# The impact of delayed treatment of uncomplicated *P*. *falciparum* malaria on progression to severe malaria: A systematic review and a pooled multicentre individual-patient meta-analysis

**DOI:** 10.1371/journal.pmed.1003359

**Published:** 2020-10-19

**Authors:** Andria Mousa, Abdullah Al-Taiar, Nicholas M. Anstey, Cyril Badaut, Bridget E. Barber, Quique Bassat, Joseph D. Challenger, Aubrey J. Cunnington, Dibyadyuti Datta, Chris Drakeley, Azra C. Ghani, Victor R. Gordeuk, Matthew J. Grigg, Pierre Hugo, Chandy C. John, Alfredo Mayor, Florence Migot-Nabias, Robert O. Opoka, Geoffrey Pasvol, Claire Rees, Hugh Reyburn, Eleanor M. Riley, Binal N. Shah, Antonio Sitoe, Colin J. Sutherland, Philip E. Thuma, Stefan A. Unger, Firmine Viwami, Michael Walther, Christopher J. M. Whitty, Timothy William, Lucy C. Okell

**Affiliations:** 1 MRC Centre for Global Infectious Disease Analysis, Department of Infectious Disease Epidemiology, Imperial College London, London, United Kingdom; 2 School of Community & Environmental Health, College of Health Sciences, Old Dominion University, Norfolk, Virginia, United States of America; 3 Global Health Division, Menzies School of Health Research and Charles Darwin University, Darwin, Northern Territory, Australia; 4 Division of Medicine, Royal Darwin Hospital, Darwin, Northern Territory, Australia; 5 Unité de Biothérapie Infectieuse et Immunité, Institut de Recherche Biomédicale des Armées, Brétigny-sur-Orge, France; 6 Unité des Virus Emergents (UVE: Aix-Marseille Univ—IRD 190—Inserm 1207—IHU Méditerranée Infection), Marseille, France; 7 QIMR Berghofer Medical Research Institute, Brisbane, Queensland, Australia; 8 ISGlobal, Hospital Clínic, Universitat de Barcelona, Barcelona, Spain; 9 Centro de Investigação em Saúde de Manhiça (CISM), Maputo, Mozambique; 10 ICREA, Barcelona, Spain; 11 Pediatric Infectious Diseases Unit, Pediatrics Department, Hospital Sant Joan de Déu (University of Barcelona), Barcelona, Spain; 12 Consorcio de Investigación Biomédica en Red de Epidemiología y Salud Pública (CIBERESP), Madrid, Spain; 13 Section of Paediatric Infectious Disease, Department of Infectious Disease, Imperial College London, United Kingdom; 14 Ryan White Center for Pediatric Infectious Disease and Global Health, Department of Pediatrics, Indiana University School of Medicine, Indianapolis, Indiana, United States of America; 15 Faculty of Infectious and Tropical Diseases, London School of Hygiene & Tropical Medicine, London, United Kingdom; 16 Sickle Cell Center, Department of Medicine, University of Illinois at Chicago, Chicago, Illinois, United States of America; 17 Medicines for Malaria Venture, Geneva, Switzerland; 18 Université de Paris, MERIT, IRD, Paris, France; 19 Department of Paediatrics and Child Health, Makerere University School of Medicine, Kampala, Uganda; 20 Imperial College London, Department of Life Sciences, London, United Kingdom; 21 Centre for Global Public Health, Institute of Population Health Sciences, Barts & The London School of Medicine & Dentistry, London, United Kingdom; 22 Institute of Immunology and Infection Research, School of Biological Sciences, University of Edinburgh, Edinburgh, United Kingdom; 23 Macha Research Trust, Choma, Zambia; 24 Department of Child Life and Health, University of Edinburgh, United Kingdom; 25 Department of Respiratory Medicine, Royal Hospital for Sick Children, Edinburgh, United Kingdom; 26 Institut de Recherche Clinique du Bénin (IRCB), Cotonou, Benin; 27 Medical Research Council Unit, Fajara, The Gambia at the London School of Hygiene and Tropical Medicine, Fajara, The Gambia; 28 Infectious Diseases Society Sabah-Menzies School of Health Research Clinical Research Unit, Kota Kinabalu, Sabah, Malaysia; 29 Gleneagles Hospital, Kota Kinabalu, Sabah, Malaysia; Mahidol-Oxford Tropical Medicine Research Unit, THAILAND

## Abstract

**Background:**

Delay in receiving treatment for uncomplicated malaria (UM) is often reported to increase the risk of developing severe malaria (SM), but access to treatment remains low in most high-burden areas. Understanding the contribution of treatment delay on progression to severe disease is critical to determine how quickly patients need to receive treatment and to quantify the impact of widely implemented treatment interventions, such as ‘test-and-treat’ policies administered by community health workers (CHWs). We conducted a pooled individual-participant meta-analysis to estimate the association between treatment delay and presenting with SM.

**Methods and findings:**

A search using Ovid MEDLINE and Embase was initially conducted to identify studies on severe *Plasmodium falciparum* malaria that included information on treatment delay, such as fever duration (inception to 22nd September 2017). Studies identified included 5 case–control and 8 other observational clinical studies of SM and UM cases. Risk of bias was assessed using the Newcastle–Ottawa scale, and all studies were ranked as ‘Good’, scoring ≥7/10. Individual-patient data (IPD) were pooled from 13 studies of 3,989 (94.1% aged <15 years) SM patients and 5,780 (79.6% aged <15 years) UM cases in Benin, Malaysia, Mozambique, Tanzania, The Gambia, Uganda, Yemen, and Zambia. Definitions of SM were standardised across studies to compare treatment delay in patients with UM and different SM phenotypes using age-adjusted mixed-effects regression. The odds of any SM phenotype were significantly higher in children with longer delays between initial symptoms and arrival at the health facility (odds ratio [OR] = 1.33, 95% CI: 1.07–1.64 for a delay of >24 hours versus ≤24 hours; p = 0.009). Reported illness duration was a strong predictor of presenting with severe malarial anaemia (SMA) in children, with an OR of 2.79 (95% CI:1.92–4.06; p < 0.001) for a delay of 2–3 days and 5.46 (95% CI: 3.49–8.53; p < 0.001) for a delay of >7 days, compared with receiving treatment within 24 hours from symptom onset. We estimate that 42.8% of childhood SMA cases and 48.5% of adult SMA cases in the study areas would have been averted if all individuals were able to access treatment within the first day of symptom onset, if the association is fully causal. In studies specifically recording onset of nonsevere symptoms, long treatment delay was moderately associated with other SM phenotypes (OR [95% CI] >3 to ≤4 days versus ≤24 hours: cerebral malaria [CM] = 2.42 [1.24–4.72], p = 0.01; respiratory distress syndrome [RDS] = 4.09 [1.70–9.82], p = 0.002). In addition to unmeasured confounding, which is commonly present in observational studies, a key limitation is that many severe cases and deaths occur outside healthcare facilities in endemic countries, where the effect of delayed or no treatment is difficult to quantify.

**Conclusions:**

Our results quantify the relationship between rapid access to treatment and reduced risk of severe disease, which was particularly strong for SMA. There was some evidence to suggest that progression to other severe phenotypes may also be prevented by prompt treatment, though the association was not as strong, which may be explained by potential selection bias, sample size issues, or a difference in underlying pathology. These findings may help assess the impact of interventions that improve access to treatment.

## Abstract 2

In a systematic review and meta-analysis, Andria Mousa and colleagues investigate associations between treatment delays and outcomes in severe malaria.

## Author summary

**Why was this study done?:**

- In 2018, severe malaria was responsible for an estimated 405,000 deaths worldwide. Patient access to first-line antimalarials in health facilities remains suboptimal in many endemic areas.
- The contribution of delay to treatment of uncomplicated malaria is often believed to be a risk factor for developing severe malaria, but this relationship has not been systematically quantified, and findings are not consistent across all studies. Understanding this relationship is critical to determine how quickly patients need to receive treatment and to quantify the impact of treatment interventions.

**What did the researchers do and find?:**

- We conducted a pooled individual-participant meta-analysis to estimate the association between delay from onset of symptoms to seeking treatment and risk of presenting with different types of severe malaria rather than uncomplicated malaria.
- The risk of severe disease was significantly higher in children and adults who had longer delays from symptom onset to treatment-seeking, and this relationship was the strongest for progression to severe malarial anaemia.
- We estimate that almost half of the severe anaemia cases in both children and adults could be prevented if they presented within the first 24 hours of symptom onset.

**What do these findings mean?:**

- The findings of this individual-participant data (IPD) meta-analysis highlight the importance of improving access to prompt first-line treatment in preventing severe malarial anaemia cases and reducing the need for potentially harmful blood transfusions.
- Our findings highlight that provision of timely treatment is essential in preventing severe disease and death.
- Providing quantitative setting-specific estimates of the benefits of timely treatment through programs such as community health workers or strengthened health systems is essential in guiding their implementation and in determining the best allocation of resources amongst all malaria interventions.

## Introduction

Access to prompt and effective antimalarial treatment for uncomplicated *Plasmodium falciparum* malaria is key in preventing progression to severe complications and death. In 2018, an estimated 405,000 deaths were attributed to severe malaria (SM) worldwide, with 67% of them occurring in children aged under 5 years [1]. Access to treatment varies greatly by country [2], and for some countries, only a small proportion of all severe cases gain admission to hospital [3]. Implementation, scale-up, and maintenance of integrated community case management (iCCM) require significant resources, estimated at US$3.4 billion per year across sub-Saharan Africa [4,5]. Recent reports from the Rapid Access Expansion (RACE) programs in Nigeria have shown that care-seeking for fever from an appropriate provider increased from 78% to 94% following implementation of iCCM and that coverage with artemisinin-based combination therapies (ACTs) within 1 day of symptom onset increased from 57% to 74% amongst malaria-confirmed cases [6]. Evidence from these iCCM programs have shown a reduction of overall clinical disease symptoms and all-cause child mortality after implementation [6,7]. These are consistent with findings from other studies, including cluster randomised trials of community health worker (CHW) programs, showing a decrease in overall mortality after increasing diagnosis and treatment coverage for malaria and other diseases [8–10]. However, malaria-specific effects have not been systematically quantified, despite anecdotal reports that improved access to quick and effective treatment reduces severe disease [11]. Understanding treatment-seeking pathways and the effect of prompt access to treatment on progression to severe disease is important in informing how malaria control programmes allocate resources between preventive interventions, such as vector control and seasonal chemoprevention, and interventions that improve access to treatment.

Some of the most widely studied clinical manifestations, symptoms, or phenotypes of severe disease include severe malarial anaemia (SMA), cerebral malaria (CM), and respiratory distress syndrome (RDS). Such presentations of severe disease are not mutually exclusive because a single individual may present with multiple symptoms. Clinical manifestations of severe disease are dependent on age and transmission setting [12–15]. In high-transmission areas where the average age at first infection is lower, severe disease often manifests as SMA, which is common in younger children, whereas CM is mostly concentrated in older children and adolescents and is more likely to occur in areas of low-to-moderate transmission [13,16]. When compared with other types of severe disease, CM has a higher case fatality rate, reaching up to 25% [17,18].

Recent evidence from national surveys suggests that treatment by trained medical providers is only sought for 37% of febrile children, and in many cases, patients do not receive first-line antimalarial treatment according to national policy [1,2,19]. This is attributed to poor access to formal healthcare providers and the distribution of ineffective drugs by the private sector or unqualified providers. Findings on the relationship between delay in receiving appropriate treatment for uncomplicated malaria (UM) and risk of developing severe disease are not consistent across all studies [20]. Several studies in both African and non-African settings have reported an increased risk of SM with delay [21–28]. By contrast, some studies found no difference in duration of symptoms between severe and uncomplicated disease [29–32], but single-site studies of SM frequently suffer from small case denominators and reduction of statistical power in analyses stratified by SM phenotype.

Inconsistencies amongst studies in estimating the association between prompt treatment in preventing severe disease might therefore be explained by factors such as the relative numbers and proportions of different severe disease syndromes in different settings, inconsistent exclusion of other causes of disease, the inclusion of different age groups and sample size. Here, we conducted a pooled individual-patient data (IPD) analysis, standardising definitions across multiple studies, with the primary objective of estimating the association between delay to treatment and disease outcomes. This was done by comparing delay to treatment in a) SM versus UM cases and b) between cases with different types of severe disease manifestations. We also explored other aspects of the treatment-seeking pathway, such as travelling time, distance to the health facility, and preadmission treatment history.

## Methods

### Study inclusion and procedures

A comprehensive search of 2 databases, Ovid MEDLINE and Embase, was initially conducted to identify studies of SM (inceptions to 22nd September 2017). Details of the search strategy, as well as the inclusion and exclusion criteria, are found in S1 Text (Table A and Table B, respectively). In brief, we included case–control studies of uncomplicated and severe *P*. *falciparum* malaria with available data on delay to treatment, quantified as either duration of illness or fever prior to hospital admission. Large studies of severe disease that did not have a case–control design, such as cohort studies, were still considered if they were sufficiently large (n > 100) to allow comparisons between different severe disease types. The research questions were formed as part of an MRC UKRI project, and the protocol is registered with protocols.io at: https://dx.doi.org/10.17504/protocols.io.bgzfjx3n. No data-driven changes occurred to the analysis plan, and both research objectives and methods remained unchanged.

Authors of the identified studies were contacted to request individual-level data (S1 Data and S2 Data). Studies that reported an odds ratio (OR) for the relationship between delay to treatment and SM but were not analysed because of nonresponse are summarised in S1 Table. In most studies for which individual-level data were obtained, each participant was only included once, and in the case of readmissions, only data from the first admission were retained and analysed. The only exception to this was the Tanzanian studies, which excluded new entries within 6 weeks of discharge. Clinical definitions of SM, which depended on time of publication and setting [33–35], were standardised across the studies, based on the WHO 2014 definition [35] (S2 Text). UM and SM cases were reclassified according to that definition, and cases that were classified as severe in the original study were excluded if they did not meet the new criteria. An additional UK data set on travellers and imported cases was used to compare the results of the pooled analysis to those from a setting with high access to treatment [36].

Findings are reported in accordance with the Preferred Reporting Items for Systematic Reviews and Meta-Analyses (PRISMA) checklist of items specific to IPD meta-analyses (S1 Checklist) [37]. Risk of bias was assessed using a modified version of the Newcastle–Ottawa scale for assessing bias in nonrandomised studies (S2 Checklist) [38]. Newcastle–Ottawa quality assessment scale (NOS) ratings were converted to Agency for Healthcare Research and Quality (AHRQ) standards scale, categorising the quality of studies as good, fair, or poor (thresholds shown in S2 Checklist). Publication bias was not assessed across publications because not all studies reported an OR for the association between treatment delay and presentation with severe disease. Although ORs for this relationship were reported in some of the studies (S1 Table), it was not done in a consistent manner between studies (for example, comparator groups were different durations of delay or adjustment for different factors), which does not enable a funnel plot analysis.

### Ethics statement

All studies included were approved by an institutional ethics review committee (listed in S3 Text for individual studies) and obtained informed consent from all participants. Ethics approval was not required for the present study.

### Statistical analysis

Studies without a matched case–control design were not matched but were analysed as an entire series. Age-adjusted, mixed-effects multivariable logistic regression was used to quantify the effect of covariates on the odds of severe disease, specific SM phenotypes, or mortality, accounting for random effects amongst studies. A mixed-effects linear regression was used to explore the change in haemoglobin at admission with increasing delay. The odds of receiving a blood transfusion during hospital admission was also explored with increasing treatment delay. For the main analyses, in addition to ORs, risk ratios (RRs) and associated 95% CIs are also reported for ease of subsequent generalisation by readers. These were obtained using a generalised estimating equations (GEE) model, allowing for correlation of observations within studies. All analyses were done separately for children aged <15 years and for ages 15 and over. A separate subset analysis included young children aged between 6 months to 5 years (excluding <6-month–olds to minimise any biases that may arise from differences in case definitions between infants and children). In all analyses, a likelihood ratio test was performed to compare the difference between a model with and without the term for illness duration.

We explored confounding by education of the caregiver (less than primary versus at least primary) and effect modification by transmission intensity using estimates of parasite prevalence of *P*. *falciparum* in 2- to 10-year–olds (PR_2–10_) obtained from the Malaria Atlas Project database at the upper administrative area level for the year of admission for each individual [39]. Transmission intensity was classified as low (*Pf*PR_2–10_ < 10%), moderate (*Pf*PR_2–10_ ≥ 10% to <35%) and high (*Pf*PR_2–10_ ≥ 35%) according to WHO criteria [40]. Accuracy of reporting the time of onset of the first uncomplicated symptoms was also examined by restricting main analyses to studies specifically reporting duration of fever (the most common uncomplicated symptom) instead of less specific definitions such as ‘duration of illness’. Some of the sites included were referral sites, and it is likely that presentation with severe disease may be associated with initial treatment failure. To account for bias arising from subjects who did not present directly to the clinic from which they were recruited, a subset analysis including only those who reported no prior antimalarial treatment was performed. Studies included were classified as pre-ACT and post-ACT based on first-line antimalarial treatment policies of each country. A likelihood ratio test was performed to test for the presence of an interaction between this classification and duration of illness.

Further sensitivity analysis was undertaken to look at the association between treatment delay and severe disease separately for those who live close to the health facility (defined as a value lower than the median travelling time or median distance to the hospital). We looked for selection bias in attendance at the health facility by comparing the odds of UM and different severe phenotypes with increasing travelling time to the hospital. Travelling time to the hospital was self-reported by the patient or caregiver, measured by a field assistant, or estimated based on distance to the health centre (using AccessMod [41]). The Global Rural-Urban Mapping project (GRUMPv1) was used to classify individuals as living within a rural or urban area based on their home location [42,43]. Because this was only available for 1 study [13], we obtained information on coordinates of the hospital sites for all studies included and used year-specific WorldPop estimates of mean population density per squared kilometer adjusted to match official UN population estimates (accessible here: https://www.worldpop.org/geodata/listing?id=77). We quantified a mean population density for a catchment area, using a radius of 30 km and 35 km around the hospital site [44], and a mean population density cutoff of >300 per km^2^ was used to stratify the main analyses [45]. We tested for an interaction between duration of illness and urban/rural or population density classifications on any severe disease and SMA.

The overlap between different SM phenotypes, i.e., when patients had 2 or more different severe phenotypes, was investigated using pairwise phi coefficients. Analyses were also repeated in patients with only 1 phenotype to examine the effect of overlap, and the number of phenotypes present in an individual was also investigated with increasing delay to treatment. For the UK data, level of immunity was also adjusted for and was defined as low for UK travellers returning from malaria-endemic countries, partial for those of African origin visiting friends and family in endemic countries, and high for new entrants from endemic countries.

## Results

### Review findings and study characteristics

A total of 3,189 studies were generated by the search strategy, of which 40 met the eligibility criteria (S1 Fig). We carried out a one-stage IPD analysis that included 14 studies whose authors responded within the timeframe of our study, of which 11 were identified in the review and 3 through contacting authors. The studies were set in Benin, Malaysia, Mozambique, Tanzania, The Gambia, Uganda, the UK, Yemen, and Zambia. Data from a total of 3,989 patients with SM and 5,780 UM cases were included in the pooled analysis (Table 1, S2 Table). For 4 of the studies with a case–control design, controls (UM) and cases (SM) were matched on age and sex [21,22,30] (details of study design and matching of included studies shown in S3 Table). The quality of all studies included was ranked as ‘Good’, with all studies scoring 7 or more out of 10 on the NOS scale (S2 Checklist).

**Table 1:** Characteristics of studies used in IPD meta-analysis. Table includes study site, study period, age ranges included, and frequencies and percentages of uncomplicated and SM groups amongst the study sample. Percentages with a given phenotype amongst severe cases are shown in brackets and omit missing values for that phenotype. The denominator only includes those who were assessed for that phenotype. ‘NA’ entries indicate that information on the phenotype in that study was not collected (for example, data on RDS were not collected in the Zambian study). Percentages may add up to more than 100% because the same study subject may present with more than 1 phenotype. This table excludes 196 individuals (2% of total participants) who were classified as severe in the original studies but did not meet the criteria for severe disease in the IPD analysis. See S2 Table for other severe disease phenotypes. See S4 Table for SM frequencies from the UK study, which was not used in the pooled analysis. **Abbreviations:** CM, cerebral malaria; IPD, individual-participant data; RDS, respiratory distress syndrome; SM, severe malaria SMA, severe malarial anaemia; UM, uncomplicated malaria.

Study Site	Years	Age Range	UM (%)	SM (%)	SMA (%)	RDS (%)	CM (%)
**Cotonou, Benin [46]**	Apr 2009 to Aug 2009	4 months to 14 years	46 (51.1)	44 (48.9)	5 (55.6)	16 (38.1)	7 (16.7)
**Farafenni, The Gambia [21]**	Sept 2002 to Dec 2002	1 months to 10 years	139 (30.2)	321 (69.8)	124 (56.6)	59 (18.7)	38 (16.9)
**Serekunda, The Gambia [47]**	Aug 2007 to Jan 2011	8 months to 16 years	360 (55.0)	295 (45.0)	22 (7.8)	130 (88.4)	55 (18.8)
**Keneba, The Gambia [48]**	Nov 2009 to Apr 2012	4 months to 5 years	31 (83.8)	6 (16.2)	1 (20.0)	NA	0 (0.0)
**Sabah, Malaysia [23]**	Sept 2010 to Nov 2012	13 years to 78 years	175 (89.7)	20 (10.3)	2 (10.0)	4 (22.2)	2 (12.5)
**Manhiça, Mozambique [30]**	Apr 2006 to Nov 2006	2 months to 5 years	63 (46.3)	73 (53.7)	24 (32.9)	31 (42.5)	7 (9.6)
**Manhiça, Mozambique**	Sept 2014 to May 2016	under 10 years	55 (37.2)	93 (62.8)	29 (31.2)	13 (14.0)	16 (17.2)
**Kilimanjaro and Tanga, Tanzania [13]**	Feb 2002 to Aug 2002	All ages	2,941 (67.8)	1,399 (32.2)	819 (59.7)	220 (15.9)	171 (12.7)
**Tanga, Tanzania [49]**	Jun 2006 to May 2007	2 months to 13 years	1,368 (63.2)	798 (36.8)	413 (51.8)	239 (30.0)	92 (11.5)
**Kampala, Uganda [50]**	2003 to 2008	2 years to 15 years	90 (51.4)	85 (48.6)	NA	NA	85 (100.0)
**Kampala, Uganda [51]**	2008 to 2013	1 year to 11 years	0 (0.0)	494 (100.0)	289 (58.5)	134 (27.1)	262 (53.0)
**Taiz, Yemen [22]**	Nov 2002 to Aug 2004	6 months to 10 years	445 (63.8)	253 (36.2)	101 (39.9)	136 (53.8)	19 (7.5)
**Macha, Southern Province, Zambia [52]**	Mar 2001 to May 2005	5 months to 7 years	67 (38.3)	108 (61.7)	72 (66.7)	NA	33 (30.6)
**Total**			**5,780 (59.2)**	**3,989 (40.8)**	**1,901 (51.0)**	**982 (27.1)**	**787 (20.5)**

**Fig 1:**
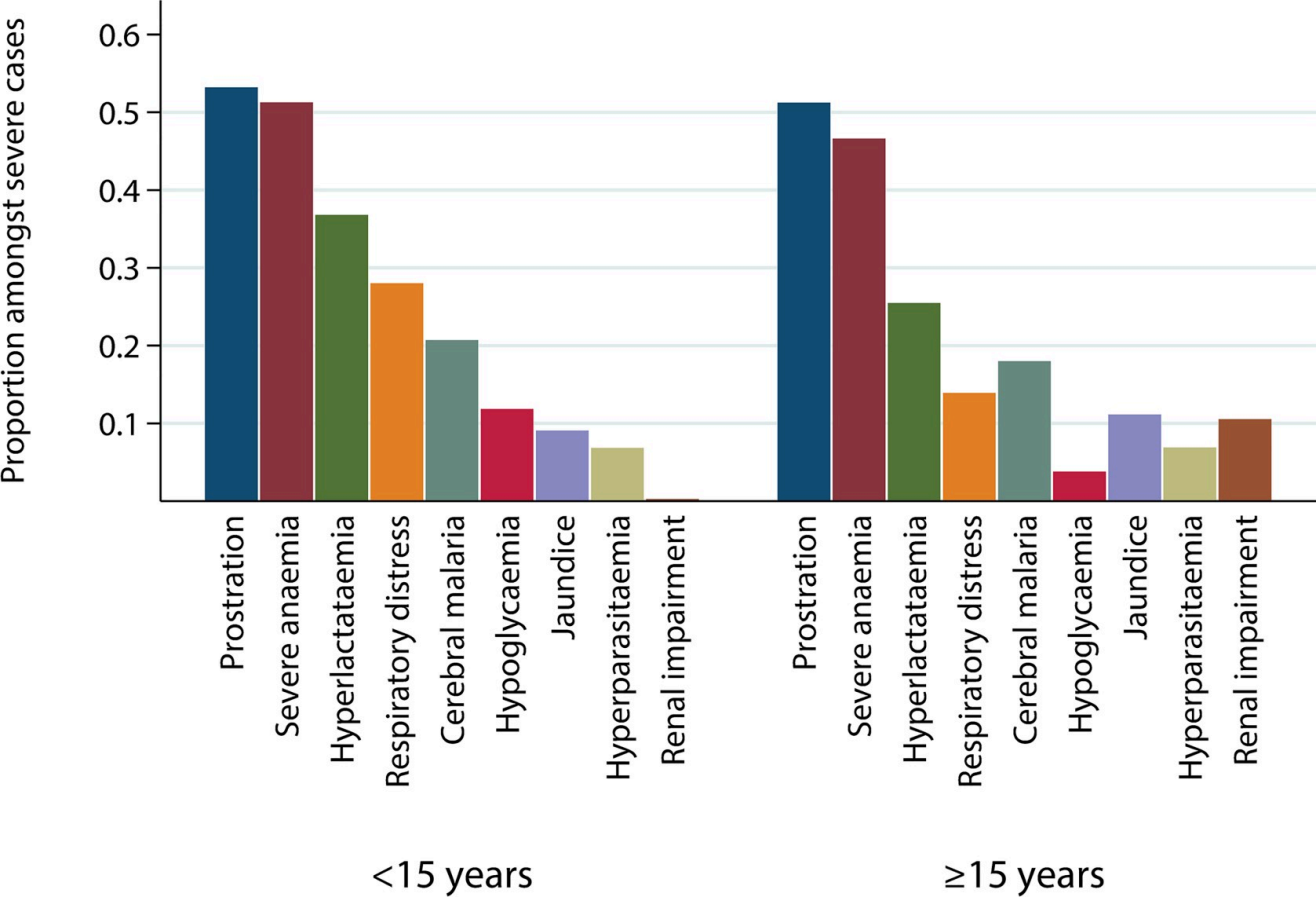
Prevalence of SM phenotypes amongst severe cases by age group. Proportions were calculated for severe cases with no missing values for a given measure. Information for all phenotypes was not available in all studies (Table 1, S2 and S4 Tables). Each case can present with more than 1 phenotype. SM, severe malaria.

Children aged under 15 constituted 79.6% (N = 4,596) of uncomplicated cases and 94.1% (N = 3,754) of severe cases (S5 Table). The most common severe disease manifestations were prostration (53.1%), SMA (51.0%), hyperlactataemia/acidosis (36.1%), RDS (27.1%), and CM (20.5%) (Fig 1). RDS was relatively common in those with hyperlactataemia/acidosis (phi coefficient = 0.24; see S6 Table for all associations). The distribution of severe disease symptoms varied between study sites (S2 Fig) and by age (Fig 1). In areas of moderate-to-high transmission intensity, such as in Farafenni in The Gambia (2002) and certain areas in Tanzania, most cases were younger children and SMA and prostration were the most common severe disease types, as expected. Jaundice was only common in low-transmission settings and in older populations, such as in Malaysia. In endemic settings, renal impairment was only seen in 0.8% (3/395) of severe cases who were assessed for creatinine or blood urea levels. This was not the case in the UK study population, in which most (43.8%, 74/169) individuals presented with renal impairment and a small number presented with SMA (2.4%, 4/168).

**Fig 2:**
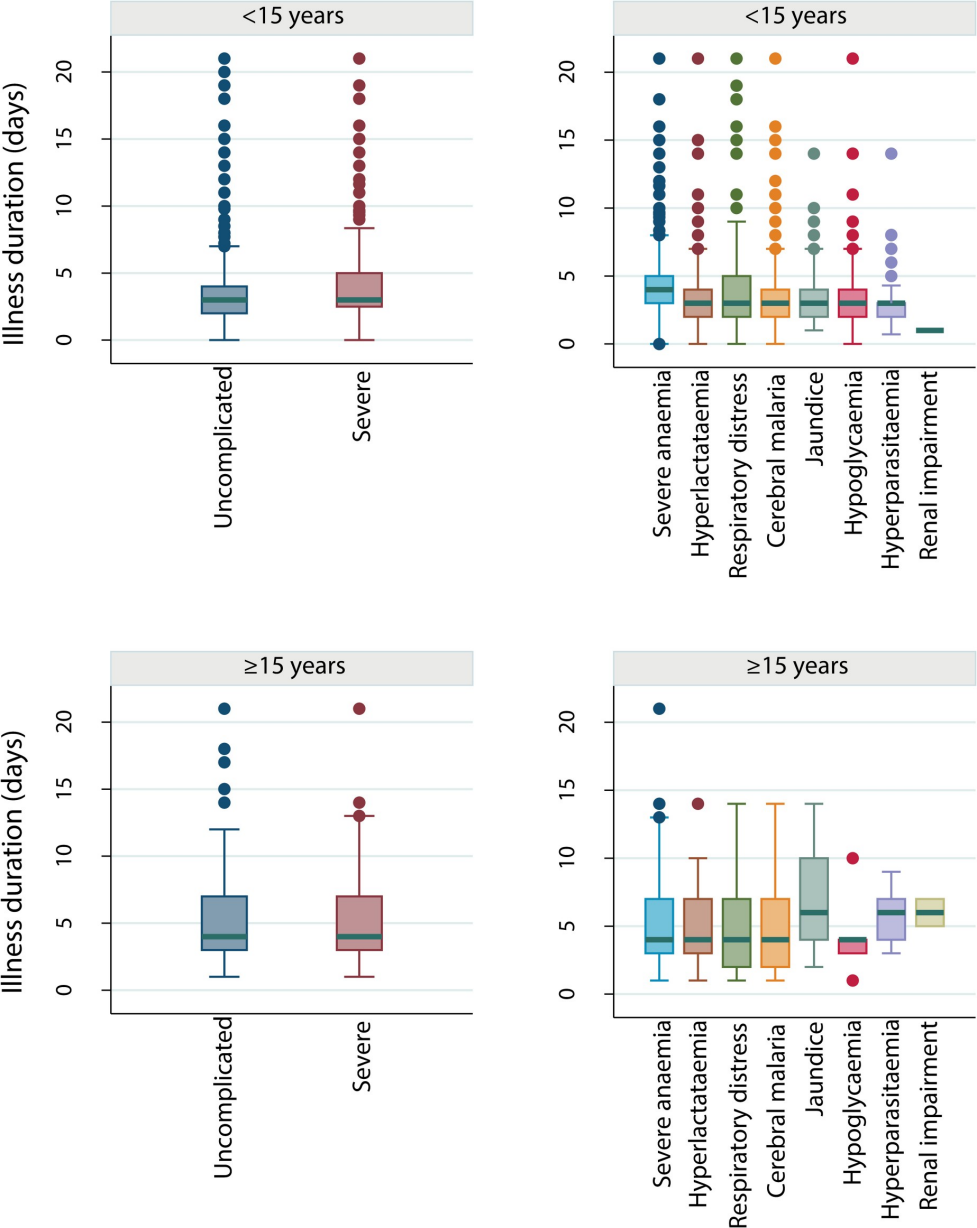
Illness duration (in days) prior to arrival at the health facility by SM phenotype and age group. Box-and-whisker plots showing median and IQR of duration of illness/fever in children and adults stratified by severity group (ages <15 years: N_UM_ = 3,277, N_SM_ = 3,708; ages ≥15 years: N_UM_ = 300, N_SM_ = 226). Outliers (observations that are 1.5 × IQR from the lower or upper quartiles) are denoted. Extreme outliers, defined as duration of illness of over 3 weeks, are omitted from these plots (0.6% of cases [20 UM and 25 SM] were omitted). SM, severe malaria; UM, uncomplicated malaria.

Data on time since first fever or any symptoms prior to arrival at the study health facility, as reported by the patient or caregiver, were available for a total of 7,512 individuals (3,577 UM and 3,935 SM; distribution shown in S3 Fig, S4 Fig, and Fig 2). Reported duration of illness varied by study and age group from a median of 1 to 5 days (S4 Fig and S5 Fig). Data on duration of severe symptoms were also obtained from 5 studies, including information on duration of coma, unconsciousness, convulsions, respiratory distress, fast breathing, or difficulty in breathing (N = 1,323). Care was sought within the first day of severe symptom onset for most cases because the median delay was <1 day after onset of any severe symptom (S7 Table). However, onset of fever or other symptoms of uncomplicated illness for those who later developed SM occurred, on average, between 1 to 3 days before they developed signs of severe disease, depending on the symptoms (S7 Table).

### Association of delay to treatment with severe malarial disease

**Fig 3:**
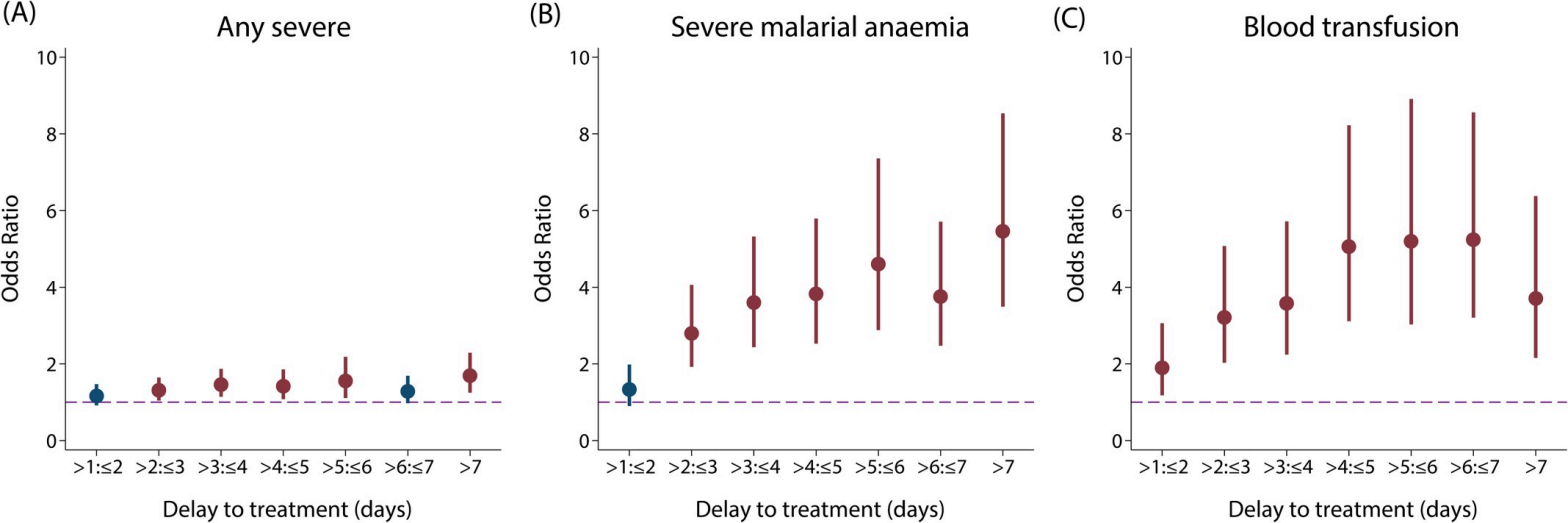
**Treatment delay and odds of presenting with any SM (A), SMA (B), and receiving blood transfusion (C) in children under 15.** ORs (and 95% CIs) for presentation with severe disease rather than UM (A & B) with each additional reported day of delay after initial symptoms, compared with patients receiving treatment within 1 day of symptom onset (N_UM_ = 3,277, N_SM_ = 3,708, N_SMA_ = 1,774). Amongst 5 studies with information on blood transfusions during hospital admission, 27.7% (1,520/5,496) of children aged under 15 with available data had received a blood transfusion. ORs (and 95% CIs) for receiving a blood transfusion was estimated for each additional day of illness duration amongst all uncomplicated and severe cases. All ORs shown were obtained from a mixed-effects logistic regression adjusted for age as a linear predictor and allowed for random study effects. Statistically significant ORs are denoted in red (dashed purple line: OR = 1). OR, odds ratio; SM, severe malaria; SMA, severe malarial anaemia; UM, uncomplicated malaria.

**Fig 4:**
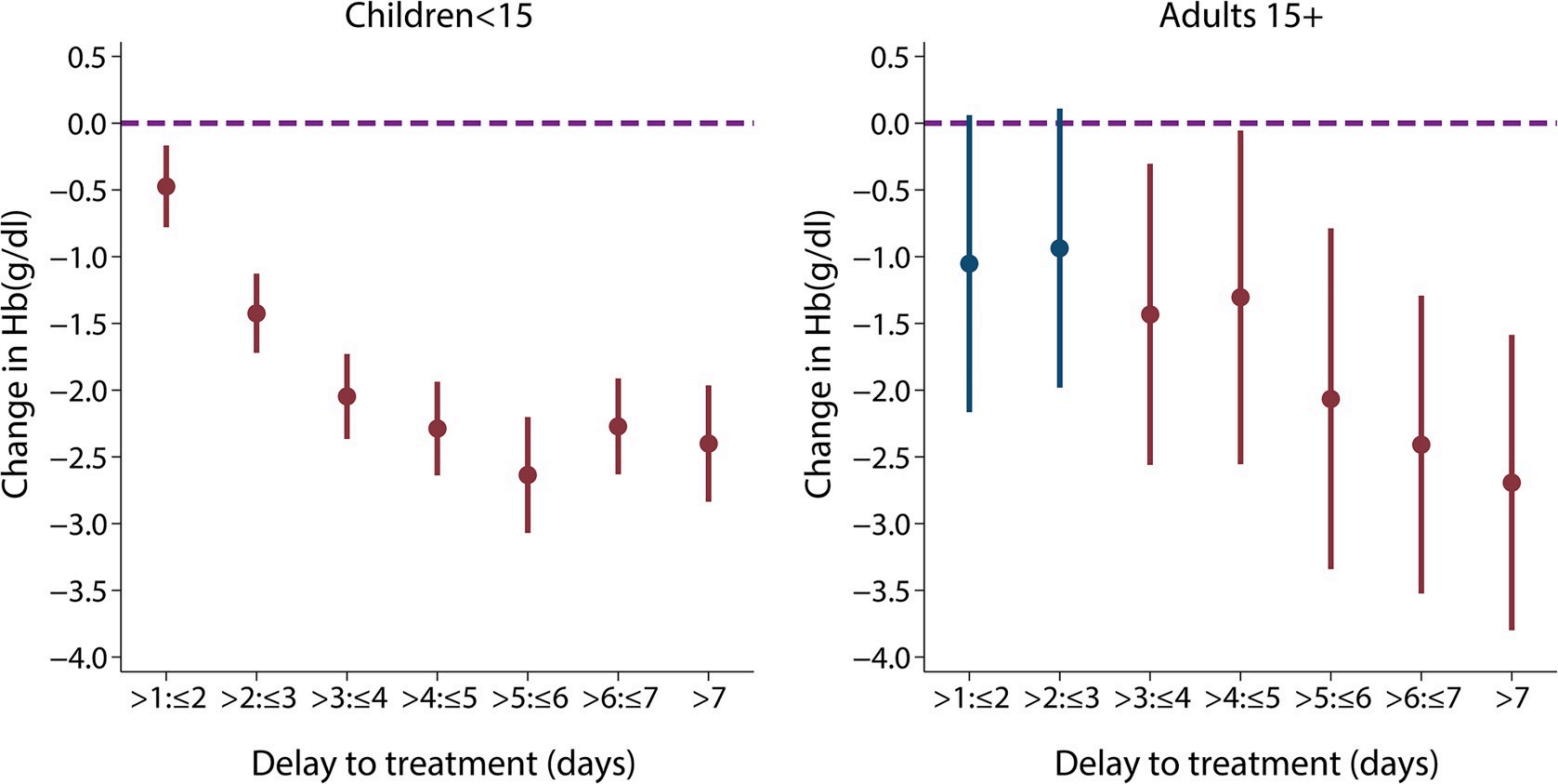
**Treatment delay and change in Hb levels for (A) children aged under 15 and (B) adults aged 15 years and over.** The change in Hb in g/dl for each extra day of delay compared with Hb in those treated within 24 hours. An increase in delay was associated with decrease in Hb levels (children: likelihood ratio χ^2^_(7)_ = 361.16, p < 0.001; adults: likelihood ratio χ^2^_(7)_ = 41.62, p < 0.001). Hb was recorded for 5,908 individuals in 10 of the studies, and its concentration was normally distributed. A mixed-effects general linear model was used to examine the relationship between delay and Hb levels, irrespective of severity group. On average, children who were admitted to the hospital more than 3 days after symptom onset had a reduction in Hb of at least 2 g/dl compared with those receiving treatment in the first day after illness onset. Hb, haemoglobin.

In children, the adjusted odds of any type of severe disease were significantly higher in patients with longer delays between initial symptoms and arrival at the study health facility after accounting for age and random study effects (OR [95% CI] = 1.33 [1.07–1.64] for a delay of over 24 hours compared with treatment within 1 day in children <15, p = 0.009; Fig 3). When stratifying by different severe disease manifestations, reported duration of illness was a strong predictor of presenting with SMA in children under 15 years (Fig 3; see S8 Table for RRs). Compared with receiving treatment within 24 hours of symptom onset, the OR for presenting with SMA was 2.79 (95% CI: 1.92–4.06, p < 0.001) for a delay between 2 to 3 days and 5.46 (95% CI: 3.49–8.53, p < 0.001) for a delay of over 7 days (χ^2^_(7)_ = 145.38, p < 0.001). The ORs for presenting with SMA in the subset analysis including only children aged between 6 months and 5 years were slightly higher (delay of 2 to 3 days: OR = 3.18 [95% CI: 2.12–4.79, p < 0.001]; delay of over 7 days: OR = 6.18 [95% CI: 3.80–10.05, p < 0.001]; χ^2^_(7)_ = 133.14, p < 0.001; S6 Fig). For the same delay groups, the equivalent reduction in haemoglobin levels in children under 15 years, irrespective of severity group, was 1.46 g/dl (95% CI: 1.19–1.73, p < 0.001) and 2.13 g/dl (95% CI: 1.73–2.53, p < 0.001), respectively (χ^2^_(7)_ = 361.16, p < 0.001; Fig 4). Similar trends were observed for the odds of receiving a blood transfusion with increasing delay to treatment in children <15 years (χ^2^_(7)_ = 120.95, p < 0.001; Fig 3) or young children <5 years (χ^2^_(7)_ = 109.96, p < 0.001; S6 Fig). Compared with those arriving within the first day of symptoms, the odds of receiving a blood transfusion when arriving at the hospital 4 to 5 days after symptom onset were 5.06 (95% CI: 3.11–8.22, p < 0.001) times higher in children under 15 years and 4.77 (95% CI: 2.87–7.95, p < 0.001) times higher in children under 5 years. For ages 15 or over, the odds of SMA were only associated with delays of over 4 days (χ^2^_(4)_ = 22.57, p < 0.001; S7 Fig). If the associations between delay and odds of SMA were entirely causal, we estimate that treating everyone in the study populations within 24 hours after symptom onset would have averted 42.8% of SMA cases in children under 15, 46.8% of SMA cases in children under 5, and 48.5% in adults aged 15 or over. In the UK study of imported adult cases, similar results were observed to those in adults from the pooled analysis (S7 Fig), with the odds of any severe disease being 3–4 times higher in those with a delay of 5 to 7 days compared with those being admitted in the first 24 hours after symptom onset (χ^2^_(7)_ = 17.59, p = 0.014). In this study, case numbers of individual phenotypes were too small to separate out.

**Fig 5:**
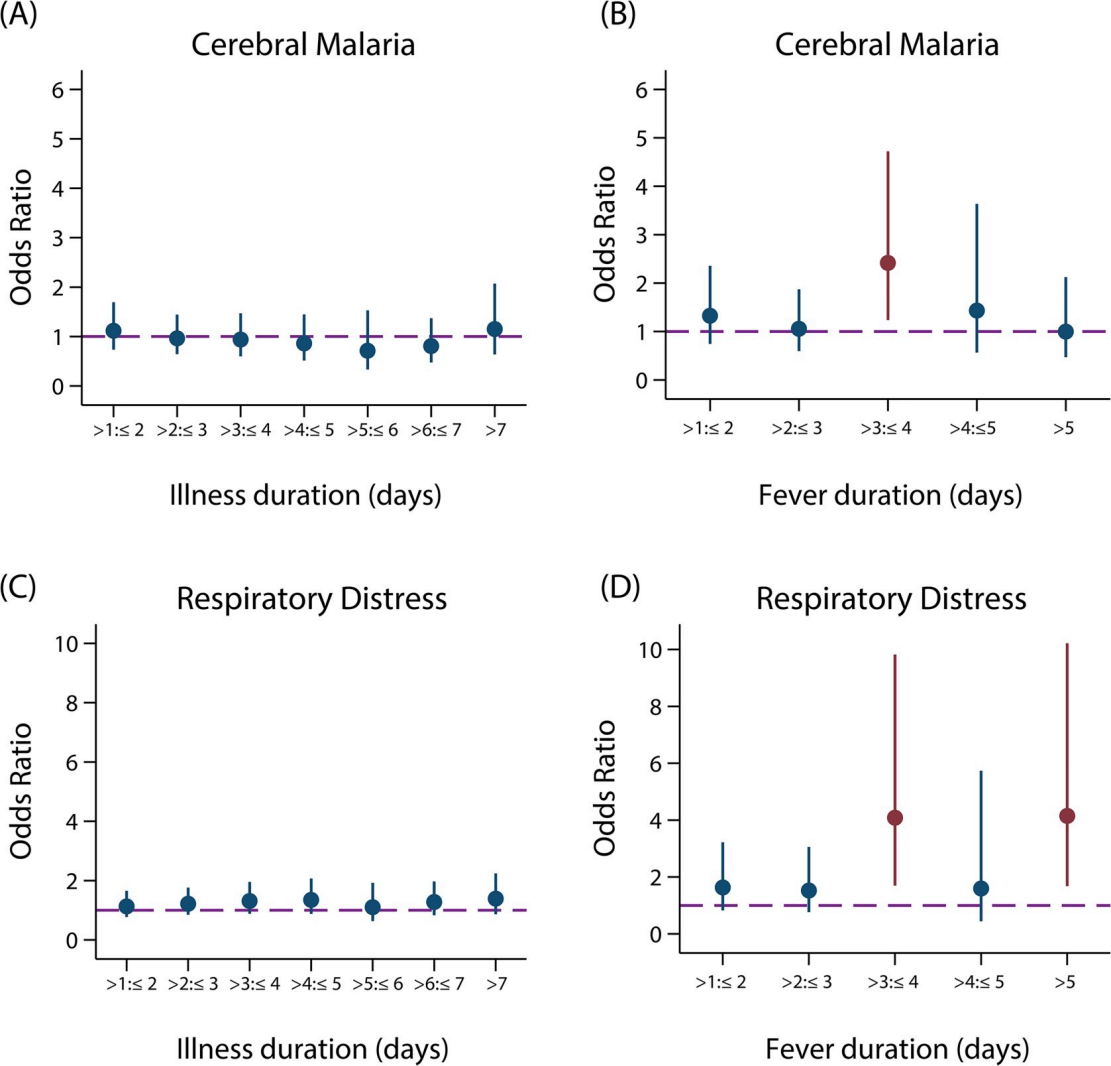
**Treatment delay and odds of presenting with CM (A–B) and respiratory distress (C–D) in children <15 years.** ORs (and 95% CIs) for presentation with severe disease with each additional reported day of illness before attending the health facility compared with patients attending within 1 day of reported illness onset (A and C; N_UM_ = 3,277, N_CM_ = 737, N_RDS_ = 945) or fever onset (B and D; N_UM_ = 492, N_CM_ = 441, N_RDS_ = 251). ORs were obtained from a mixed-effects logistic regression adjusted for age as a linear predictor and allowed for random study effects. Statistically significant ORs are denoted in red (dashed purple line: OR = 1). The same plots for other SM phenotypes are shown in S9 Fig. CM, cerebral malaria; OR, odds ratio; RDS, respiratory distress syndrome; SM, severe malaria UM, uncomplicated malaria.

In the overall data set, no relationship was observed between duration of illness and presentation with any other SM phenotypes for either children or adults (Fig 5, S7 and S8 Figs). However, caregivers may have reported the time of onset of severe symptoms if asked about duration of illness rather than initial onset of UM symptoms. We therefore also repeated the analysis restricted to a subset of 9 studies that specifically recorded onset of fever, the most common UM symptom (N = 1,866, including 1,689 children aged under 15 years). In these studies, there was some evidence of an association between treatment delay after fever onset and all severe phenotypes except hyperparasitaemia (Fig 5, S9 Fig). Compared to receiving treatment within 24 hours of fever onset, the OR for a child presenting with CM and RDS for a delay of 3 to 4 days after fever onset was 2.42 (95% CI: 1.24–4.72; p = 0.01) and 4.09 (95% CI: 1.70–9.82; p = 0.002), respectively. A delay of over 4 days from fever onset was not associated with the odds of presenting with CM (>4 to ≤5 days: OR = 1.43 [95% CI: 0.56–3.63, p = 0.45], 5+ days: OR = 1.00 [95% CI: 0.50–2.12, p = 1.00] compared with a delay of ≤24 hours; Fig 5).

Sensitivity analysis accounting for mother’s educational attainment was carried out. Low mother’s education was significantly associated with SMA, RDS, and CM (OR range = 1.74–1.81) but did not affect the relationship between treatment delay and disease severity (S9 Table). To explore the influence of overlapping severe symptoms, we repeated the analysis excluding individuals with more than 1 type of symptom. The association between delay and severe disease phenotypes in the absence of other severe phenotypes remained similar as in the main analysis (S9 Table). The mean number of severe phenotypes at presentation did not increase with increasing duration of illness (χ^2^_(7)_ = 2.67, p = 0.92).

**Fig 6:**
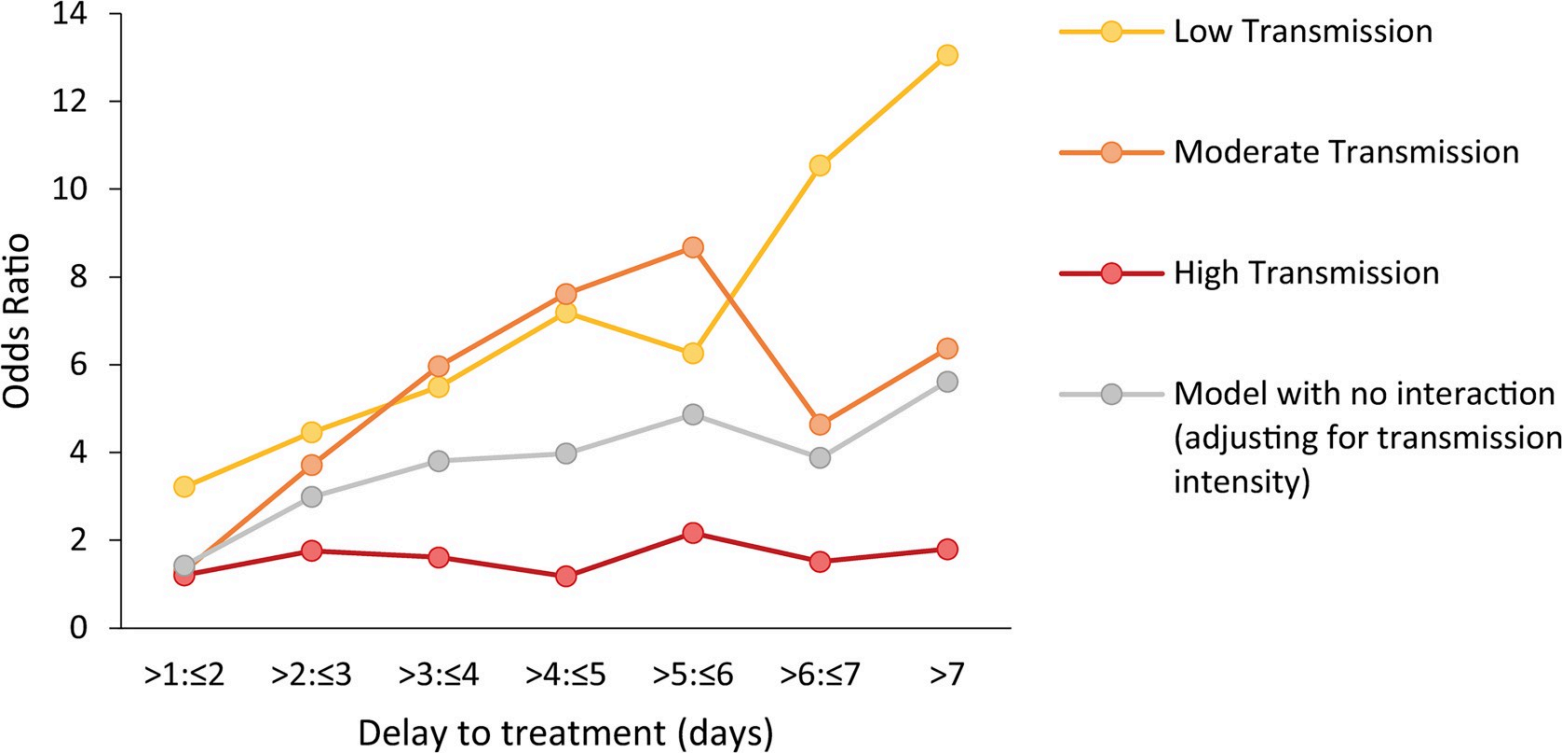
Association between delay to treatment and SMA for different levels of transmission intensity in children <15 years. ORs for the association between duration of illness and SMA from 2 age-adjusted mixed-effects models: one adjusting for transmission intensity level and one accounting for an interaction between transmission intensity and delay. Transmission intensity was categorised into low (*Pf*PR_2–10_ < 10%), moderate (*Pf*PR_2–10_ 10% to <35%), and high (*Pf*PR_2–10_ ≥ 35%). CIs around the OR estimates are shown in S10 Table. OR, odds ratio; SMA, severe malarial anaemia.

There is some evidence to suggest that the association between treatment delay and severity is stronger in areas with low malaria transmission compared to areas with high malaria transmission. There was no interaction between transmission intensity and treatment delay on the odds of CM and there was a nonlinear association with odds of RDS (likelihood ratio tests: CM: χ^2^ = 18.99, p = 0.17; RDS: χ^2^ = 48.92, p < 0.001; Fig 6, S10 Table). An almost-linear increase in the effect size of treatment delay on SMA with a decrease in transmission intensity was observed (likelihood ratio test: χ^2^ = 49.86, p < 0.001; Fig 6, S10 Table). Compared with receiving treatment within the first day the OR for presenting SMA for a delay of 4 to 5 days was 7.19 (95% CI: 2.38–21.73; p < 0.001) in those living in areas of low transmission (<10% *Pf*PR_2–10_). In areas of high malaria transmission intensity (≥35% *Pf*PR_2–10_), the equivalent OR was smaller and not statistically significant (OR = 1.18, 95% CI: 0.10–13.68). Nonetheless, the proportion of SMA amongst severe cases was much higher in high malaria transmission settings compared with low and moderate transmission intensities (<10% *Pf*PR_2–10_ = 0.39; 10% to <35% *Pf*PR_2–10_ = 0.51; ≥35% *Pf*PR_2–10_ = 0.77). Mean haemoglobin, even in children with UM, was much lower in areas with high malaria transmission intensity (6.41 g/dl, 95% CI: 6.31–6.51 for ≥35% *Pf*PR_2–10_) compared with areas of lower malaria transmission intensity (9.78 g/dl, 95%CI: 9.54–10.01 for <10% *Pf*PR_2–10_; p < 0.001), suggesting there might be higher levels of pre-existing anaemia in these populations.

Mortality outcomes during admission were available for 2,921 UM and 3,811 SM cases. Mortality was 1.0% in the uncomplicated group and 8.3% in the severe group (S11 Table). The highest case fatality rate was observed for CM (19.6%). Although mortality was dependent on the type of severe disease (S10 Fig*)*, it was not associated with duration of illness in severe cases when adjusting for age (p > 0.05 for any day of delay for both children and adults). Mortality was higher in children with SMA compared to the uncomplicated group accounting for age (OR = 7.27, 95% CI = 4.57–11.56, p < 0.001), even with exclusion of SMA cases who also presented with other severe phenotypes (OR = 1.95, 95% CI = 1.07–3.55, p = 0.03). Amongst children with severe disease, mortality in SMA cases was lower compared with those with other severe phenotypes, adjusting for age (OR = 0.63, 95% CI = 0.49–0.82, p = 0.001). A lower proportion of individuals presenting with SMA were febrile (>37.5°C) at presentation compared with other phenotypes (56.4% febrile for SMA compared with a range of 61.1%–84.0% febrile for other phenotypes). Amongst SMA cases, a higher proportion seeking treatment quickly (within 1 day of symptom onset) had fever at presentation (63.8% versus 56.3% presenting to the hospital after the first day of symptom onset), suggesting that fever may trigger treatment-seeking more than other SMA symptoms.

Travelling time to the health facility was recorded for 2,604 UM cases and 2,579 SM cases and was strongly associated with increased odds of severe disease, though the association between geographic distance and severity was not as strong (S11 Fig and S12 Fig). A small but statistically significant positive correlation was found between duration of illness and travelling time (Spearman’s rho = 0.16, p < 0.001, N = 5,313). The odds of severe disease were approximately 2–3 times higher for those living further than 1 hour of travel time from the health facility, even after adjusting for duration of illness (S11 Fig). This suggested a selection bias, with cases living further away being less likely to travel to the health facility when the illness was not severe. The association between delay to treatment and SMA remained similar after adjustment for travelling time to the health facility (OR of SMA = 3.12 for a delay of 2 to 3 days versus ≤24 hours, 95% CI: 1.78–5.48 [p < 0.001]; not adjusted for travelling time for the same subset: OR = 3.36, 95% CI: 1.92–5.87 [p < 0.001], S13 Fig). The same conclusions were drawn when restricting the analysis to children living close to the health facility (distance < 17.2 km or travelling time <35 minutes; S13 Fig; for a delay of 2 to 3 days compared with ≤ 24 hours: OR = 3.30, 95% CI = 1.37–7.97, p = 0.008). Assuming that treatment is received within 24 hours, the reduction of SMA cases estimated from this subgroup analysis is 63.3% for children and 57.9% for adults.

Geolocated individual-level data were available for a subset of the individuals from the Tanzanian study conducted in 2002 (n = 3,992) [13]. Amongst children with available data on illness duration, 13% (219/1,706) were classified as living in an urban location. Residing in an urban location was associated with lower odds of SM compared with living in a rural location in this setting (any SM: OR = 0.39, 95% CI = 0.32–0.48, p < 0.001; SMA: OR = 0.18, 95% CI = 0.12–0.25, p < 0.001). In the stratified analysis of this subset population, the OR comparing ≤24 hours versus >24 hours for any SM was 0.94 (95% CI = 0.49–1.80, p = 0.84) for those living in rural areas and 1.02 (95% CI = 0.30, 3.50; p = 0.97) in urban areas, but this difference was not statistically significant (interaction term: p = 0.86). A test for this interaction for specific SM phenotypes could not be performed because of the small sample size with severe disease living in urban areas. The mean population density around a 30-km radius of a hospital’s location ranged between 18 per km^2^ (Macha, Zambia) and 1,562 per km^2^ (Cotonou, Benin). For children admitted to hospitals located in areas of low population density (≤300/km^2^), the OR for a delay of >24 hours compared to being admitted within the first day of symptom onset was 1.43 (95% CI = 1.11–1.84, p = 0.005) for any SM and 2.52 (95% CI = 1.75–3.64; p < 0.001) for SMA. In more densely populated areas (>300/km^2^), the equivalent ORs for SM and SMA were 1.08 (95% CI = 0.72–1.61; p = 0.72) and 4.59 (95% CI = 1.12–18.75; p = 0.03), respectively. The association between duration of illness and severity was not significantly different between the 2 population density groups (test for an interaction—SM: p = 0.32; SMA: p = 0.35).

Prior treatment with any antimalarial outside the hospital setting was much more common for severe cases (65.8%; 2,059/3,127) than uncomplicated cases (24.8%; 1,037/4,183). In children, prior treatment was associated with longer delay to admission (χ^2^ = 115.71, p < 0.001). Those who lived over 10 km from a health facility were more likely to have taken antimalarial treatment compared with those living within 10 km (OR [95% CI] = 2.65 [1.56, 4.50]; χ^2^ = 15.12, p = 0.001). Compared with receiving no prior treatment and adjusting for delay to treatment, an association of prior treatment was observed with presentation with severe disease in children (OR = 1.50, 95% CI: 1.22–1.85, p = 0.028), but this was not the case when also accounting for travelling time (OR = 1.14, 95% CI: 0.82–1.59, p = 0.45). However, an association was observed between prior antimalarial treatment and increased odds of SMA, even when accounting for both illness duration and travelling time (OR = 1.55, 95% CI: 1.03–2.33, p = 0.035), though it was unknown whether reported antimalarial use occurred before onset of severe symptoms. Prior treatment with ACT specifically was not associated with a change in the odds of any SM (OR = 0.55, 95% CI: 0.22, 1.36; p = 0.19), adjusting for treatment delay. In the subset analysis including patients who reported taking no antimalarial treatment prior to admission, similar relationships were observed between treatment delay and severity (S14 Fig). In this analysis, we estimate that 29.7% of SMA cases in children would have been averted if they received treatment within the first 24 hours of symptom onset. We further investigated the association between severity and first-line antimalarial treatment policies of each country. The studies conducted in Zambia, The Gambia (Farafenni), Yemen, Tanzania (2002), and Uganda (2003) were classified as pre-ACT. We found no significant interaction between duration of illness and classification of study as pre- or post-ACT policy (likelihood ratio test for presence of an interaction: p = 0.24 and p = 0.78 for any SM and SMA, respectively).

## Discussion

Despite substantial investment in programmes that improve rapid access to antimalarial treatment and universal acknowledgment that such access is critical, it has been difficult to quantify the impact of rapid treatment on malaria morbidity and mortality. Our analyses pooling data from 13 studies suggest that reducing delay to treatment is very strongly associated with preventing SMA, and there are also moderate associations with other SM types. Some inconsistencies observed in the association between treatment delay and severe disease in previous studies may be explained by differences in the frequency of SM types in different study populations and transmission settings, highlighting the importance of stratifying analyses by type of SM. Whilst the included studies are observational, after adjusting for potential biases, we estimate that if the association between treatment delay and SMA is causal, approximately half of SMA cases in these studies would have been prevented if all cases were treated within a day of symptom onset. This suggests the potential for substantial reduction of SM by increasing rapid access to treatment through interventions such as increasing the number of health facilities, reducing healthcare fees, or funding CHWs.

Previous studies have shown evidence of an association between mortality and treatment delay that was not observed here [53,54]. Our analysis focuses on the delay in treating UM, but mortality can be affected by many other factors after SM develops, for example, by the availability of prereferral rectal artesunate for patients who live far from a health facility [55], as well as the availability of hospital staff, facilities, antimalarial drugs, and supportive treatment procedures after presentation at the hospital. For example, blood transfusions are not available in all areas and often involve further risks of infection, particularly when appropriate tests on donors are not carried out [56,57]. Studies have suggested that long delays in receiving blood transfusions in SMA cases are associated with lower survival [58]. Despite SMA being less frequently fatal compared to other SM phenotypes in hospital-based studies, it is associated with poor long-term outcomes and higher mortality after hospital discharge [59]. Our findings highlight that improving access to treatment would be likely to reduce the need for blood transfusions and potentially reduce the high rate of postdischarge readmission and mortality [60].

Both biological reasons as well as data limitations may explain why we observed a stronger association between delay to treatment and SMA compared with other SM phenotypes such as CM and RDS. Time since onset of illness is reported by caregivers in these studies, and for some CM or RDS cases, caregivers may report the onset of the very evident severe symptoms associated with these conditions, such as coma or fast breathing, instead of uncomplicated symptoms that occur earlier. This is supported by our analysis showing that in studies which asked about onset of fever (often a first sign of UM) rather than a less specific question about onset of ‘illness’, there was more evidence of an association between treatment delay and other severe disease phenotypes than in the full pooled data. Additionally, the much higher fatality rate of CM and RDS may mean that a higher proportion of these cases die before reaching a healthcare facility compared with SMA cases in areas of low access to care. However, in the UK data, in which we may expect that few severe cases would not access healthcare, there was still a relatively strong association between treatment delay and cases developing SM.

Relevant biological differences between types of SM include the likelihood that symptoms of SMA are less specific, less easily recognised, and have a slower onset [61]. Previous studies were also unable to detect a strong association between delay from onset of fever and risk of CM [28]. Studies support the view that CM is biologically different to other SM phenotypes, with different aetiological pathways [62,63] as well as an important role of genetic variations at both the host and parasite level [64–67]. CM appears to develop faster than SMA [68], suggesting a narrower time window may exist for preventing CM by treatment that hinders detection in epidemiological studies. Such a rapid progression highlights the importance of prevention of initial infection by other interventions such as insecticide-treated nets.

Evidence from settings with very good access to antimalarial treatment are consistent with our findings. In a malaria vaccine (RTS,S) trial setting, with clinical care being part of the trial design, the ratio of the proportion of SMA versus CM amongst severe cases in 5- to 7-month–old children was lower than that observed in our data for the same age group (SMA versus CM: 5.0 for RTS,S trial and 9.0 for our pooled data set) [69]. This may suggest SMA cases are more amenable to prevention by treatment than CM cases. A large observational study conducted in Uganda showed an even greater impact when prompt treatment and regular check-ups were provided as part of the study, recording no malaria deaths or SM cases of any type [70], supporting the idea that all types of SM can be prevented by prompt treatment. The strong association between delay to treatment and presentation of SM, despite rarely presenting with SMA in the UK study, further highlights the importance of prompt treatment for all SM types.

Longer travelling times and living in rural areas, indicators of the ease of access to healthcare, were both associated with higher odds of severe disease independently of treatment delay. This is likely to indicate selection bias, in that uncomplicated cases who live further from the health facility may be less likely to seek treatment in the formal healthcare system. This bias does not, however, explain the association between delay and severe disease, which remained statistically significant and of similar size when the analysis was restricted to individuals living near the health facility (less than the median distance or travelling time). Additionally, the association between delay and severity did not differ between urban and rural settings, which further highlights this. Antimalarial treatment prior to being admitted to a formal health facility was more common in severe cases and in those living further from the hospital. This might indicate that seeking treatment outside the formal healthcare system, which may not be effective, delays appropriate treatment and increases the risk of SM. This is supported by studies in Uganda and Tanzania, which have shown that seeking care at a drug shop as a first response to illness was associated with delay in seeking care at a health facility where appropriate care was provided [24,28,71]. In addition, studies from Nigeria and Tanzania have shown evidence of progression to severe disease or SMA when not using a frontline antimalarial treatment in areas of high parasite resistance or when using suboptimal dosage [24,72]. The importance of drug resistance on severity has been highlighted by a study conducted in The Gambia during a period when chloroquine was still widely used, which found that carriage of chloroquine-resistant parasites increased the risk of SM in children [21]. It is difficult to distinguish whether seeking prior treatment delays appropriate treatment or whether more severe symptoms trigger additional treatment without delaying care. Nonetheless, the large number of cases obtaining antimalarial treatment prior to admission in this study highlights the importance of engaging all health providers (including the private sector) in malaria surveillance and case management, ensuring availability of effective and affordable antimalarials, and diagnostic testing [73].

Delay in receiving treatment was associated with a higher risk of developing SMA in low-to-moderate transmission settings (<35% slide prevalence in 2- to 10-year–olds), but not in areas with very high malaria transmission intensity (>35% *Pf*PR_2–10_). In high-transmission settings, chronic—and therefore compensated—anaemia is more common, partly because of frequent reinfection [74] as well as coinfections, malnutrition, and other risk factors linked with the poverty often present in highly endemic areas. In our study populations, even patients with UM in high-transmission settings (>35% *Pf*PR_2–10_) had on average 3.8 g/dl lower haemoglobin levels than the same group in low-to-moderate transmission settings. Therefore, progression to SMA may occur rapidly in high-transmission areas, potentially making it difficult to detect an impact of delay.

A limitation of this study is that there were relatively small numbers of adults with SM compared to children, leading to limited power to detect differences between SM phenotypes. Another main limitation is that not every study measured all markers of severity for everyone included, and therefore, conclusions may be substantially influenced by missing data. Exclusion of other conditions such as concomitant secondary bacteraemia, HIV, or other severe illnesses varied between studies. For example, asymptomatic malaria cases admitted for severe symptoms due to other conditions may be incorrectly included in the SM group. Findings may also be subject to other confounding factors that we were unable to adjust for because they were not measured in the studies included. For instance, low socioeconomic status and malnutrition may be associated with both slower treatment-seeking at a health facility and greater risk of SMA. We have addressed some of this confounding by adjusting for mother’s educational level, which did not affect the main conclusions. Duration of fever or illness was self-reported, and what constitutes as fever may vary by cultural setting [75]. True duration of illness for children with severe disease may be longer than reported because caregivers may feel reluctant to admit that care was not sought earlier. Conversely, mothers of children with SM may be more likely to remember early symptoms compared with those of children with UM [76–78].

## Conclusions

A large number of deaths from malaria in Africa occur in children under 5 and are attributable to SMA, which is estimated to constitute 47% of severe cases in high-transmission settings [79–81]. The findings of this IPD meta-analysis highlight the importance of improving access to prompt treatment in preventing SMA cases and reducing the need for potentially harmful blood transfusions. There was also evidence that prompt treatment can prevent other SM phenotypes, but this association, though statistically significant, was weaker, perhaps due to differences in underlying mechanisms of pathology between phenotypes and data limitations. CHWs have achieved increases in prompt ACT treatment coverage in many settings, but areas with the highest malaria burden have still been unable to deploy CHWs in most rural and low-access areas [79]. Our findings highlight that expansion of the provision of timely treatment is important in preventing SM and mortality.

## Supporting information

S1 Checklist PRISMA checklist of items specific to IPD meta-analyses. IPD, individual-participant data; PRISMA, Preferred Reporting Items for Systematic Reviews and Meta-Analyses
(DOCX)

S2 Checklist NOS for assessing bias in nonrandomised studies. NOS, Newcastle–Ottawa quality assessment scale
(DOCX)

S1 Text Details of search strategy (Table A) and study inclusion (Table B).
(DOCX)

S2 Text Case definitions. (DOCX)

S3 Text Institutional ethics review committees, participant consent, and study funding. (DOCX)

S1 Fig PRISMA flow diagram of the screening process and selection of eligible studies. PRISMA, Preferred Reporting Items for Systematic Reviews and Meta-Analyses
(PDF)

S2 Fig Prevalence of phenotypes amongst severe cases for each data set. Proportions were calculated for the severe cases with no missing values of a given measure. Each case may present with more than 1 phenotype. Absence of a bar may indicate that no information was collected on a phenotype (refer to Table 1 and S2 Table). For instance, the studies done in Uganda were originally designed to look at CM (and severe anaemia for 2008) only. CM, cerebral malaria.
(TIF)

S3 Fig Histograms of duration of illness prior to admission by study and severity. Histograms showing distribution of illness duration for (A) UM and (B) SM. SM, severe malaria; UM, uncomplicated malaria.
(TIF)

S4 Fig Box-and-whisker plots of duration of illness prior to admission by study and severity. Box-and-whisker plots showing median and IQR of duration of illness/fever in children <15 stratified by severity group (UM and SM) and data set are shown below (N_UM_ = 3,557, N_SM_ = 3,935). Median is shown as a thick dark line, and outliers (observations that are over 1.5 × IQR from the upper and lower quartiles) are denoted. Extreme outliers, defined as duration of illness of over 3 weeks, are omitted from these plots (0.6% of cases [20 UM and 25 SM] were omitted). SM, severe malaria; UM, uncomplicated malaria.
(TIF)

S5 Fig Illness duration by severity and study in ages 15 years or over. Box-and-whisker plots showing median and IQR of duration of illness/fever in those aged 15 and over, stratified by severity group (UM: N_UM_ = 300, SM: N_SM_ = 226). Median is shown as a thick dark line, and outliers (observations that are 1.5 × IQR from the lower or upper quartiles) are denoted. Extreme outliers, defined as duration of illness of over 3 weeks, are omitted from these plots (1.9% of cases aged 15 or over [4 UM and 6 SM] were omitted). SM, severe malaria; UM, uncomplicated malaria.
(TIF)

S6 Fig **Prevalence of phenotypes amongst severe cases in children aged between 6 months and 5 years (A), treatment delay and odds of presenting with any SM (B), SMA (C), and receiving blood transfusion (D) in children aged between 6 months and 5 years.** Proportions were calculated for severe cases with no missing values of a given measure. Each case can present with more than one phenotype. ORs (and 95% CIs) for presentation with severe disease rather than UM (A & B) with each additional reported day of delay after initial symptoms, compared with patients receiving treatment within 1 day of symptom onset (N_UM_ = 2,479, N_SM_ = 2,982, N_SMA_ = 1,519). Amongst 5 studies with information on blood transfusions during hospital admission, 29.8% (1,299/ 4,364) of children aged between 6 months to 5 years with available data had received a blood transfusion. ORs (and 95% CIs) for receiving blood transfusion was estimated for each additional day of illness duration amongst all uncomplicated and severe cases. All ORs shown were obtained from a mixed-effects logistic regression adjusted for age as a linear predictor and allowed for random study effects. Statistically significant ORs are denoted in red (dashed purple line: OR = 1). OR, odds ratio; SM, severe malaria; SMA, severe malarial anaemia; UM, uncomplicated malaria.
(TIF)

S7 Fig Treatment delay and odds of SM in adults aged 15 or over. Age-adjusted ORs (and 95% CIs) for the association between delay to treatment and presenting with (A) any severe disease, (B) prostration, (C) SMA, (D) hyperlactataemia or acidosis, (E) RDS, and (F) CM for adults aged 15 or over. Age-adjusted ORs were obtained from a mixed-effects logistic regression, with receiving treatment within 1 day of symptom onset being the reference category (dashed purple line: OR = 1). UM: N = 300; SM: N = 226; prostration: N = 103; SMA: N = 101; hyperlactataemia/acidosis: N = 40; RDS: N = 30; CM: N = 37. CM, cerebral malaria; OR, odds ratio; RDS, respiratory distress syndrome; SM, severe malaria; SMA, severe malarial anaemia; UM, uncomplicated malaria.
(TIF)

S8 Fig **Association between treatment delay and hyperlactataemia/acidosis (A), jaundice (B), prostration (C), and hyperparasitaemia (D) in children under 15 years.** Age-adjusted ORs (and 95% CIs) for the association between delay to treatment and severe disease phenotypes for children under 15. Age-adjusted ORs were obtained from a mixed-effects logistic regression, with receiving treatment within 1 day of symptom onset being the reference category (dashed purple line: OR = 1). The equivalent plots for any SM and SMA are shown in Fig 3 and for CM and RDS are shown in Fig 5. UM: N = 3,277; hyperlactataemia/acidosis: N = 950; jaundice: N = 248; prostration: N = 1,710; hyperparasitaemia: N = 206. CM, cerebral malaria; OR, odds ratio; RDS, respiratory distress syndrome; SM, severe malaria; SMA, severe malarial anaemia; UM, uncomplicated malaria.
(TIF)

S9 Fig **Duration of fever and severe malarial disease (A), SMA (B), hyperlactataemia/acidosis (C), prostration (D), and hyperparasitaemia (E).** Age-adjusted ORs (and 95% CIs) for the association between duration of fever and severe disease phenotypes for children under 15. Age-adjusted ORs were obtained from a mixed-effects logistic regression, with receiving treatment within 1 day of fever onset being the reference category (dashed purple line: OR = 1). Six categories were used instead of 8 for duration of fever because sample size was smaller than the analysis including duration of either illness or fever. The equivalent plots for CM and RDS are shown in Fig 5. The association was not explored for jaundice because of small sample size. UM: N = 492; any SM: N = 1,197; SMA: N = 528; hyperlactataemia/acidosis: N = 309, prostration: N = 707; hyperparasitaemia: N = 34. CM, cerebral malaria; OR, odds ratio; RDS, respiratory distress syndrome; SM, severe malaria; SMA, severe malarial anaemia; UM, uncomplicated malaria.
(TIF)

S10 Fig **Mortality adjusted by phenotype in severe cases (A) and the association between delay to treatment and mortality in severe cases (B), in SMA cases (C), and in CM cases (D) in children.** Adjusted ORs along with 95% CIs for mortality are shown obtained from mixed-effects logistic regression in severe cases. Panel A shows results of a model adjusting for age and presence of CM, severe anaemia, RDS, hyperlactataemia/acidosis, hypoglycaemia, and prostration in children aged under 15 years (N = 1,964). ORs for Panel A show the adjusted odds of mortality associated with each phenotype relative to other phenotypes (individuals with severe disease but without a given phenotype were considered as the reference category). Panels B–D show the association between duration of illness and odds of death amongst severe cases (B; N = 3,550), SMA cases (C; N = 1,763) and CM cases (D; N = 727). CM, cerebral malaria; OR, odds ratio; RDS, respiratory distress syndrome; SMA, severe malarial anaemia.
(TIF)

S11 Fig Travelling time and severe malarial disease. ORs (and 95% CIs) for the associations between travelling time to the health facility and presentation with any severe disease and specifically for SMA, hyperlactataemia/acidosis, RDS, CM, jaundice and hypoglycaemia for children aged <15. ORs were adjusted for age and duration of illness and were obtained from a mixed-effects logistic regression, with travelling time of under 1 hour being the reference category (dashed purple line: OR = 1). Duration of illness was fitted as a categorical variable with the following 8 categories: ≤1 day, >1 to ≤2 days, >2 to ≤3 days, >3 to ≤4 days, >4 to ≤5 days, >5 to ≤6 days, >6 to ≤7 days, >7 days. Uncomplicated: N = 2,468; any severe: N = 1,274; SMA: N = 1,274; prostration: N = 899; hyperlactataemia/acidosis: N = 541; RDS: N = 590; CM: N = 261; jaundice: N = 195; hypoglycaemia: N = 206. For the studies conducted in Farafenni (The Gambia) and the earlier Tanzanian study, travelling time was reported by either the patient or caregiver, and for Yemen, this was measured by a field assistant. For the later Tanzanian study, distance between the hospital and the individual’s district/village was estimated using AccessMod, and travelling times were then computed based on reports by local residents of how they would normally undertake the journey to the hospital (detailed criteria in Manongi and colleagues [82]). CM, cerebral malaria; OR, odds ratio; RDS, respiratory distress syndrome; SMA, severe malarial anaemia.
(TIF)

S12 Fig Distance and severe malarial disease. ORs (and 95% CIs) for the association between distance from the patient’s residence to the health facility and presenting with (A) any severe disease and specifically for (B) SMA, (C) hyperlactataemia/acidosis, (D) RDS, (E) CM, and (F) hypoglycaemia for children aged <15. ORs were adjusted for age and duration of illness and were obtained from a mixed-effects logistic regression, with distance <10 km being the reference category (dashed purple line: OR = 1). Duration of illness was fitted as a categorical variable with the following 8 categories: ≤1 day, >1 to ≤2 days, >2 to ≤3 days, >3 to ≤4 days, >4 to ≤5 days, >5 to ≤6 days, >6 to ≤7 days, >7 days. Uncomplicated: N = 479; any severe: N = 285; SMA: N = 102; hyperlactataemia/acidosis: N = 48; RDS: N = 145; CM: N = 24; hypoglycaemia: N = 27. In the study set in Benin, distance was measured between district of residence and the hospital using Google Maps. For Yemen, the distance between the patient’s residence and the hospital was measured by the visiting field assistant using car mileage. CM, cerebral malaria; OR, odds ratio; RDS, respiratory distress syndrome; SMA, severe malarial anaemia.
(TIF)

S13 Fig Accessibility and the association between duration of illness and odds of SMA compared with UM in children <15. ORs (and 95% CIs) for presentation with SMA with each additional reported day of delay after initial symptoms compared with patients receiving treatment within 1 day of symptom onset. ORs were obtained from a mixed-effects logistic regression adjusted for age as a linear predictor and allowed for random study effects. Statistically significant ORs are denoted in red (dashed purple line: OR = 1). (A) Adjusting for travelling time (N = 3,742) and (B) limiting analysis to those who live near a health facility (N = 2,026). This includes only those who live within median distance (<17.2 km) or travelling time (<35 minutes) from the hospital. OR, odds ratio; SMA, severe malarial anaemia; UM, uncomplicated malaria.
(TIF)

S14 Fig Association between SM and delay in those who reported taking no antimalarial treatment prior to admission in children <15. ORs (and 95% CIs) for presentation with (A) any SM, (B) SMA, (E) CM, and (F) RDS and requiring a blood transfusion (C) with each additional reported day of delay after initial symptoms compared with patients receiving treatment within 1 day of symptom onset. The equivalent plots for delay from fever onset are also shown for CM (G) and RDS (H). Age-adjusted change in haemoglobin (g/dl) from a mixed-effects general linear model is also shown in (D). All ORs were obtained from an age-adjusted mixed-effects logistic regression. CM, cerebral malaria; OR, odds ratio; RDS, respiratory distress syndrome; SM, severe malaria; SMA, severe malarial anaemia.
(TIF)

S1 Table Reported association between treatment delay and severity in studies not included in the pooled analysis. (DOCX)

S2 Table Frequency of severe disease phenotypes by study. Table includes study site, study period, age ranges included, and frequencies of SM phenotype groups. Percentages with a given phenotype amongst severe cases are shown in brackets and omit missing values for that phenotype. The denominator only includes those who were assessed for that phenotype. For instance, renal impairment was not systematically assessed in many of the studies. ‘NA’ entries indicate that no information was collected for that phenotype in that study. See Table 1 for other phenotypes of SM. HG, Hypoglycaemia; HL, Hyperlactataemia or Acidosis; HP, hyperparasitaemia; JN, Jaundice; PRO, Prostration; RI, Renal Impairment; SM, severe malaria.
(DOCX)

S3 Table Study design and matching of included studies. (DOCX)

S4 Table UK study of SM. Table includes data from 415 individuals aged 16 or over (median age = 35, IQR = 27–46) recruited in Northwick Park Hospital (London, UK) between April 1991 and May 2006 [36]. Table denotes frequencies and percentages with UM and SM. Percentages with a given phenotype amongst severe cases omit missing values for that phenotype. Data for RDS and prostration were not collected. Mortality during admission was estimated amongst SM cases. This study was not used in the pooled analysis but was used to compare IPD analysis findings to those from a setting with high access to care. IPD, individual-participant data; RDS, respiratory distress syndrome; SM, severe malaria; UM, uncomplicated malaria.
(DOCX)

S5 Table Frequencies of UM and SM by age group. Table includes study site, study period, age ranges included, and frequencies of UM and SM for 3 age groups (<5 years, 5 to <15 years, and 15+ years). Age was missing for 1 SM and 5 UM cases. SM, severe malaria; UM, uncomplicated malaria.
(DOCX)

S6 Table Pairwise phi coefficients between different SM phenotypes amongst severe cases. CM, cerebral malaria; HG, Hypoglycaemia; HL, Hyperlactataemia or Acidosis; HP, Hyperparasitaemia; JN, Jaundice; PRO, Prostration; RDS, respiratory distress syndrome; RI, Renal Impairment SM, severe malaria; SMA, severe malarial anaemia.
(DOCX)

S7 Table Duration of severe symptoms. Duration of severe symptoms obtained from 5 studies for 1,323 individuals. The median delay to admission in days after onset of severe symptoms and the difference between reported onset of severe symptoms and onset of uncomplicated disease are presented.
(DOCX)

S8 Table RRs for presentation with severe disease phenotypes. RRs and associated 95% CI shown were obtained using a GEE model, allowing for correlation of observations within studies. Models are shown for children (<15 years). GEE, generalised estimating equations; RR, risk ratio
(DOCX)

S9 Table **Sensitivity analysis with (A) exclusion of multiple phenotypes and (B) mother’s education as a covariate.** Age-adjusted ORs (and 95% CIs) for the association between delay to treatment and SMA, RDS, and CM in children aged under 15. Age-adjusted ORs were obtained from a mixed-effects logistic regression, with receiving treatment within 1 day of symptom onset being the reference category. For sensitivity analysis A, cases with the phenotype of interest and prostration were not excluded because of prostration being a consequence of other symptoms (for instance, large overlap between CM and prostration). For sensitivity analysis B, low mother’s education was defined as not having completed at least primary education. The mothers of 1,782 (38.0%) children did not complete primary education. Mother’s education was quantified in 4 of the studies from Yemen (n = 781), The Gambia (Farafenni; n = 447), Tanzania 2002–2003 (n = 1,309), and Tanzania 2006–2007 (n = 2,157). CM, cerebral malaria; OR, odds ratio; RDS, respiratory distress syndrome; SMA, severe malarial anaemia.
(DOCX)

S10 Table Delay to treatment and severe disease phenotypes for different malaria transmission intensity levels in children. Age-adjusted ORs (and 95% CIs) for the association between duration of illness and SMA, RDS, and CM in children under 15. Age-adjusted ORs were obtained from a mixed-effects logistic regression, with receiving treatment within 1 day of symptom onset being the reference category. Models include an interaction between transmission intensity and the effect of duration of illness on a phenotype. Transmission intensity was categorised into low (*Pf*PR_2–10_ < 10%), moderate (*Pf*PR_2–10_ of 10% to <35%), and high (*Pf*PR_2–10_ ≥ 35%). CM, cerebral malaria; OR, odds ratio; RDS, respiratory distress syndrome; SMA, severe malarial anaemia.
(DOCX)

S11 Table Mortality by severe phenotype. Case fatality in UM and SM groups. Table denotes the number of individuals with available mortality status during admission and the number (and %) of deaths amongst those. SM, severe malaria; UM, uncomplicated malaria.
(DOCX)

S1 Data (CSV)

S2 Data (XLSX)

## Abbreviations

ACT: artemisinin-based combination therapy
AHRQ: Agency for Healthcare Research and Quality
CHW: community health worker
CM: cerebral malaria
GEE: generalised estimating equations
GRUMP: Global Rural-Urban Mapping project
iCCM: integrated community case management
IPD: individual-participant data
NOS: Newcastle–Ottawa quality assessment scale
OR: odds ratio
PRISMA: Preferred Reporting Items for Systematic Reviews and Meta-Analyses
RACE: Rapid Access Expansion
RDS: respiratory distress syndrome
RR: risk ratio
SM: severe malaria
SMA: severe malarial anaemia
UM: uncomplicated malaria
