# Supplementary material for: The impact of delayed treatment of uncomplicated P. falciparum malaria on progression to severe malaria: A systematic review and a pooled multicentre individual-patient meta-analysis
Source: PLoS Med. 2020 Oct 19;17(10):e1003359. doi: 10.1371/journal.pmed.1003359 (PMC7571702; doi:10.1371/journal.pmed.1003359)
Supplement: S2 Checklist — NOS, Newcastle–Ottawa quality assessment scale (DOCX) [file pmed.1003359.s002.docx]

**S2 Checklist. Newcastle–Ottawa quality assessment scale (NOS) for assessing bias in non-randomised studies**

**Thresholds for converting the NOS rating to Agency for Healthcare Research and Quality - AHRQ - standards (good, fair, and poor):

**Good quality:** 3 or 4 stars in Selection domain AND 1 or 2 stars in Comparability domain AND 2 or 3 stars in Outcome domain

**Fair quality:** 2 stars in Selection domain AND 1 or 2 stars in Comparability domain AND 2 or 3 stars in Outcome domain

**Poor quality**: 0 or 1 star in Selection domain OR 0 stars in Comparability domain OR 0 or 1 stars in Outcome domain

The definitions for both severe cases and uncomplicated cases was well described in all studies, and comparability between the groups was high. Any variations in the quality assessment was due to differences in matching or confounding variables that a study had collected (e.g. availability of measures of accessibility such as distance and travelling time or mother’s education) and small differences in the proportion reporting symptom duration between severe and uncomplicated malaria groups

Badaut C, Guyonnet L, Milet J, Renard E, Durand R, Viwami F, et al. Immunoglobulin response to Plasmodium falciparum RESA proteins in uncomplicated and severe malaria. Malaria Journal. 2015;14.

**Cotonou, Benin (2009)**

| **No.** | **Criterion** | **Decision rule** | **Score (*)** | **Notes** |
| --- | --- | --- | --- | --- |
| **SELECTION** | | | | |
| **1** | Is the severe case definition adequate? | a) Yes, with independent validation*  b) Yes, based on self-reports  c) No description | * |  |
| **2** | Representativeness of severe cases | a) consecutive or obviously representative series of severe cases *  b) potential for selection biases or not stated |  | May be subject to selection bias, some severe cases may not reach the health facility |
| **3** | Selection of uncomplicated malaria cases | a) Uncomplicated malaria cases were selected from the same source population as the severe cases*  b) Uncomplicated malaria cases were selected from a different source population  c) no description | * |  |
| **4** | Definition of controls | a) no history of severe disease (endpoint) *  b) no description of source | * |  |
| COMPARABILITY | | | | |
| **1** | Comparability of severe cases and uncomplicated malaria cases on the basis of the design or analysis | a) study is matched for age or sex or can adjust for age *  To score an additional *:  b) study can adjust for access to health facility (travel time or distance) OR  c) study can adjust for mother’s education  (Max score= 2*) | ****** | Study not matched on age and sex but can adjust for age  Study has information on distance to the health facility but no information on mother’s education |
| EXPOSURE | | | | |
| **1** | Ascertainment of exposure | a) secure record (eg medical records records) *  b) structured interview where blind to case/control status *  c) interview not blinded to case/control status  d) written self-report or no description | ***** | Delay data is based on self-reported structured interview (blind to case/control status) so may be subject to reporting bias |
| **2** | Same method of ascertainment for severe and uncomplicated cases | a) Yes*  b) No | ***** |  |
| **3** | Non-response rate | a) Same for both groups*  b) Non-respondents described  c) Rate different and no designation | ***** | % missing duration of illness prior to admission:  Uncomplicated Malaria: 0.0%  Severe Malaria: 2.3% |
| **Total score out of 10** | | | **8/10 (Good Quality)** | |

Meerman L, Ord R, Bousema JT, van Niekerk M, Osman E, Hallett R, et al. Carriage of chloroquine-resistant parasites and delay of effective treatment increase the risk of severe malaria in Gambian children. Journal of Infectious Diseases. 2005;192(9):1651-7.

**Farafenni, The Gambia (2002)**

| **No.** | **Criterion** | **Decision rule** | **Score (*)** | **Notes** |
| --- | --- | --- | --- | --- |
| **SELECTION** | | | | |
| **1** | Is the severe case definition adequate? | a) Yes, with independent validation*  b) Yes, based on self-reports  c) No description | * |  |
| **2** | Representativeness of severe cases | a) consecutive or obviously representative series of severe cases *  b) potential for selection biases or not stated |  | May be subject to selection bias, some severe cases may not reach the health facility |
| **3** | Selection of uncomplicated malaria cases | a) Uncomplicated malaria cases were selected from the same source population as the severe cases*  b) Uncomplicated malaria cases were selected from a different source population  c) no description | * |  |
| **4** | Definition of controls | a) no history of severe disease (endpoint) *  b) no description of source | * |  |
| COMPARABILITY | | | | |
| **1** | Comparability of severe cases and uncomplicated malaria cases on the basis of the design or analysis | a) study is matched for age or sex or can adjust for age *  To score an additional *:  b) study can adjust for access to health facility (travel time or distance) OR  c) study can adjust for mother’s education  (Max score= 2*) | ****** | Study matched on age and sex and can adjust for age  Study has information on travel time to the health facility and data on mother’s education |
| EXPOSURE | | | | |
| **1** | Ascertainment of exposure | a) secure record (eg medical records records) *  b) structured interview where blind to case/control status *  c) interview not blinded to case/control status  d) written self-report or no description | ***** | Delay data is based on self-reported structured interview (blind to case/control status) so may be subject to reporting bias |
| **2** | Same method of ascertainment for severe and uncomplicated cases | a) Yes*  b) No | ***** |  |
| **3** | Non-response rate | a) Same for both groups*  b) Non-respondents described  c) Rate different and no designation | ***** | % missing duration of illness prior to admission:  Uncomplicated Malaria: 1.4%  Severe Malaria: 5.0% |
| **Total score out of 10** | | | **8/10 (Good Quality)** | |

Walther M, Jeffries D, Finney OC, Njie M, Ebonyi A, Deininger S, et al. Distinct roles for FOXP3 and FOXP3 CD4 T cells in regulating cellular immunity to uncomplicated and severe Plasmodium falciparum malaria. PLoS Pathog. 2009;5(4):e1000364.

**Serekunda, The Gambia (2007-2011)**

| **No.** | **Criterion** | **Decision rule** | **Score (*)** | **Notes** |
| --- | --- | --- | --- | --- |
| **SELECTION** | | | | |
| **1** | Is the severe case definition adequate? | a) Yes, with independent validation*  b) Yes, based on self-reports  c) No description | * |  |
| **2** | Representativeness of severe cases | a) consecutive or obviously representative series of severe cases *  b) potential for selection biases or not stated |  | May be subject to selection bias, some severe cases may not reach the health facility |
| **3** | Selection of uncomplicated malaria cases | a) Uncomplicated malaria cases were selected from the same source population as the severe cases*  b) Uncomplicated malaria cases were selected from a different source population  c) no description | * |  |
| **4** | Definition of controls | a) no history of severe disease (endpoint) *  b) no description of source | * |  |
| COMPARABILITY | | | | |
| **1** | Comparability of severe cases and uncomplicated malaria cases on the basis of the design or analysis | a) study is matched for age or sex or can adjust for age*  To score an additional *:  b) study can adjust for access to health facility (travel time or distance)  OR  c) study can adjust for mother’s education  (Max score= 2*) | ***** | Study not matched on age and sex but can adjust for age  Study has no information on travel time/distance to the health facility or mother’s education |
| EXPOSURE | | | | |
| **1** | Ascertainment of exposure | a) secure record (eg medical records records) *  b) structured interview where blind to case/control status *  c) interview not blinded to case/control status  d) written self-report or no description | ***** | Delay data is based on self-reported structured interview (blind to case/control status) so may be subject to reporting bias |
| **2** | Same method of ascertainment for severe and uncomplicated cases | a) Yes*  b) No | ***** |  |
| **3** | Non-response rate | a) Same for both groups*  b) Non-respondents described  c) Rate different and no designation | ***** | % missing duration of illness prior to admission:  Uncomplicated Malaria: 0.3%  Severe Malaria: 0.0% |
| **Total score out of 10** | | | **7/10 (Good Quality)** | |

Rees CP, Hawkesworth S, Moore SE, Dondeh BL, Unger SA. Factors Affecting Access to Healthcare: An Observational Study of Children under 5 Years of Age Presenting to a Rural Gambian Primary Healthcare Centre. Plos One. 2016;11(6).

**Keneba, The Gambia (2009-2012)**

| **No.** | **Criterion** | **Decision rule** | **Score (*)** | **Notes** |
| --- | --- | --- | --- | --- |
| **SELECTION** | | | | |
| **1** | Is the severe case definition adequate? | a) Yes, with independent validation*  b) Yes, based on self-reports  c) No description | * |  |
| **2** | Representativeness of severe cases | a) consecutive or obviously representative series of severe cases *  b) potential for selection biases or not stated |  | May be subject to selection bias, some severe cases may not reach the health facility |
| **3** | Selection of uncomplicated malaria cases | a) Uncomplicated malaria cases were selected from the same source population as the severe cases*  b) Uncomplicated malaria cases were selected from a different source population  c) no description | * |  |
| **4** | Definition of controls | a) no history of severe disease (endpoint) *  b) no description of source | * |  |
| COMPARABILITY | | | | |
| **1** | Comparability of severe cases and uncomplicated malaria cases on the basis of the design or analysis | a) study is matched for age or sex or can adjust for age*  To score an additional *:  b) study can adjust for access to health facility (travel time or distance) OR  c) study can adjust for mother’s education  (Max score= 2*) | ***** | Study not matched on age and sex but can adjust for age  Study has no information on travel time/distance to the health facility or mother’s education |
| EXPOSURE | | | | |
| **1** | Ascertainment of exposure | a) secure record (eg medical records records) *  b) structured interview where blind to case/control status *  c) interview not blinded to case/control status  d) written self-report or no description | ***** | Delay data is based on self-reported structured interview (blind to case/control status) so may be subject to reporting bias |
| **2** | Same method of ascertainment for severe and uncomplicated cases | a) Yes*  b) No | ***** |  |
| **3** | Non-response rate | a) Same for both groups*  b) Non-respondents described  c) Rate different and no designation | ***** | % missing duration of illness prior to admission:  Uncomplicated Malaria: 0.0%  Severe Malaria: 0.0% |
| **Total score out of 10** | | | **7/10 (Good Quality)** | |

Barber BE, Grigg MJ, William T, Piera KA, Boyle MJ, Yeo TW, et al. Effects of Aging on Parasite Biomass, Inflammation, Endothelial Activation, Microvascular Dysfunction and Disease Severity in Plasmodium knowlesi and Plasmodium falciparum Malaria. Journal of Infectious Diseases. 2017;215(12):1908-17.

**Sabah, Malaysia (2010- 2012)**

| **No.** | **Criterion** | **Decision rule** | **Score (*)** | **Notes** |
| --- | --- | --- | --- | --- |
| **SELECTION** | | | | |
| **1** | Is the severe case definition adequate? | a) Yes, with independent validation*  b) Yes, based on self-reports  c) No description | * |  |
| **2** | Representativeness of severe cases | a) consecutive or obviously representative series of severe cases *  b) potential for selection biases or not stated |  | May be subject to selection bias, some severe cases may not reach the health facility |
| **3** | Selection of uncomplicated malaria cases | a) Uncomplicated malaria cases were selected from the same source population as the severe cases*  b) Uncomplicated malaria cases were selected from a different source population  c) no description | * |  |
| **4** | Definition of controls | a) no history of severe disease (endpoint) *  b) no description of source | * |  |
| COMPARABILITY | | | | |
| **1** | Comparability of severe cases and uncomplicated malaria cases on the basis of the design or analysis | a) study is matched for age or sex or can adjust for age*  To score an additional *:  b) study can adjust for access to health facility (travel time or distance) OR  c) study can adjust for individual’s education  (Max score= 2*) | ***** | Study not matched on age and sex but can adjust for age  Study has no information on travel time/distance to the health facility or individual’s education |
| EXPOSURE | | | | |
| **1** | Ascertainment of exposure | a) secure record (eg medical records records) *  b) structured interview where blind to case/control status *  c) interview not blinded to case/control status  d) written self-report or no description | ***** | Delay data is based on self-reported structured interview (blind to case/control status) so may be subject to reporting bias |
| **2** | Same method of ascertainment for severe and uncomplicated cases | a) Yes*  b) No | ***** |  |
| **3** | Non-response rate | a) Same for both groups*  b) Non-respondents described  c) Rate different and no designation | ***** | % missing duration of illness prior to admission:  Uncomplicated Malaria: 0.6%  Severe Malaria: 0.0% |
| **Total score out of 10** | | | **7/10 (Good Quality)** | |

Rovira-Vallbona E, Moncunill G, Bassat Q, Aguilar R, Machevo S, Puyol L, et al. Low antibodies against Plasmodium falciparum and imbalanced pro-inflammatory cytokines are associated with severe malaria in Mozambican children: A case-control study. Malaria Journal. 2012; 11:181.

**Manhiça, Mozambique (2006)**

| **No.** | **Criterion** | **Decision rule** | **Score (*)** | **Notes** |
| --- | --- | --- | --- | --- |
| **SELECTION** | | | | |
| **1** | Is the severe case definition adequate? | a) Yes, with independent validation*  b) Yes, based on self-reports  c) No description | * |  |
| **2** | Representativeness of severe cases | a) consecutive or obviously representative series of severe cases *  b) potential for selection biases or not stated |  | May be subject to selection bias, some severe cases may not reach the health facility |
| **3** | Selection of uncomplicated malaria cases | a) Uncomplicated malaria cases were selected from the same source population as the severe cases*  b) Uncomplicated malaria cases were selected from a different source population  c) no description | * |  |
| **4** | Definition of controls | a) no history of severe disease (endpoint) *  b) no description of source | * |  |
| COMPARABILITY | | | | |
| **1** | Comparability of severe cases and uncomplicated malaria cases on the basis of the design or analysis | a) study is matched for age or sex or can adjust for age*  To score an additional *:  b) study can adjust for access to health facility (travel time or distance) OR  c) study can adjust for mother’s education  (Max score= 2*) | ***** | Study matched on age and sex and can adjust for age  Study has no information on travel time/distance to the health facility or mother’s education |
| EXPOSURE | | | | |
| **1** | Ascertainment of exposure | a) secure record (eg medical records records) *  b) structured interview where blind to case/control status *  c) interview not blinded to case/control status  d) written self-report or no description | ***** | Delay data is based on self-reported structured interview (blind to case/control status) so may be subject to reporting bias |
| **2** | Same method of ascertainment for severe and uncomplicated cases | a) Yes*  b) No | ***** |  |
| **3** | Non-response rate | a) Same for both groups*  b) Non-respondents described  c) Rate different and no designation | ***** | % missing duration of illness prior to admission:  Uncomplicated Malaria: 0.0%  Severe Malaria: 0.0% |
| **Total score out of 10** | | | **7/10 (Good Quality)** | |

Unpublished but study design identical to the previous study in Manhiça (see above)

**Manhiça, Mozambique (2014-2016)**

| **No.** | **Criterion** | **Decision rule** | **Score (*)** | **Notes** |
| --- | --- | --- | --- | --- |
| **SELECTION** | | | | |
| **1** | Is the severe case definition adequate? | a) Yes, with independent validation*  b) Yes, based on self-reports  c) No description | * |  |
| **2** | Representativeness of severe cases | a) consecutive or obviously representative series of severe cases *  b) potential for selection biases or not stated |  | May be subject to selection bias, some severe cases may not reach the health facility |
| **3** | Selection of uncomplicated malaria cases | a) Uncomplicated malaria cases were selected from the same source population as the severe cases*  b) Uncomplicated malaria cases were selected from a different source population  c) no description | * |  |
| **4** | Definition of controls | a) no history of severe disease (endpoint) *  b) no description of source | * |  |
| COMPARABILITY | | | | |
| **1** | Comparability of severe cases and uncomplicated malaria cases on the basis of the design or analysis | a) study is matched for age or sex or can adjust for age*  To score an additional *:  b) study can adjust for access to health facility (travel time or distance) OR  c) study can adjust for mother’s education  (Max score= 2*) | ***** | Study matched on age and sex and can adjust for age  Study has no information on travel time/distance to the health facility or mother’s education |
| EXPOSURE | | | | |
| **1** | Ascertainment of exposure | a) secure record (eg medical records records) *  b) structured interview where blind to case/control status *  c) interview not blinded to case/control status  d) written self-report or no description | ***** | Delay data is based on self-reported structured interview (blind to case/control status) so may be subject to reporting bias |
| **2** | Same method of ascertainment for severe and uncomplicated cases | a) Yes*  b) No | ***** |  |
| **3** | Non-response rate | a) Same for both groups*  b) Non-respondents described  c) Rate different and no designation | ***** | % missing duration of illness prior to admission:  Uncomplicated Malaria: 3.3%  Severe Malaria: 3.2% |
| **Total score out of 10** | | | **7/10 (Good Quality)** | |

Reyburn H, Mbatia R, Drakeley C, Bruce J, Carneiro I, Olomi R, et al. Association of transmission intensity and age with clinical manifestations and case fatality of severe Plasmodium falciparum malaria. Journal of the American Medical Association. 2005;293(12):1461-70.

**Kilimanjaro and Tanga, Tanzania (2002)**

| **No.** | **Criterion** | **Decision rule** | **Score (*)** | **Notes** |
| --- | --- | --- | --- | --- |
| **SELECTION** | | | | |
| **1** | Is the severe case definition adequate? | a) Yes, with independent validation*  b) Yes, based on self-reports  c) No description | * |  |
| **2** | Representativeness of severe cases | a) consecutive or obviously representative series of severe cases *  b) potential for selection biases or not stated |  | May be subject to selection bias, some severe cases may not reach the health facility |
| **3** | Selection of uncomplicated malaria cases | a) Uncomplicated malaria cases were selected from the same source population as the severe cases*  b) Uncomplicated malaria cases were selected from a different source population  c) no description | * |  |
| **4** | Definition of controls | a) no history of severe disease (endpoint) *  b) no description of source | * |  |
| COMPARABILITY | | | | |
| **1** | Comparability of severe cases and uncomplicated malaria cases on the basis of the design or analysis | a) study is matched for age or sex or can adjust for age*  To score an additional *:  b) study can adjust for access to health facility (travel time or distance) OR  c) study can adjust for mother’s education  (Max score= 2*) | ****** | Study not matched on age and sex but can adjust for age  Study has no information on travel time/distance to the health facility but has data on mother’s education |
| EXPOSURE | | | | |
| **1** | Ascertainment of exposure | a) secure record (eg medical records records) *  b) structured interview where blind to case/control status *  c) interview not blinded to case/control status  d) written self-report or no description | ***** | Delay data is based on self-reported structured interview (blind to case/control status) so may be subject to reporting bias |
| **2** | Same method of ascertainment for severe and uncomplicated cases | a) Yes*  b) No | ***** |  |
| **3** | Non-response rate | a) Same for both groups*  b) Non-respondents described  c) Rate different and no designation |  | % missing duration of illness prior to admission:  Uncomplicated Malaria: 73.5%  Severe Malaria: 1.8% |
| **Total score out of 10** | | | **7/10 (Good Quality)** | |

Nadjm Behzad, Amos Ben, Mtove George, Ostermann Jan, Chonya Semkini, Wangai Hannah et al. WHO guidelines for antimicrobial treatment in children admitted to hospital in an area of intense Plasmodium falciparum transmission: prospective study BMJ 2010; 340 :c1350

**Tanga, Tanzania (2006-2007)**

| **No.** | **Criterion** | **Decision rule** | **Score (*)** | **Notes** |
| --- | --- | --- | --- | --- |
| **SELECTION** | | | | |
| **1** | Is the severe case definition adequate? | a) Yes, with independent validation*  b) Yes, based on self-reports  c) No description | * |  |
| **2** | Representativeness of severe cases | a) consecutive or obviously representative series of severe cases *  b) potential for selection biases or not stated |  | May be subject to selection bias, some severe cases may not reach the health facility |
| **3** | Selection of uncomplicated malaria cases | a) Uncomplicated malaria cases were selected from the same source population as the severe cases*  b) Uncomplicated malaria cases were selected from a different source population  c) no description | * |  |
| **4** | Definition of controls | a) no history of severe disease (endpoint) *  b) no description of source | * |  |
| COMPARABILITY | | | | |
| **1** | Comparability of severe cases and uncomplicated malaria cases on the basis of the design or analysis | a) study is matched for age or sex or can adjust for age*  To score an additional *:  b) study can adjust for access to health facility (travel time or distance) OR  c) study can adjust for mother’s education  (Max score= 2*) | ****** | Study not matched on age and sex but can adjust for age  Study has information on travel time to the health facility and has data on mother’s education |
| EXPOSURE | | | | |
| **1** | Ascertainment of exposure | a) secure record (eg medical records records) *  b) structured interview where blind to case/control status *  c) interview not blinded to case/control status  d) written self-report or no description | ***** | Delay data is based on self-reported structured interview (blind to case/control status) so may be subject to reporting bias |
| **2** | Same method of ascertainment for severe and uncomplicated cases | a) Yes*  b) No | ***** |  |
| **3** | Non-response rate | a) Same for both groups*  b) Non-respondents described  c) Rate different and no designation | ***** | % missing duration of illness prior to admission:  Uncomplicated Malaria: 0.4%  Severe Malaria: 0.1% |
| **Total score out of 10** | | | **8/10 (Good Quality)** | |

John CC, Opika-Opoka R, Byarugaba J, Idro R, Boivin MJ. Low Levels of RANTES Are Associated with Mortality in Children with Cerebral Malaria. The Journal of infectious diseases. 2006;194(6):837-45.

**Kampala, Uganda (2003-2008)**

| **No.** | **Criterion** | **Decision rule** | **Score (*)** | **Notes** |
| --- | --- | --- | --- | --- |
| **SELECTION** | | | | |
| **1** | Is the severe case definition adequate? | a) Yes, with independent validation*  b) Yes, based on self-reports  c) No description | * |  |
| **2** | Representativeness of severe cases | a) consecutive or obviously representative series of severe cases *  b) potential for selection biases or not stated |  | May be subject to selection bias, some severe cases may not reach the health facility |
| **3** | Selection of uncomplicated malaria cases | a) Uncomplicated malaria cases were selected from the same source population as the severe cases*  b) Uncomplicated malaria cases were selected from a different source population  c) no description | * |  |
| **4** | Definition of controls | a) no history of severe disease (endpoint) *  b) no description of source | * |  |
| COMPARABILITY | | | | |
| **1** | Comparability of severe cases and uncomplicated malaria cases on the basis of the design or analysis | a) study is matched for age or sex or can adjust for age*  To score an additional *:  b) study can adjust for access to health facility (travel time or distance) OR  c) study can adjust for mother’s education  (Max score= 2*) | ***** | Study not matched on age and sex but can adjust for age  Study has no information on travel time to the health facility or information on mother’s education |
| EXPOSURE | | | | |
| **1** | Ascertainment of exposure | a) secure record (eg medical records records) *  b) structured interview where blind to case/control status *  c) interview not blinded to case/control status  d) written self-report or no description | ***** | Delay data is based on self-reported structured interview (blind to case/control status) so may be subject to reporting bias |
| **2** | Same method of ascertainment for severe and uncomplicated cases | a) Yes*  b) No | ***** |  |
| **3** | Non-response rate | a) Same for both groups*  b) Non-respondents described  c) Rate different and no designation | ***** | % missing duration of illness prior to admission:  Uncomplicated Malaria: 10.0%  Severe Malaria: 4.7% |
| **Total score out of 10** | | | **7/10 (Good Quality)** | |

Bangirana P, Opoka RO, Boivin MJ, Idro R, Hodges JS, Romero RA, et al. Severe malarial anemia is associated with long-term neurocognitive impairment. Clinical infectious diseases. 2014;59(3):336-44.

**Kampala, Uganda (2008-2013)**

| **No.** | **Criterion** | **Decision rule** | **Score (*)** | **Notes** |
| --- | --- | --- | --- | --- |
| **SELECTION** | | | | |
| **1** | Is the severe case definition adequate? | a) Yes, with independent validation*  b) Yes, based on self-reports  c) No description | * |  |
| **2** | Representativeness of severe cases | a) consecutive or obviously representative series of severe cases *  b) potential for selection biases or not stated |  | May be subject to selection bias, some severe cases may not reach the health facility |
| **3** | Selection of uncomplicated malaria cases | a) Uncomplicated malaria cases were selected from the same source population as the severe cases*  b) Uncomplicated malaria cases were selected from a different source population  c) no description | N/A | No uncomplicated control group (only community controls which were not relevant for this analysis)  N/A |
| **4** | Definition of controls | a) no history of severe disease (endpoint) *  b) no description of source | N/A | N/A (no uncomplicated malaria control group) |
| COMPARABILITY | | | | |
| **1** | Comparability of severe cases and uncomplicated malaria cases on the basis of the design or analysis | a) study is matched for age or sex or can adjust for age*  To score an additional *:  b) study can adjust for access to health facility (travel time or distance) OR  c) study can adjust for mother’s education  (Max score= 2*) | ***** | Study can adjust for age  Study has no information on travel time to the health facility or information on mother’s education |
| EXPOSURE | | | | |
| **1** | Ascertainment of exposure | a) secure record (eg medical records records) *  b) structured interview where blind to case/control status *  c) interview not blinded to case/control status  d) written self-report or no description | ***** | Delay data is based on self-reported structured interview (blind to case/control status) so may be subject to reporting bias |
| **2** | Same method of ascertainment for severe and uncomplicated cases | a) Yes*  b) No | **N/A** | N/A (no uncomplicated malaria control group) |
| **3** | Non-response rate | a) Same for both groups*  b) Non-respondents described  c) Rate different and no designation | **N/A** | % missing duration of illness prior to admission:  Severe Malaria: 0.0% |
| **Total score out of 10** | | | **N/A (no uncomplicated malaria control group)** | |

Al-Taiar A, Jaffar S, Assabri A, Al-Habori M, Azazy A, Al-Gabri A, et al. Who develops severe malaria? Impact of access to healthcare, socio-economic and environmental factors on children in Yemen: A case-control study. Tropical Medicine and International Health. 2008;13(6):762-70.

**Taiz, Yemen (2002-2004)**

| **No.** | **Criterion** | **Decision rule** | **Score (*)** | **Notes** |
| --- | --- | --- | --- | --- |
| **SELECTION** | | | | |
| **1** | Is the severe case definition adequate? | a) Yes, with independent validation*  b) Yes, based on self-reports  c) No description | * |  |
| **2** | Representativeness of severe cases | a) consecutive or obviously representative series of severe cases *  b) potential for selection biases or not stated |  | May be subject to selection bias, some severe cases may not reach the health facility |
| **3** | Selection of uncomplicated malaria cases | a) Uncomplicated malaria cases were selected from the same source population as the severe cases*  b) Uncomplicated malaria cases were selected from a different source population  c) no description | * |  |
| **4** | Definition of controls | a) no history of severe disease (endpoint) *  b) no description of source | * |  |
| COMPARABILITY | | | | |
| **1** | Comparability of severe cases and uncomplicated malaria cases on the basis of the design or analysis | a) study is matched for age or sex or can adjust for age*  To score an additional *:  b) study can adjust for access to health facility (travel time or distance) OR  c) study can adjust for mother’s education  (Max score= 2*) | ****** | Study matched on age and can adjust for age  Study has information on travel time and distance to the health facility, as well as information on mother’s education |
| EXPOSURE | | | | |
| **1** | Ascertainment of exposure | a) secure record (eg medical records records) *  b) structured interview where blind to case/control status *  c) interview not blinded to case/control status  d) written self-report or no description | ***** | Delay data is based on self-reported structured interview (blind to case/control status) so may be subject to reporting bias |
| **2** | Same method of ascertainment for severe and uncomplicated cases | a) Yes*  b) No | ***** |  |
| **3** | Non-response rate | a) Same for both groups*  b) Non-respondents described  c) Rate different and no designation | ***** | % missing duration of illness prior to admission:  Uncomplicated Malaria: 0.0%  Severe Malaria: 0.0% |
| **Total score out of 10** | | | **8/10 (Good Quality)** | |

**Macha, Southern Province, Zambia (2001-2005)**

| **No.** | **Criterion** | **Decision rule** | **Score (*)** | **Notes** |
| --- | --- | --- | --- | --- |
| **SELECTION** | | | | |
| **1** | Is the severe case definition adequate? | a) Yes, with independent validation*  b) Yes, based on self-reports  c) No description | * |  |
| **2** | Representativeness of severe cases | a) consecutive or obviously representative series of severe cases *  b) potential for selection biases or not stated |  | May be subject to selection bias, some severe cases may not reach the health facility |
| **3** | Selection of uncomplicated malaria cases | a) Uncomplicated malaria cases were selected from the same source population as the severe cases*  b) Uncomplicated malaria cases were selected from a different source population  c) no description | * |  |
| **4** | Definition of controls | a) no history of severe disease (endpoint) *  b) no description of source | * |  |
| COMPARABILITY | | | | |
| **1** | Comparability of severe cases and uncomplicated malaria cases on the basis of the design or analysis | a) study is matched for age or sex or can adjust for age*  To score an additional *:  b) study can adjust for access to health facility (travel time or distance) OR  c) study can adjust for mother’s education  (Max score= 2*) | ***** | Study not matched on age but can adjust for age  Study has no information on travel time or distance to the health facility and no information on mother’s education |
| EXPOSURE | | | | |
| **1** | Ascertainment of exposure | a) secure record (eg medical records records) *  b) structured interview where blind to case/control status *  c) interview not blinded to case/control status  d) written self-report or no description | ***** | Delay data is based on self-reported structured interview (blind to case/control status) so may be subject to reporting bias |
| **2** | Same method of ascertainment for severe and uncomplicated cases | a) Yes*  b) No | ***** |  |
| **3** | Non-response rate | a) Same for both groups*  b) Non-respondents described  c) Rate different and no designation | ***** | % missing duration of illness prior to admission:  Uncomplicated Malaria: 3.0%  Severe Malaria: 3.7% |
| **Total score out of 10** | | | **7/10 (Good Quality)** | |

Phillips A, Bassett P, Szeki S, Newman S, Pasvol G. Risk Factors for Severe Disease in Adults with Falciparum Malaria. Clinical Infectious Diseases. 2009;48(7):871-8.

**London, United Kingdom (1991-2006)**

| **No.** | **Criterion** | **Decision rule** | **Score (*)** | **Notes** |
| --- | --- | --- | --- | --- |
| **SELECTION** | | | | |
| **1** | Is the severe case definition adequate? | a) Yes, with independent validation*  b) Yes, based on self-reports  c) No description | * |  |
| **2** | Representativeness of severe cases | a) consecutive or obviously representative series of severe cases *  b) potential for selection biases or not stated |  | May be subject to selection bias, but this is less likely as this is a setting with good access to healthcare. |
| **3** | Selection of uncomplicated malaria cases | a) Uncomplicated malaria cases were selected from the same source population as the severe cases*  b) Uncomplicated malaria cases were selected from a different source population  c) no description | * |  |
| **4** | Definition of controls | a) no history of severe disease (endpoint) *  b) no description of source | * |  |
| COMPARABILITY | | | | |
| **1** | Comparability of severe cases and uncomplicated malaria cases on the basis of the design or analysis | a) study is matched for age or sex or can adjust for age*  To score an additional *:  b) study can adjust for access to health facility (travel time or distance) OR  c) study can adjust for individual’s/ mother’s education  (Max score= 2*) | ***** | Study not matched on age but can adjust for age  Study has no information on travel time or distance to the health facility and no information on individual’s/mother’s education |
| EXPOSURE | | | | |
| **1** | Ascertainment of exposure | a) secure record (eg medical records records) *  b) structured interview where blind to case/control status *  c) interview not blinded to case/control status  d) written self-report or no description | ***** | Delay data is based on self-reported structured interview (blind to case/control status) so may be subject to reporting bias |
| **2** | Same method of ascertainment for severe and uncomplicated cases | a) Yes*  b) No | ***** |  |
| **3** | Non-response rate | a) Same for both groups*  b) Non-respondents described  c) Rate different and no designation | ***** | % missing duration of illness prior to admission:  Uncomplicated Malaria: 13.0%  Severe Malaria: 10.1% |
| **Total score out of 10** | | | **7/10 (Good Quality)** | |
