# Supplementary material for: The impact of delayed treatment of uncomplicated P. falciparum malaria on progression to severe malaria: A systematic review and a pooled multicentre individual-patient meta-analysis
Source: PLoS Med. 2020 Oct 19;17(10):e1003359. doi: 10.1371/journal.pmed.1003359 (PMC7571702; doi:10.1371/journal.pmed.1003359)
Supplement: S1 Text — Details of search strategy (Table A) and study inclusion (Table B). (DOCX) [file pmed.1003359.s003.docx]

**S1 Text. Details of search strategy and study inclusion**

**Table A. Search strategy.**

| **Databases searched:**  **Ovid MEDLINE(R) ALL 1946 to November 22, 2017**  **Embase Classic+Embase 1947 to 2017 November 22** | | |
| --- | --- | --- |
| **#** | **Search** | **Fields searched** |
| 1 | (malaria* or falciparum) | All |
| 2 | (severe or cerebral) | All |
| 3 | ((duration adj3 (illness or disease)) or delay* or access* or distance or travel* or case-control or risk factor or risk factors)) | All |
| 4 | 1 AND 2 AND 3 |  |

**Table B. Inclusion and exclusion criteria.**
