# Supplementary material for: The impact of delayed treatment of uncomplicated P. falciparum malaria on progression to severe malaria: A systematic review and a pooled multicentre individual-patient meta-analysis
Source: PLoS Med. 2020 Oct 19;17(10):e1003359. doi: 10.1371/journal.pmed.1003359 (PMC7571702; doi:10.1371/journal.pmed.1003359)
Supplement: S2 Text — (DOCX) [file pmed.1003359.s004.docx]

**S2 Text. Case definitions**

**Uncomplicated malaria:** Admission to a health facility with presence of *Plasmodium falciparum* infection at the time of admission (diagnosis by either RDT, microscopy or PCR)

**Severe malaria** (based on TMIH WHO 2014 criteria)**:** Diagnosis of *Plasmodium falciparum* infection and at least one of the following symptoms, in the absence of an alternative cause:

- **Cerebral malaria:** Blantyre coma score <3 for children and Glasgow Coma Score <11 for adults. **ꝉ**
- **Severe malarial anaemia**: A haemoglobin concentration <5 g/dl or a haematocrit (PCV) of <15% in children <12 years of age (<7 g/dl and<20%, respectively, in older children and adults)
- **Acidosis or Hyperlactatemia**: plasma bicarbonate of<15 mM, base deficit of >8 meq/l or venous plasma lactate>5mM (hyperlactatemia).
- **Respiratory distress:** Due to the variations in definitions, the original definition of each study was used, which was based on increased respiratory rate (usually over 30 breaths per minute) and any signs of increased respiratory effort (e.g. nasal flaring, intercostal indrawing, subcostal recession etc.)
- **Hypoglycaemia**: Blood or plasma glucose <2.2 mM (<40 mg/dl)
- **Renal impairment**: Plasma or serum creatinine>265lM (>3 mg/dl) or blood urea>20 mM**‡**
- **Hyperparasitaemia**: *P. falciparum* parasitaemia >10% (or >500,000 parasites per μl of blood) in settings with stable endemicity and *P. falciparum* parasitaemia ≥4% (or >200,000 parasites per μl of blood) in settings with unstable endemicity (Malaysia and the UK)
- **Jaundice**: elevated plasma bilirubin (>50μM or 3mg/dl) or clinical signs of jaundice (yellowing of the skin and sclera)
- **Prostration**: the inability to sit upright in a child normally able to do so or to drink in the case of children too young to sit. All studies have used this definition of prostration. However, the age considered to be old enough to sit unsupported varied between 6 to 12 months. Data was not available to standardise age thresholds and the original definition was used.

*ꝉ For the Tanzanian studies, the original definition of cerebral malaria was used to preserve sample size. This was comprised of a BCS < 3 for young children or GCS<9 for older children and adolescents.*

*‡ The WHO TMIH [1] definition of renal impairment is different to the KDIGO criteria [2] of Acute Kidney Injury.*

1. World Health Organization. Severe Malaria. Tropical Medicine & International Health. 2014;19(s1):7-131. doi: doi:10.1111/tmi.12313_2.

2. Khwaja A. KDIGO clinical practice guidelines for acute kidney injury. Nephron Clinical Practice. 2012;120(4):c179-c84.
