# Supplementary material for: The impact of delayed treatment of uncomplicated P. falciparum malaria on progression to severe malaria: A systematic review and a pooled multicentre individual-patient meta-analysis
Source: PLoS Med. 2020 Oct 19;17(10):e1003359. doi: 10.1371/journal.pmed.1003359 (PMC7571702; doi:10.1371/journal.pmed.1003359)
Supplement: S3 Text — (DOCX) [file pmed.1003359.s005.docx]

**S3 Text. Institutional ethics review committees, participant consent and study funding**

| **Study** | **Study site** | **Years** | **Research ethics committee/institutional review board** | **Consent information** | **Funding of original study** |
| --- | --- | --- | --- | --- | --- |
| Badaut 2015 | Cotonou, Benin | Apr 2009 to Aug 2009 | The study was approved by the ethics committee of the Faculté des Sciences de la Santé of the University of Abomey-Calavi in Benin. | For each child, a written informed consent from parents or legal guardians was obtained. The study was conducted in accordance with the Declaration of Helsinki. | This work was supported by the French Agence Nationale de la Recherche under grant MIE (ANR-08-MIE-031) and CNRS. |
| Meerman 2005 | Farafenni, The Gambia | Sept 2002 to Dec 2002 | Ethical approval for the study was obtained from the Joint Gambian Government/Medical Research Council Ethics Committee. | Informed consent was obtained from all patients or their parents or guardians. Oral consent (in appropriate local language) was obtained from parents/caregivers of each child, and was documented on the information Sheet, signed and dated by the interpreter/study staff member. This procedure was approved by the Joint GG/MRC Ethics Committee in 2002. | Medical Research Council Laboratories, The Gambia; Health Protection Agency, United Kingdom (support to C.J.S.); Gates Malaria Partnership (support to R.O. and R.L.H.). |
| Walther 2009 | Serekunda, The Gambia | Aug 2007 to Jan 2011 | The study was reviewed and approved by the Joint Gambian Government/MRC Ethics Committee and the Ethics Committee of the London School of Hygiene & Tropical Medicine (London, UK). | Written informed consent was obtained from the parents or guardians. | The project was fully financed by the core budget allocated to the MRC laboratories, Fajara Malaria Programme. The funders had no role in study design, data collection and analysis, decision to publish, or preparation of the manuscript. |
| Rees 2016 | Keneba, The Gambia | Nov 2009 to Apr 2012 | Ethical approval for the study was granted by the London School of Hygiene and Tropical Medicine Ethics Board and the joint Gambia Government / MRC Unit The Gambia Ethics Committee (L2011.33). MRC Keneba has an agreement with the study population in Kiang West (KW) to undertake research studies and, prior to implementation, all studies must seek full approval by the joint Gambia Government/MRC Unit The Gambia Ethics Committee. At the community level, regular meetings between the MRC and local community are held in particular before any major study to get their support. In return the KW population receive free health care at MRC Keneba health clinic. | Informed oral and written consent was obtained from the guardian of each participating child to use data collected confidentially and identifiable by an identification number only. If guardians were illiterate the consent form would be read out by a trained field assistant and a thumbprint used to indicate full understanding and agreement once understanding had been checked. | This work was supported by Grant MC-A760-5QX00, MRC International Nutrition Group, http://www.mrc.gm. The funder had no role in study design, data collection and analysis, decision to publish, or preparation of the manuscript. |
| Barber 2017 | Sabah, Malaysia | Sept 2010 to Nov 2012 | The study was approved by the Ethics Committees of the Malaysian Ministry of Health and Menzies School of Health Research. | Informed written consent was provided by all participating adults, and by the parent or guardian of any participant aged <18 years. | This work was supported by the Australian National Health and Medical Research Council (program grants 496600 and 1037304; project grant 1045156; fellowships to B. E. B., M. J. B., T. W. Y., and N. M. A., and scholarship to M. J. G.). The Burnet Institute is supported by the Australian National Health and Medical Research Council Infrastructure for Research Institutes Support Scheme and by the Victorian State Government Operational Infrastructure Support. |
| Rovira-Vallbona 2012 | Manhiça, Mozambique | Apr 2006 to Nov 2006 | The study was approved by the National Mozambican and the Hospital Clinic of Barcelona Ethics Review Committees. | Children under five years of age attending the Manhiça District Hospital with a clinical diagnosis of P. falciparum malaria were recruited after written informed consent was given by their parents or guardians. | This work was supported by the Instituto de Salud Carlos III (grant PS09/01113; salary support FI06/00019 to ERV, CD10/00156 to GM and CP-04/00220 to AM) and the Ministerio de Ciencia e Innovación (grant SAF2008-00743; salary support RYC-2008-02631 to CD). The Centro de Investigação em Saúde de Manhiça receives core support from the Spanish Agency for International Cooperation and Development. |
| unpublished | Manhiça, Mozambique | Sept 2014 to May 2016 | The study was approved by the National Mozambican and the Hospital Clinic of Barcelona Ethics Review Committees. | Children under five years of age attending the Manhiça District Hospital with a clinical diagnosis of P. falciparum malaria were recruited after written informed consent was given by their parents or guardians. | The study was supported by La Caixa and Bill and Melinda Gates Foundations (OPP1115265) and the Instituto de Salud Carlos III [PI13/01478 cofunded by the Fondo Europeo de Desarrollo Regional (FEDER). AM was further supported by CES10/021­I3SNS and the Department of Science & Technology, Government of India (Overseas Postdoctoral Fellowship, SB/OS/PDF­043/2015­16). ISGlobal is a member of the CERCA Programme, Generalitat de Catalunya (http://cerca.cat/en/suma/). CISM is supported by the Government of Mozambique and the Spanish Agency for International Development (AECID). |
| Reyburn 2005 | Kilimanjaro and Tanga, Tanzania | Feb 2002 to Aug 2002 | Ethical approval for the study was granted by the ethical committees of the National Institute for Medical Research, Dar es Salaam, Tanzania, and the London School of Hygiene and Tropical Medicine, London, England. | Signed (or thumbprint) informed consent to participate was obtained from patients or their relatives in every case. | This study was funded by the UK Medical Research Council grant 9901439. The funders did not play any role in the study design, data collection and analysis, decision to publish, or preparation of the manuscript. |
| Nadjm 2010 | Tanga, Tanzania | Jun 2006 to May 2007 | The study was approved by the ethics committees of the National Institute for Medical Research, Tanzania, and the London School of Hygiene and Tropical Medicine. | The caregiver of each child in the study gave written informed consent. | Core funding for the study was provided by European Commission (Europaid) grant code SANTE/2004/078-607. BN was supported by grants from the Berkeley Fellowship, Sir Halley Stewart Trust, and Pfizer Pharmaceuticals. Pfizer Pharmaceuticals provided equipment and consumables for microbiology. Abbott Pharmaceuticals provided reagents for HIV testing. Netspear (www.netspear.org) funded the E-tests, which were performed at the KEMRI/Wellcome Trust Centre for Geographic Medicine (Coast), Kilifi, Kenya. None of the funders had a role in the design, analysis, or interpretation of results. |
| John 2006 | Kampala, Uganda | 2003 to 2008 | Ethics approval for the study was granted by the institutional review boards for human studies at Makerere University Faculty of Medicine, University Hospitals of Cleveland, Case Western Reserve University, and Indiana Wesleyan University | Written, informed consent was obtained from the parents or guardians of study participants. | National Institutes of Health (Fogarty Institute grant R21 TW-006794 to C.C.J.); Institute of International Education (Fulbright African Regional Research Award to M.J.B.) |
| Bangirana 2014 | Kampala, Uganda | 2008 to 2013 | Ethical approval was granted by the institutional review boards for human studies at Makerere University School of Medicine, University of Minnesota, and Michigan State University. | Written informed consent was obtained from parents or guardians of study participants. | This work was supported by the National Institute of Neurological Disorders and Stroke and the Fogarty International Center (grants R01NS055349; and D43 NS078280). |
| Al-Taiar 2008 | Taiz, Yemen | Nov 2002 to Aug 2004 | The study was approved by the ethics committees at London School of Hygiene and Tropical Medicine, United Kingdom & Ministry of Health and Population, Yemen & Faculty of Medicine and Health Sciences, Sana'a University, Yemen. | Written informed consent was obtained from the child’s parents or guardian before recruitment. | UNICEF-UNDP-World Bank-WHO Special Programme for Research and Training in Tropical Diseases (TDR), project A10491. A. Al-Taiar was supported by TDR grant A30333 and C. Whitty by the Gates Malaria Partnership, with funding from the Bill and Melinda Gates Foundation. |
| Thuma 2011 | Macha, Southern Province, Zambia | Mar 2001 to May 2005 | The study was approved by the Ethical and Research Committee of the University of Zambia (Lusaka, Zambia) and the Committee on Human Investigation of Howard University (Washington, DC). | Written informed consent was obtained for each participant from parent or guardian. | This study was supported by the National Institute of Allergy and Infectious Diseases at the National Institutes of Health (grants 1 R01 AI44857; and AI051306); the National Heart, Lung, and Blood Institute and the Office of Research on Minority Health at the National Institutes of Health (grant UH1-HL03679); and the National Institute of Research Resources, Howard University General Clinical Research Center (grant MO1-RR10284). |
| Phillips 2009 | London, United Kingdom | Apr 1991 to May 2006 | The study was approved by the Harrow Research Ethics Committee (REC 06/Q0405/24) | The original study published in Clinical Infectious Diseases was a retrospective study conducted between 1991 and 2006. It did not require written approval from the ethics committee. None of the patients were identifiable from the data and everyone received the best treatment available at the time. | Wellcome Trust (numerous grants to G.P.); Northwest London Hospitals NHS Trust (to G.P.) |
