# Supplementary material for: The impact of delayed treatment of uncomplicated P. falciparum malaria on progression to severe malaria: A systematic review and a pooled multicentre individual-patient meta-analysis
Source: PLoS Med. 2020 Oct 19;17(10):e1003359. doi: 10.1371/journal.pmed.1003359 (PMC7571702; doi:10.1371/journal.pmed.1003359)
Supplement: S1 Fig — PRISMA, Preferred Reporting Items for Systematic Reviews and Meta-Analyses (PDF) [file pmed.1003359.s006.pdf]

S1 Fig. PRISMA flow diagram of the screening process and selection of eligible studies.

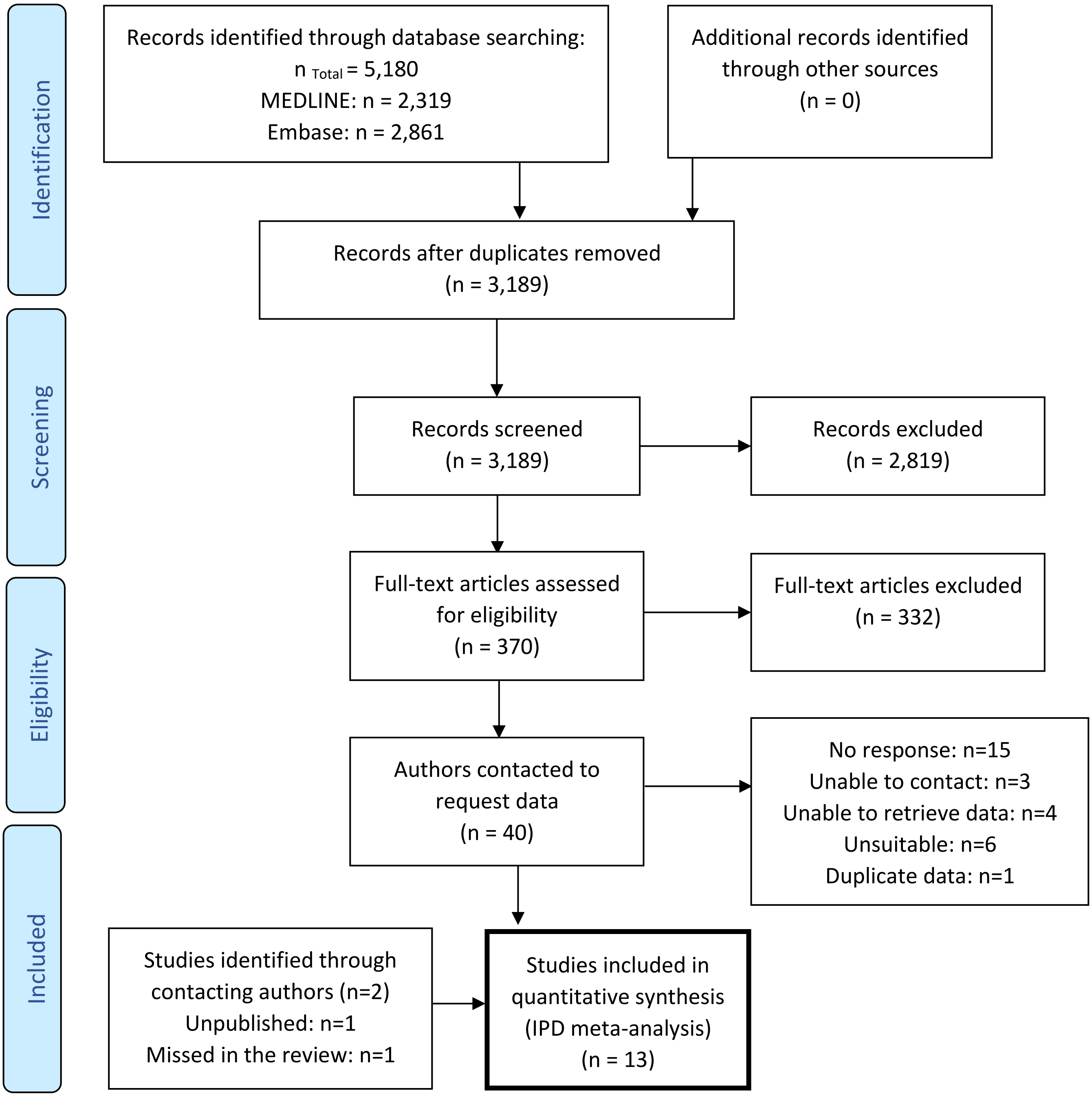

Initially, titles and abstracts were screened based on their relevance and full text screening was then performed to assess eligibility. IPD individual-level data was then sought for all 40 studies identified. All data received from the 13 studies were individual-level and none were aggregate. The number of participants for whom data were provided for the IPD analysis was 9,965. Individuals who were classified as severe in the original study but did not meet the criteria for severe disease in the IPD analysis (N=196) were excluded from the analysis. The total number of studies used in the analysis is 14, though a UK dataset on imported cases and travellers, used to compare outcomes in a setting with better access to care, was not included in the pooled IPD analysis and is omitted in the PRISMA flow diagram. All data and findings were checked with the original study's principal investigator and any issues were resolved.
