# Supplementary figures and images for: The impact of delayed treatment of uncomplicated P. falciparum malaria on progression to severe malaria: A systematic review and a pooled multicentre individual-patient meta-analysis
Source: PLoS Med. 2020 Oct 19;17(10):e1003359. doi: 10.1371/journal.pmed.1003359 (PMC7571702; doi:10.1371/journal.pmed.1003359)

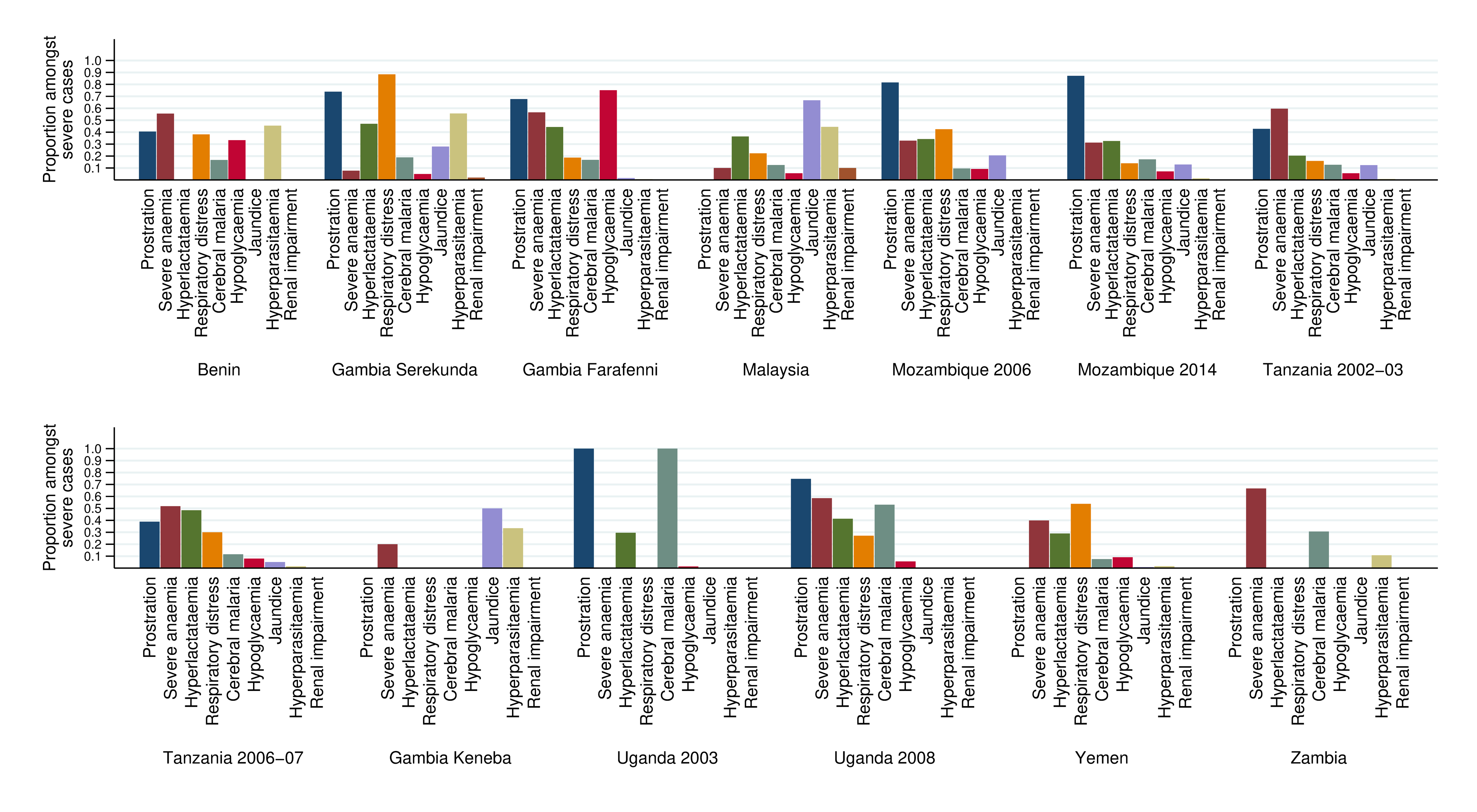

Supplement: S2 Fig — Proportions were calculated for the severe cases with no missing values of a given measure. Each case may present with more than 1 phenotype. Absence of a bar may indicate that no information was collected on a phenotype (refer to Table 1 and S2 Table). For instance, the studies done in Uganda were originally designed to look at CM (and severe anaemia for 2008) only. CM, cerebral malaria. (TIF) [file pmed.1003359.s007.tif]

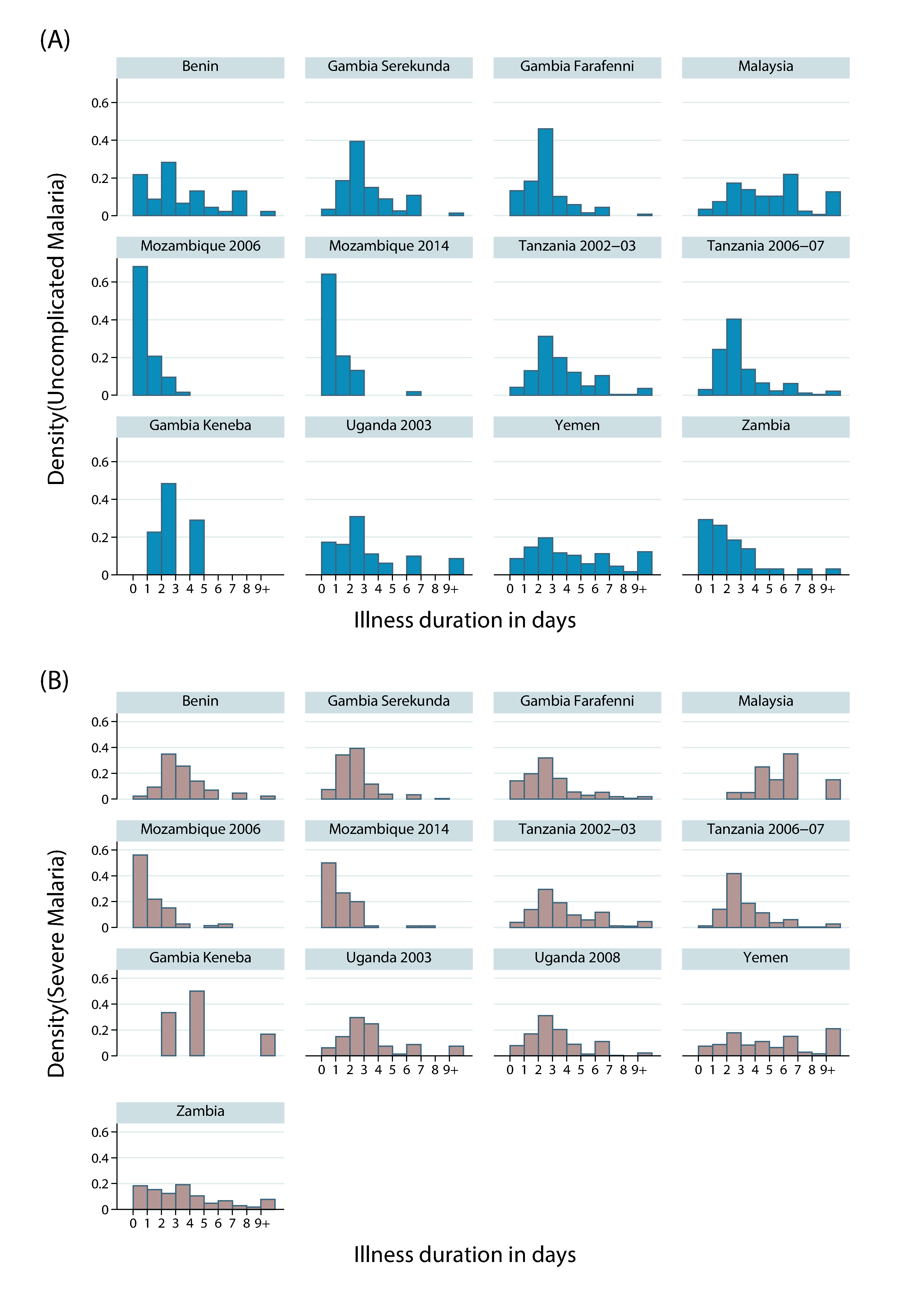

Supplement: S3 Fig — Histograms showing distribution of illness duration for (A) UM and (B) SM. SM, severe malaria; UM, uncomplicated malaria. (TIF) [file pmed.1003359.s008.tif]

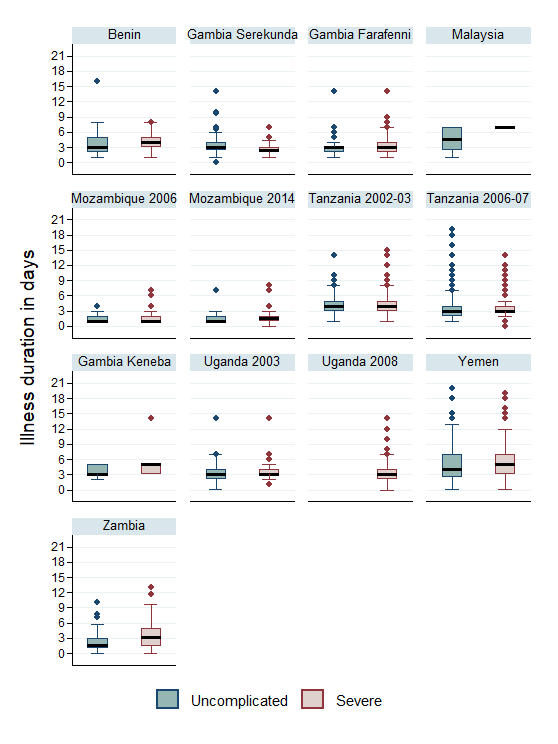

Supplement: S4 Fig — Box-and-whisker plots showing median and IQR of duration of illness/fever in children <15 stratified by severity group (UM and SM) and data set are shown below (NUM = 3,557, NSM = 3,935). Median is shown as a thick dark line, and outliers (observations that are over 1.5 × IQR from the upper and lower quartiles) are denoted. Extreme outliers, defined as duration of illness of over 3 weeks, are omitted from these plots (0.6% of cases [20 UM and 25 SM] were omitted). SM, severe malaria; UM, uncomplicated malaria. (TIF) [file pmed.1003359.s009.tif]

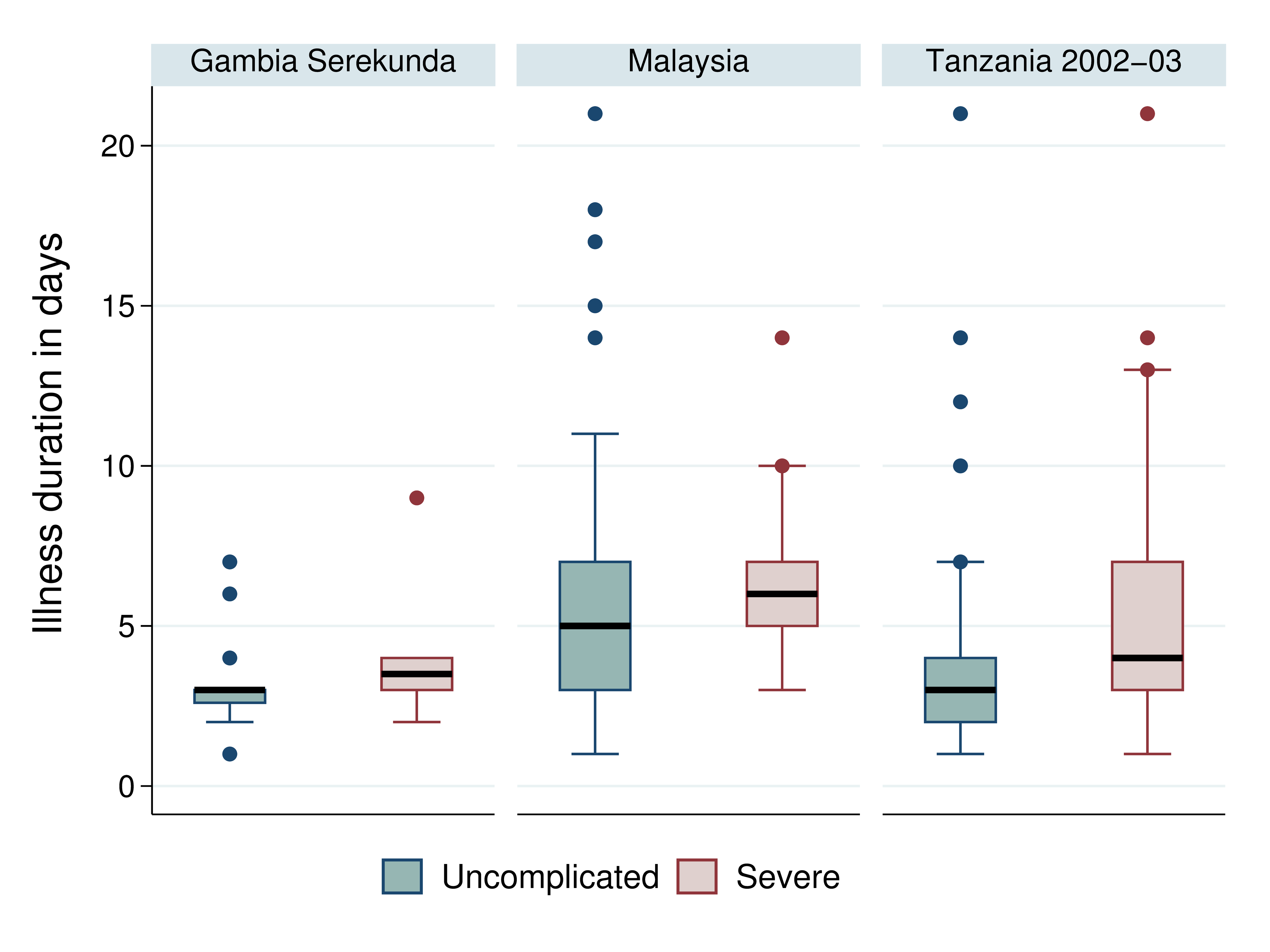

Supplement: S5 Fig — Box-and-whisker plots showing median and IQR of duration of illness/fever in those aged 15 and over, stratified by severity group (UM: NUM = 300, SM: NSM = 226). Median is shown as a thick dark line, and outliers (observations that are 1.5 × IQR from the lower or upper quartiles) are denoted. Extreme outliers, defined as duration of illness of over 3 weeks, are omitted from these plots (1.9% of cases aged 15 or over [4 UM and 6 SM] were omitted). SM, severe malaria; UM, uncomplicated malaria. (TIF) [file pmed.1003359.s010.tif]

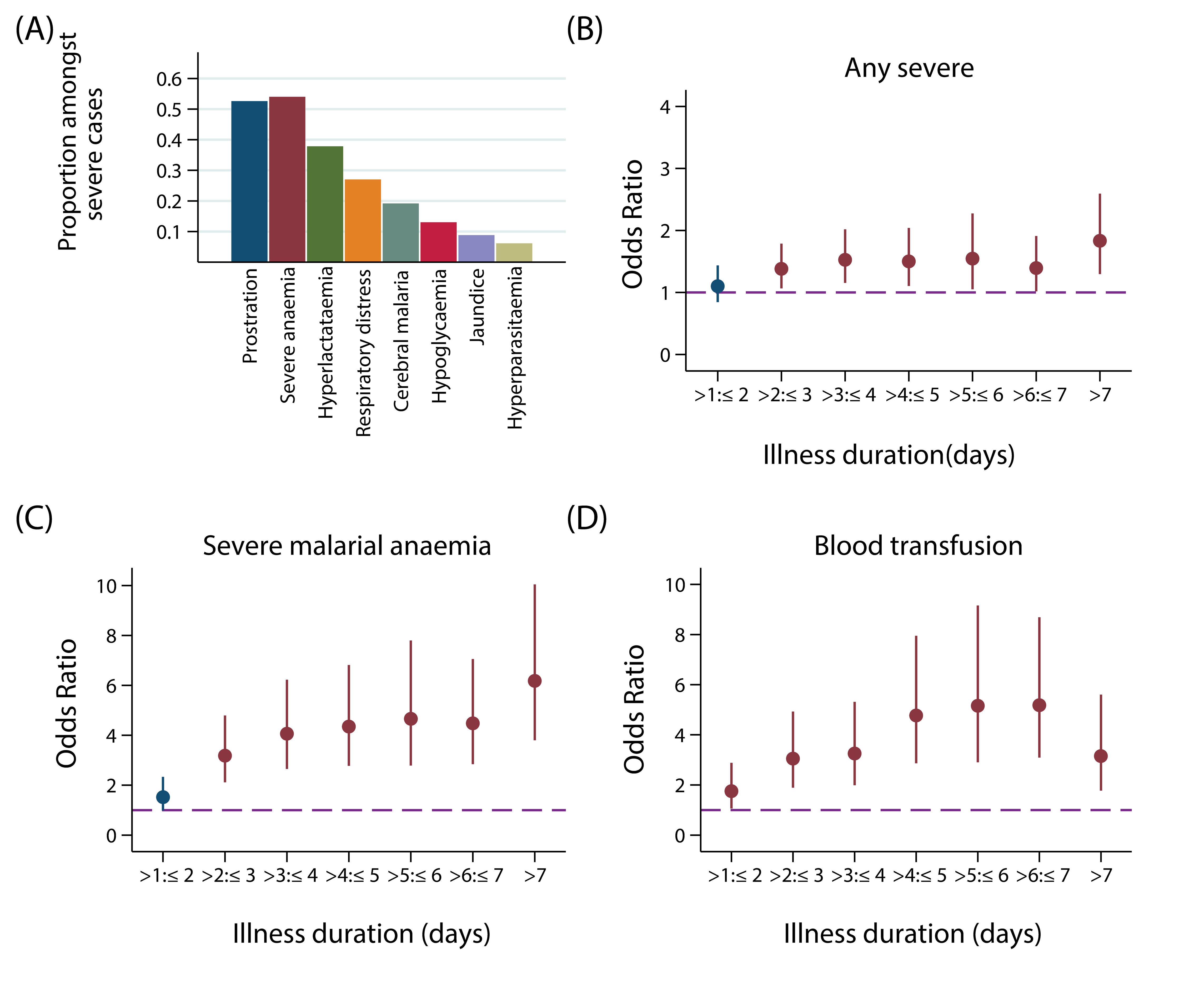

Supplement: S6 Fig — Prevalence of phenotypes amongst severe cases in children aged between 6 months and 5 years (A), treatment delay and odds of presenting with any SM (B), SMA (C), and receiving blood transfusion (D) in children aged between 6 months and 5 years. Proportions were calculated for severe cases with no missing values of a given measure. Each case can present with more than one phenotype. ORs (and 95% CIs) for presentation with severe disease rather than UM (A & B) with each additional reported day of delay after initial symptoms, compared with patients receiving treatment within 1 day of symptom onset (NUM = 2,479, NSM = 2,982, NSMA = 1,519). Amongst 5 studies with information on blood transfusions during hospital admission, 29.8% (1,299/ 4,364) of children aged between 6 months to 5 years with available data had received a blood transfusion. ORs (and 95% CIs) for receiving blood transfusion was estimated for each additional day of illness duration amongst all uncomplicated and severe cases. All ORs shown were obtained from a mixed-effects logistic regression adjusted for age as a linear predictor and allowed for random study effects. Statistically significant ORs are denoted in red (dashed purple line: OR = 1). OR, odds ratio; SM, severe malaria; SMA, severe malarial anaemia; UM, uncomplicated malaria. (TIF) [file pmed.1003359.s011.tif]

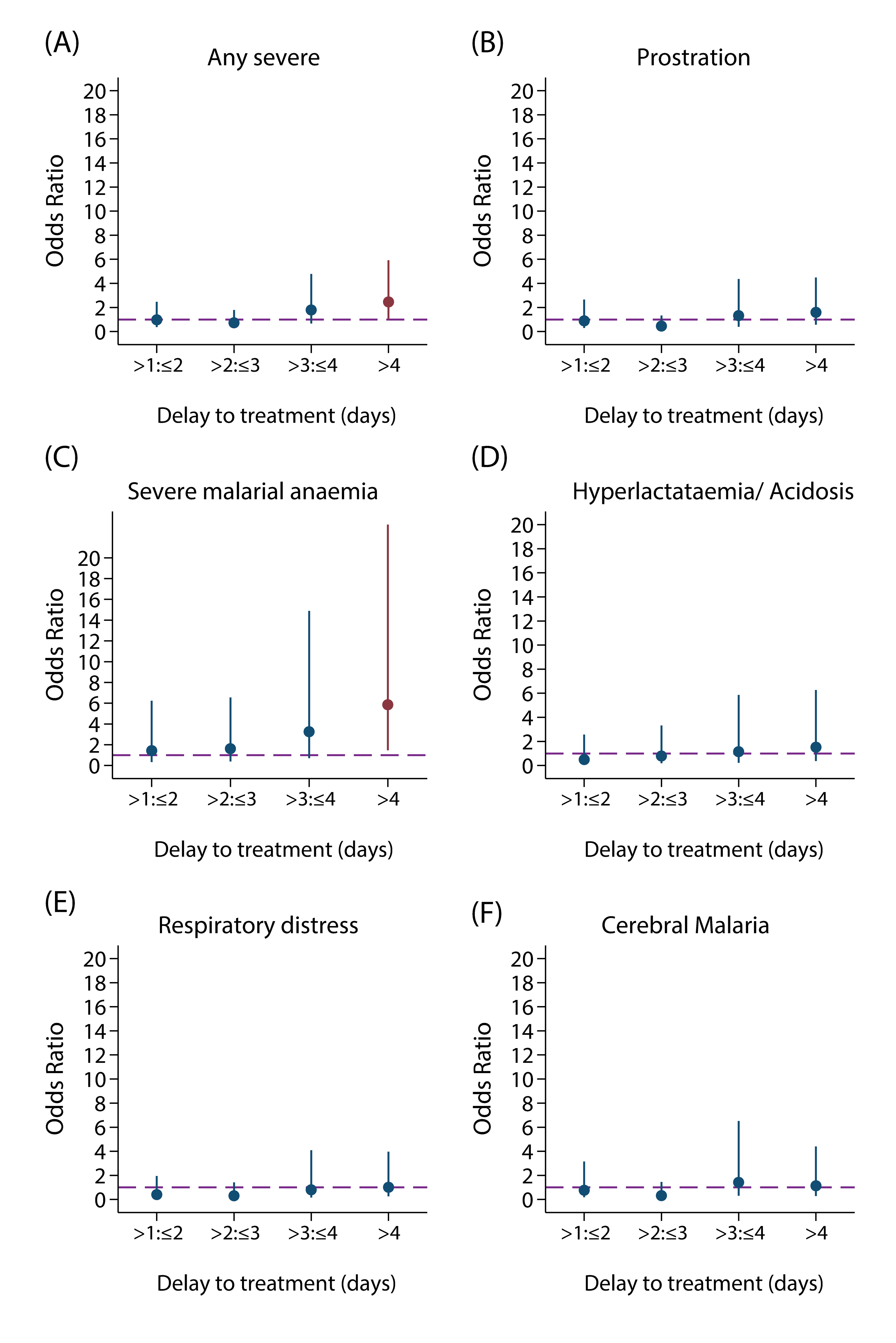

Supplement: S7 Fig — Age-adjusted ORs (and 95% CIs) for the association between delay to treatment and presenting with (A) any severe disease, (B) prostration, (C) SMA, (D) hyperlactataemia or acidosis, (E) RDS, and (F) CM for adults aged 15 or over. Age-adjusted ORs were obtained from a mixed-effects logistic regression, with receiving treatment within 1 day of symptom onset being the reference category (dashed purple line: OR = 1). UM: N = 300; SM: N = 226; prostration: N = 103; SMA: N = 101; hyperlactataemia/acidosis: N = 40; RDS: N = 30; CM: N = 37. CM, cerebral malaria; OR, odds ratio; RDS, respiratory distress syndrome; SM, severe malaria; SMA, severe malarial anaemia; UM, uncomplicated malaria. (TIF) [file pmed.1003359.s012.tif]

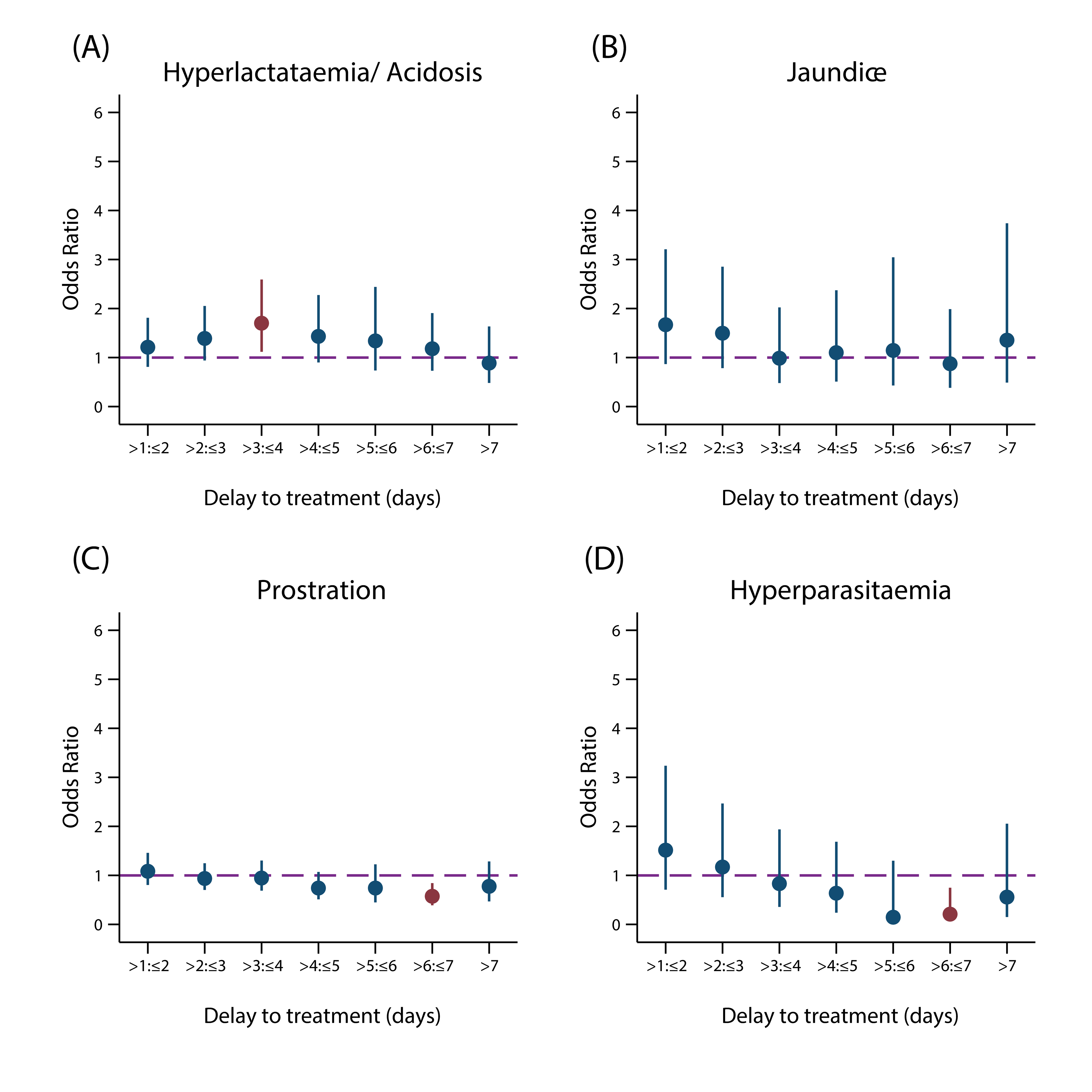

Supplement: S8 Fig — Association between treatment delay and hyperlactataemia/acidosis (A), jaundice (B), prostration (C), and hyperparasitaemia (D) in children under 15 years. Age-adjusted ORs (and 95% CIs) for the association between delay to treatment and severe disease phenotypes for children under 15. Age-adjusted ORs were obtained from a mixed-effects logistic regression, with receiving treatment within 1 day of symptom onset being the reference category (dashed purple line: OR = 1). The equivalent plots for any SM and SMA are shown in Fig 3 and for CM and RDS are shown in Fig 5. UM: N = 3,277; hyperlactataemia/acidosis: N = 950; jaundice: N = 248; prostration: N = 1,710; hyperparasitaemia: N = 206. CM, cerebral malaria; OR, odds ratio; RDS, respiratory distress syndrome; SM, severe malaria; SMA, severe malarial anaemia; UM, uncomplicated malaria. (TIF) [file pmed.1003359.s013.tif]

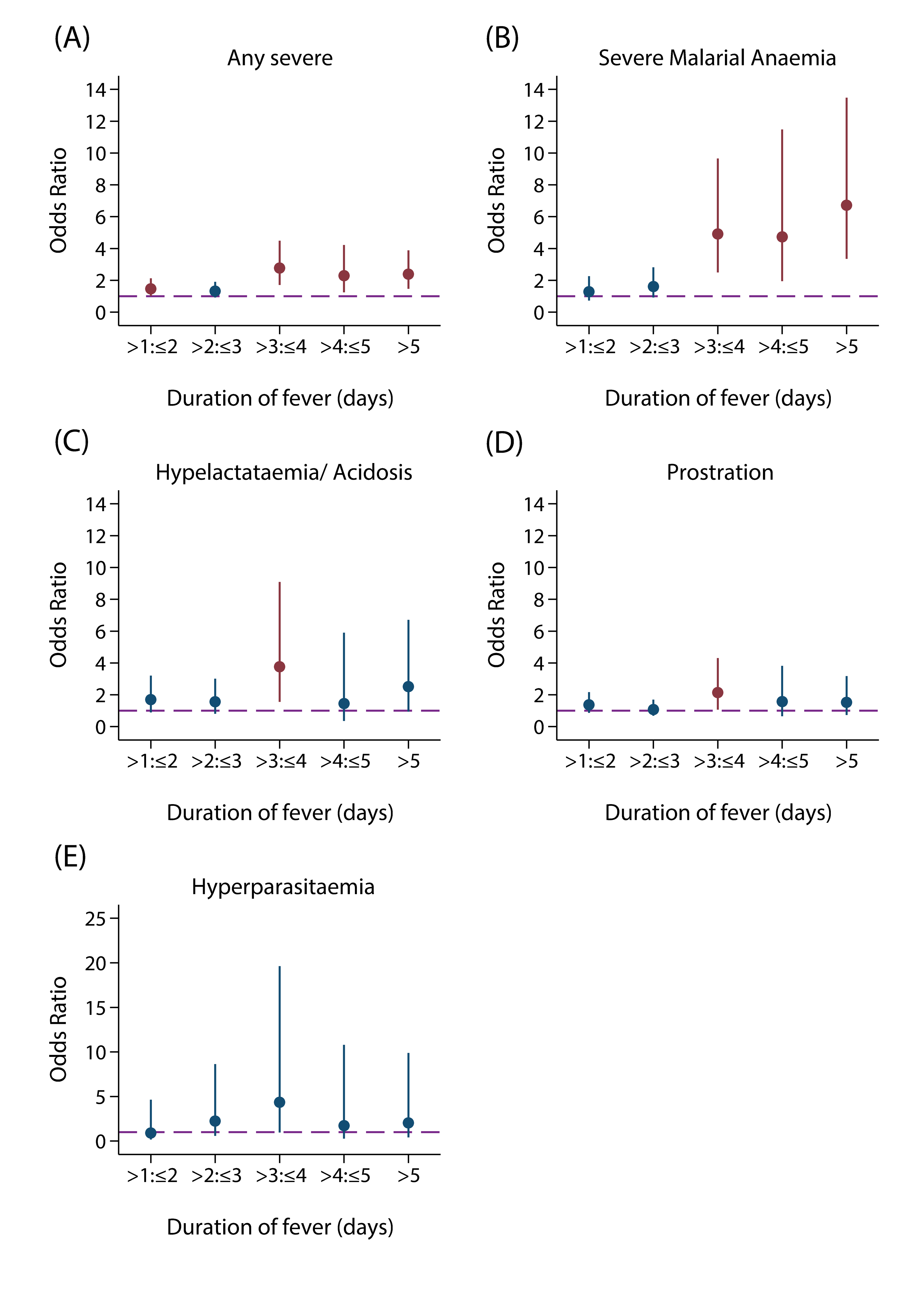

Supplement: S9 Fig — Duration of fever and severe malarial disease (A), SMA (B), hyperlactataemia/acidosis (C), prostration (D), and hyperparasitaemia (E). Age-adjusted ORs (and 95% CIs) for the association between duration of fever and severe disease phenotypes for children under 15. Age-adjusted ORs were obtained from a mixed-effects logistic regression, with receiving treatment within 1 day of fever onset being the reference category (dashed purple line: OR = 1). Six categories were used instead of 8 for duration of fever because sample size was smaller than the analysis including duration of either illness or fever. The equivalent plots for CM and RDS are shown in Fig 5. The association was not explored for jaundice because of small sample size. UM: N = 492; any SM: N = 1,197; SMA: N = 528; hyperlactataemia/acidosis: N = 309, prostration: N = 707; hyperparasitaemia: N = 34. CM, cerebral malaria; OR, odds ratio; RDS, respiratory distress syndrome; SM, severe malaria; SMA, severe malarial anaemia; UM, uncomplicated malaria. (TIF) [file pmed.1003359.s014.tif]

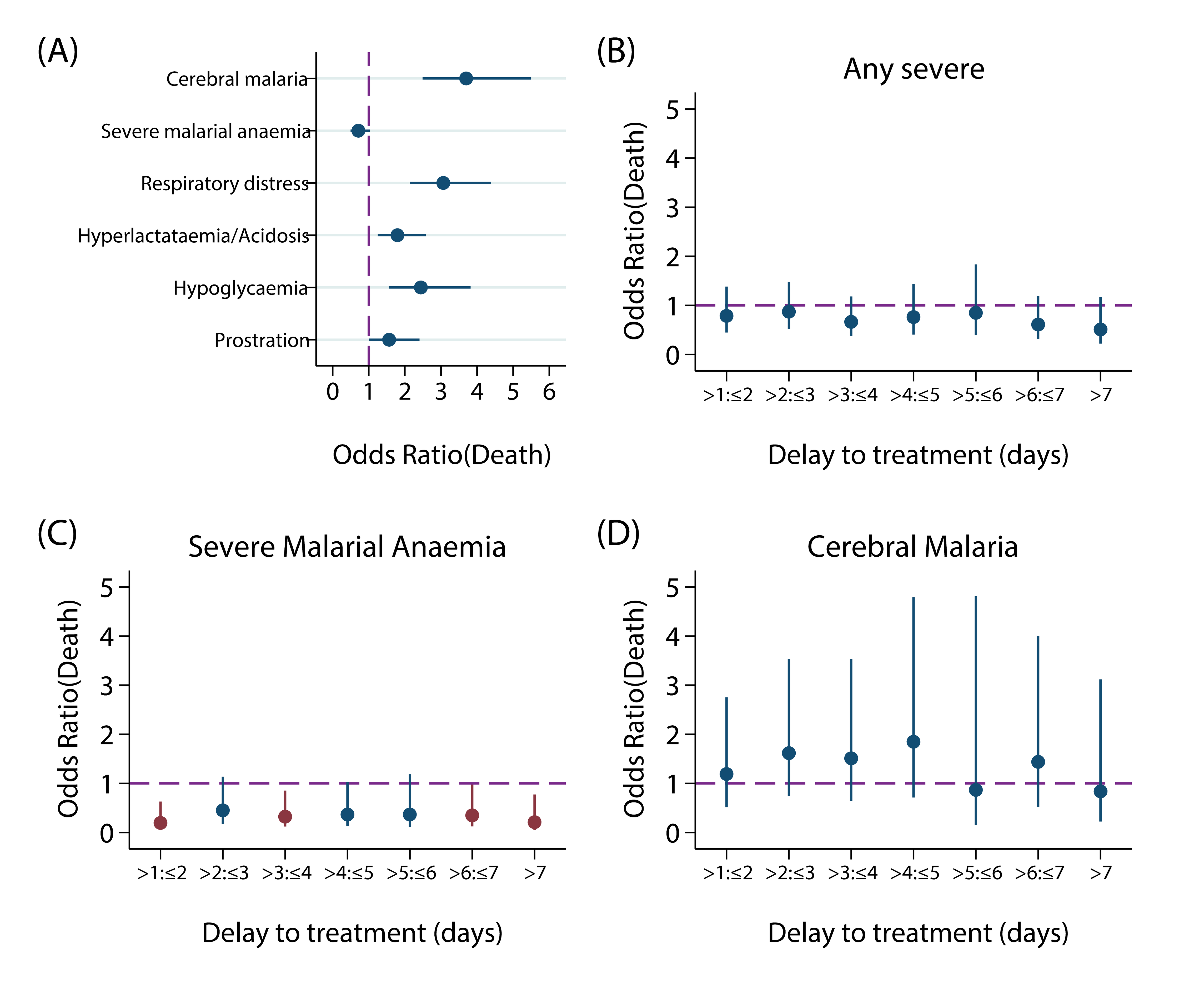

Supplement: S10 Fig — Mortality adjusted by phenotype in severe cases (A) and the association between delay to treatment and mortality in severe cases (B), in SMA cases (C), and in CM cases (D) in children. Adjusted ORs along with 95% CIs for mortality are shown obtained from mixed-effects logistic regression in severe cases. Panel A shows results of a model adjusting for age and presence of CM, severe anaemia, RDS, hyperlactataemia/acidosis, hypoglycaemia, and prostration in children aged under 15 years (N = 1,964). ORs for Panel A show the adjusted odds of mortality associated with each phenotype relative to other phenotypes (individuals with severe disease but without a given phenotype were considered as the reference category). Panels B–D show the association between duration of illness and odds of death amongst severe cases (B; N = 3,550), SMA cases (C; N = 1,763) and CM cases (D; N = 727). CM, cerebral malaria; OR, odds ratio; RDS, respiratory distress syndrome; SMA, severe malarial anaemia. (TIF) [file pmed.1003359.s015.tif]

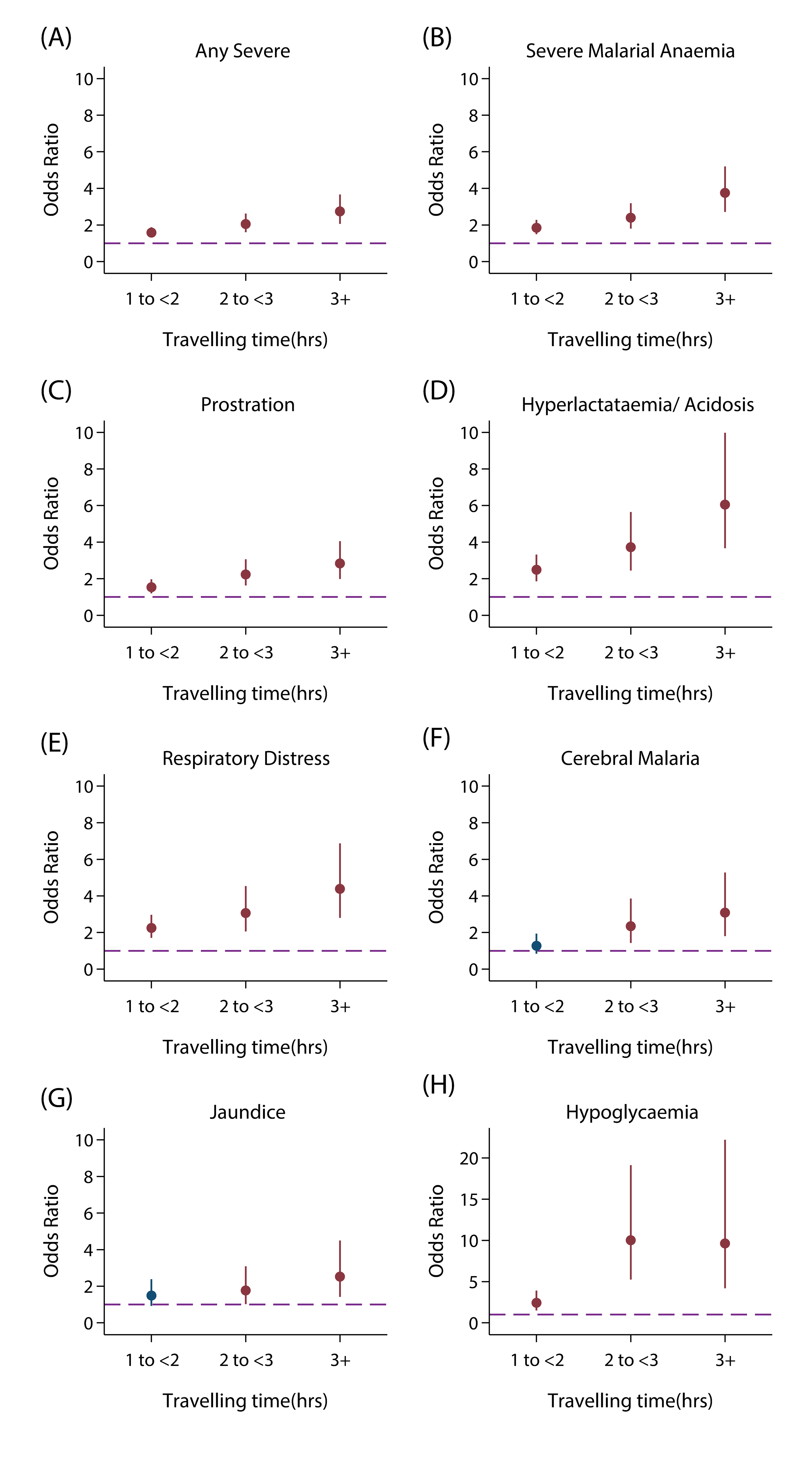

Supplement: S11 Fig — ORs (and 95% CIs) for the associations between travelling time to the health facility and presentation with any severe disease and specifically for SMA, hyperlactataemia/acidosis, RDS, CM, jaundice and hypoglycaemia for children aged <15. ORs were adjusted for age and duration of illness and were obtained from a mixed-effects logistic regression, with travelling time of under 1 hour being the reference category (dashed purple line: OR = 1). Duration of illness was fitted as a categorical variable with the following 8 categories: ≤1 day, >1 to ≤2 days, >2 to ≤3 days, >3 to ≤4 days, >4 to ≤5 days, >5 to ≤6 days, >6 to ≤7 days, >7 days. Uncomplicated: N = 2,468; any severe: N = 1,274; SMA: N = 1,274; prostration: N = 899; hyperlactataemia/acidosis: N = 541; RDS: N = 590; CM: N = 261; jaundice: N = 195; hypoglycaemia: N = 206. For the studies conducted in Farafenni (The Gambia) and the earlier Tanzanian study, travelling time was reported by either the patient or caregiver, and for Yemen, this was measured by a field assistant. For the later Tanzanian study, distance between the hospital and the individual’s district/village was estimated using AccessMod, and travelling times were then computed based on reports by local residents of how they would normally undertake the journey to the hospital (detailed criteria in Manongi and colleagues [82]). CM, cerebral malaria; OR, odds ratio; RDS, respiratory distress syndrome; SMA, severe malarial anaemia. (TIF) [file pmed.1003359.s016.tif]

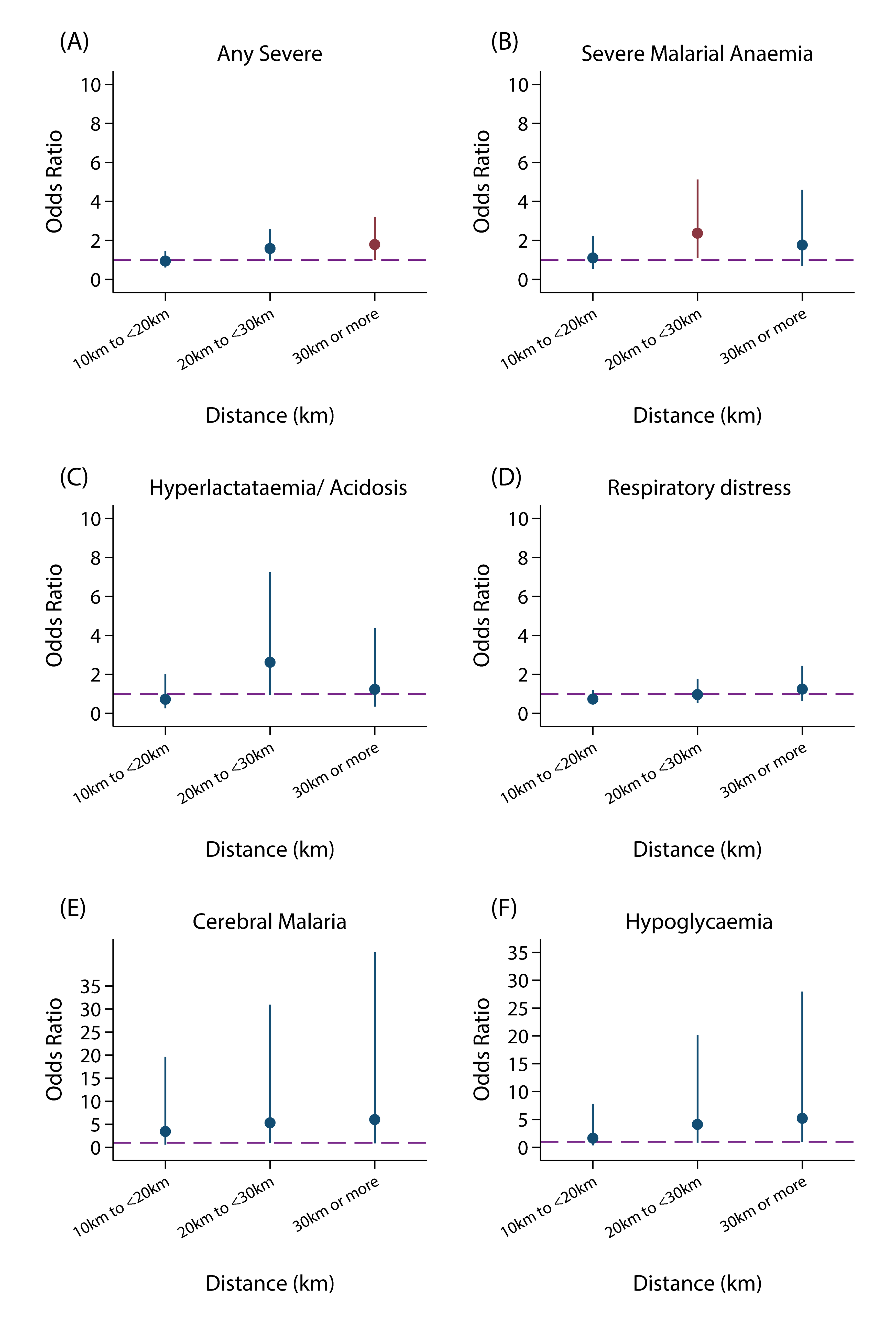

Supplement: S12 Fig — ORs (and 95% CIs) for the association between distance from the patient’s residence to the health facility and presenting with (A) any severe disease and specifically for (B) SMA, (C) hyperlactataemia/acidosis, (D) RDS, (E) CM, and (F) hypoglycaemia for children aged <15. ORs were adjusted for age and duration of illness and were obtained from a mixed-effects logistic regression, with distance <10 km being the reference category (dashed purple line: OR = 1). Duration of illness was fitted as a categorical variable with the following 8 categories: ≤1 day, >1 to ≤2 days, >2 to ≤3 days, >3 to ≤4 days, >4 to ≤5 days, >5 to ≤6 days, >6 to ≤7 days, >7 days. Uncomplicated: N = 479; any severe: N = 285; SMA: N = 102; hyperlactataemia/acidosis: N = 48; RDS: N = 145; CM: N = 24; hypoglycaemia: N = 27. In the study set in Benin, distance was measured between district of residence and the hospital using Google Maps. For Yemen, the distance between the patient’s residence and the hospital was measured by the visiting field assistant using car mileage. CM, cerebral malaria; OR, odds ratio; RDS, respiratory distress syndrome; SMA, severe malarial anaemia. (TIF) [file pmed.1003359.s017.tif]

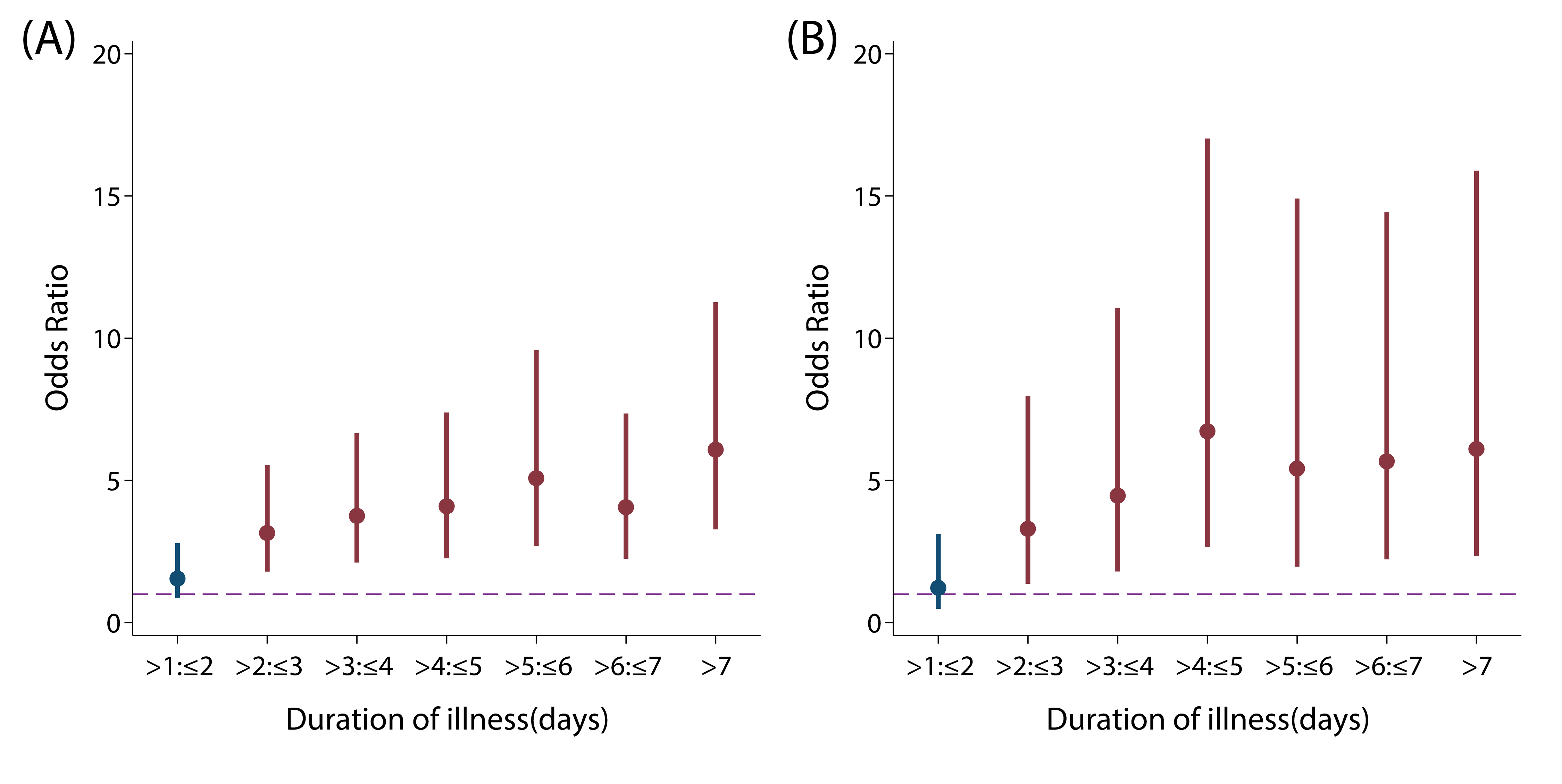

Supplement: S13 Fig — ORs (and 95% CIs) for presentation with SMA with each additional reported day of delay after initial symptoms compared with patients receiving treatment within 1 day of symptom onset. ORs were obtained from a mixed-effects logistic regression adjusted for age as a linear predictor and allowed for random study effects. Statistically significant ORs are denoted in red (dashed purple line: OR = 1). (A) Adjusting for travelling time (N = 3,742) and (B) limiting analysis to those who live near a health facility (N = 2,026). This includes only those who live within median distance (<17.2 km) or travelling time (<35 minutes) from the hospital. OR, odds ratio; SMA, severe malarial anaemia; UM, uncomplicated malaria. (TIF) [file pmed.1003359.s018.tif]

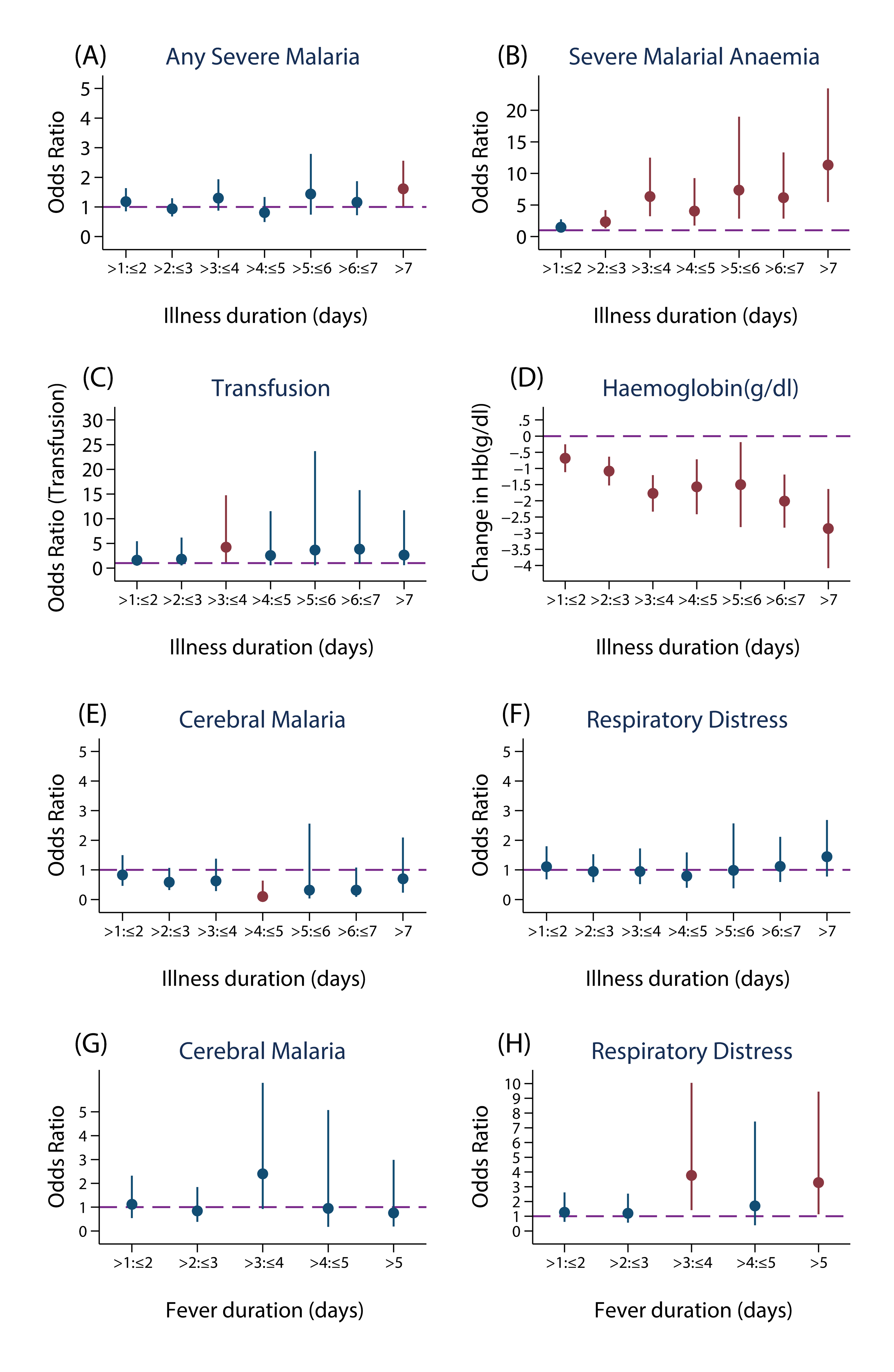

Supplement: S14 Fig — ORs (and 95% CIs) for presentation with (A) any SM, (B) SMA, (E) CM, and (F) RDS and requiring a blood transfusion (C) with each additional reported day of delay after initial symptoms compared with patients receiving treatment within 1 day of symptom onset. The equivalent plots for delay from fever onset are also shown for CM (G) and RDS (H). Age-adjusted change in haemoglobin (g/dl) from a mixed-effects general linear model is also shown in (D). All ORs were obtained from an age-adjusted mixed-effects logistic regression. CM, cerebral malaria; OR, odds ratio; RDS, respiratory distress syndrome; SM, severe malaria; SMA, severe malarial anaemia. (TIF) [file pmed.1003359.s019.tif]
