# Supplementary material for: The impact of delayed treatment of uncomplicated P. falciparum malaria on progression to severe malaria: A systematic review and a pooled multicentre individual-patient meta-analysis
Source: PLoS Med. 2020 Oct 19;17(10):e1003359. doi: 10.1371/journal.pmed.1003359 (PMC7571702; doi:10.1371/journal.pmed.1003359)
Supplement: S1 Table — (DOCX) [file pmed.1003359.s020.docx]

**S1 Table- Reported association between treatment delay and severity in studies not included in the pooled analysis.**

| **Study** | **Setting** | **Period** | **Ages** | **Sample size** | **Delay OR (95%CI)** | **Comparison** |
| --- | --- | --- | --- | --- | --- | --- |
| Mpimbaza 2017[1] | Jinja, Uganda | Mar 2015 to Mar 2016 | 4 months to 10 years | 325 SM 325 UM | Severe Malaria: aOR= 3.77 (2.35-6.03)  Severe Malarial Anaemia: aOR=15.6 (3.02,80.6) Impaired consciousness aOR= 1.13(0.30, 4.28)  *aOR adjusted for age, gametocytaemia, health care seeking, caregiver's and home characteristics* | ≥24 hours vs. <24 hours ≥24 hours vs. <24 hours ≥24 hours vs. <24 hours |
| Kahabuka 2012[2] | Tanga, Tanzania | Jul 2009 to Jan 2010 | 1 month to 5 years | 293 SM  190 UM | Severe Malaria: OR=3.0(1.9-4.8), aOR=2.9(1.8-4.7)  Severe Malaria: OR=2.4(1.5-4.0), aOR= 2.1(1.3-3.6)  *aOR: adjusted for age, sex, caregiver's education and SES* | 3-4 days vs. 1-2 days ≥5 days vs. 1-2 days |
| Zoungrana 2014[3] | Koudougou, Burkina Faso | Jul 2012 to Sept 2012 | under 5 | 201 SM 309 UM | Severe Malaria: aOR=4.53 (1.76-11.65) *aOR: adjusted for parent characteristics  (e.g. education and SES)* | ≥4 days vs. 4 days |
| Ossou-Nguiet 2013[4] | Brazzaville, Congo | Jul 2011 to Dec 2011 | 4 months to 14 years | 230 CM 123 UM | CM: OR=5.11(1.71-15.29)  CM: OR=13.69(2.57-31.90) | 4-7 days vs.<4 days  ≥7 days vs. <7 days |
| Mutsigiri-Murewanhema 2017[5] | Mutasa and Nyanga, Zimbabwe | Sep 2014 to May 2015 | under 10 | 52 SM 104 UM | Severe Malaria: OR**ᶧ**=14.30 (4.57, 45.36)  Severe Malaria: OR**‡**=5.59 (2.67, 11.68)  Severe Malaria: aOR**ᶧ**=9.03(2.21, 36.93)  Severe Malaria: aOR**‡**=3.82(1.44,10.12)  ᶧ***delay to seeking medical care*** **‡*delay to antimalarial treatment*** *aOR= adjusted for distance, comorbidities, ITN ownership* | >2 days vs. ≤2 days >24hrs vs. ≤24 hrs >2 days vs. ≤2 days >24hrs vs. ≤24 hrs |

1. Mpimbaza A, Ndeezi G, Katahoire A, Rosenthal PJ, Karamagi C. Demographic, Socioeconomic, and Geographic Factors Leading to Severe Malaria and Delayed Care Seeking in Ugandan Children: A Case-Control Study. The American journal of tropical medicine and hygiene. 2017;97(5):1513-23. doi: 10.4269/ajtmh.17-0056.

2. Kahabuka C, Kvåle G, Hinderaker SG. Factors associated with severe disease from malaria, pneumonia and diarrhea among children in rural Tanzania – A hospital-based cross-sectional study. BMC Infectious Diseases. 2012;12:219-. doi: 10.1186/1471-2334-12-219.

3. Zoungrana A, Chou YJ, Pu C. Socioeconomic and environment determinants as predictors of severe malaria in children under 5 years of age admitted in two hospitals in Koudougou district, Burkina Faso: a cross sectional study. Acta tropica. 2014;139:109-14. doi: 10.1016/j.actatropica.2014.07.011.

4. Ossou-Nguiet PM, Okoko AR, Ekouya Bowassa G, Oko AP, Mabiala-Babela JR, Ndjobo Mamadoud IC, et al. Determinants of cerebral malaria in Congolese children. Revue neurologique. 2013;169(6-7):510-4. doi: 10.1016/j.neurol.2012.11.003.

5. Mutsigiri-Murewanhema F, Mafaune PT, Shambira G, Juru T, Bangure D, Mungati M, et al. Factors associated with severe malaria among children below ten years in Mutasa and Nyanga districts, Zimbabwe, 2014-2015. Pan Afr Med J. 2017;27:23-. doi: 10.11604/pamj.2017.27.23.10957.

References:
