# Supplementary material for: The impact of delayed treatment of uncomplicated P. falciparum malaria on progression to severe malaria: A systematic review and a pooled multicentre individual-patient meta-analysis
Source: PLoS Med. 2020 Oct 19;17(10):e1003359. doi: 10.1371/journal.pmed.1003359 (PMC7571702; doi:10.1371/journal.pmed.1003359)
Supplement: S2 Table — Table includes study site, study period, age ranges included, and frequencies of SM phenotype groups. Percentages with a given phenotype amongst severe cases are shown in brackets and omit missing values for that phenotype. The denominator only includes those who were assessed for that phenotype. For instance, renal impairment was not systematically assessed in many of the studies. ‘NA’ entries indicate that no information was collected for that phenotype in that study. See Table 1 for other phenotypes of SM. HG, Hypoglycaemia; HL, Hyperlactataemia or Acidosis; HP, hyperparasitaemia; JN, Jaundice; PRO, Prostration; RI, Renal Impairment; SM, severe malaria. (DOCX) [file pmed.1003359.s021.docx]

**S2 Table. Frequency of severe disease phenotypes by study.** Table includes study site, study period, age ranges included, and frequencies of severe malaria phenotypes groups. Percentages with a given phenotype among severe cases are shown in brackets and omit missing values for that phenotype. The denominator only includes those who were assessed for that phenotype. For instance, renal impairment was not systematically assessed in many of the studies. “NA” entries indicate that no information was collected for that phenotype in that study. Definitions: PRO= Prostration; HL= Hyperlactatemia or Acidosis; HG= Hypoglycaemia; JN= Jaundice; HP= Hyperparasitaemia; RI= Renal Impairment. See *Table 1* for other phenotypes of severe malaria.

| **Study site** | **Years** | **Age range** | **PRO (%)** | **HL (%)** | **HG (%)** | **JN (%)** | **HP (%)** | **RI (%)** |
| --- | --- | --- | --- | --- | --- | --- | --- | --- |
| Cotonou, Benin | Apr 2009 to Aug 2009 | 4 months to 14 years | 17 (40.5) | NA | 4 (33.3) | NA | 20 (45.5) | NA |
| Farafenni, The Gambia | Sept 2002 to Dec 2002 | 1 months to 10 years | 216 (67.7) | 59 (44.4) | 154 (75.1) | 5 (1.6) | 2 (0.6) | NA |
| Serekunda, The Gambia | Aug 2007 to Jan 2011 | 8 months to 16 years | 218 (73.9) | 126 (47.0) | 14 (5.0) | 14 (28.0) | 159 (55.6) | 1 (2.0) |
| Keneba, The Gambia | Nov 2009 to Apr 2012 | 4 months to 5 years | NA | NA | NA | 3 (50.0) | 2 (33.3) | NA |
| Sabah, Malaysia | Sep 2010 to Nov 2012 | 13 years to 78 years | NA | 4 (36.4) | 1 (5.6) | 12 (66.7) | 8 (44.4) | 2 (10.0) |
| Manhiça, Mozambique | Apr 2006 to Nov 2006 | 2 months to 5 years | 53 (81.5) | 25 (34.3) | 6 (9.2) | 15 (20.6) | 0 (0.0) | 0 (0.0) |
| Manhiça, Mozambique | Sep 2014 to May 2016 | under 10 years | 61 (87.1) | 30 (32.6) | 5 (7.1) | 12 (12.9) | 1 (1.3) | NA |
| Kilimanjaro and Tanga, Tanzania | Feb 2002 to Aug 2002 | All ages | 586 (42.8) | 167 (20.3) | 39 (5.6) | 173 (12.4) | 11 (0.8) | NA |
| Tanga, Tanzania | Jun 2006 to May 2007 | 2 months to 13 years | 308 (38.8) | 338 (48.4) | 63 (7.9) | 40 (5.0) | 12 (1.5) | NA |
| Kampala, Uganda | 2003 to 2008 | 2 years to 15 years | 1 (100.0) | 13 (29.6) | 1 (1.4) | NA | NA | NA |
| Kampala, Uganda | 2008 to 2013 | 1 year to 11 years | 369 (74.7) | 190 (41.3) | 27 (5.6) | NA | NA | NA |
| Taiz, Yemen | Nov 2002 to Aug 2004 | 6 months to 10 years | NA | 49 (29.0) | 23 (9.1) | 2 (0.8) | 4 (1.6) | 0 (0.0) |
| Macha, Southern Province, Zambia | Mar 2001 to May 2005 | 5 months to 7 years | NA | NA | NA | NA | 11 (10.7) | NA |
|  |  |  |  |  |  |  |  |  |
| **Total** |  |  | **1,829 (53.1)** | **1,001 (36.1)** | **337 (11.4)** | **276 (9.2)** | **230 (6.8)** | **3 (0.8)** |
|  |  |  |  |  |  |  |  |  |
