# Supplementary material for: The impact of delayed treatment of uncomplicated P. falciparum malaria on progression to severe malaria: A systematic review and a pooled multicentre individual-patient meta-analysis
Source: PLoS Med. 2020 Oct 19;17(10):e1003359. doi: 10.1371/journal.pmed.1003359 (PMC7571702; doi:10.1371/journal.pmed.1003359)
Supplement: S3 Table — (DOCX) [file pmed.1003359.s022.docx]

**S3 Table. Study design and matching of included studies**

| Study site | Pre-defined as a case control study? | Was matching used? | What was matched on? |
| --- | --- | --- | --- |
| Cotonou, Benin | NO | NO | NA |
| Farafenni, The Gambia | YES | YES | Age and Sex and season |
| Serekunda, The Gambia | NO | NO | NA |
| Keneba, The Gambia | NO | NO | NA |
| Sabah, Malaysia | NO | NO | NA |
| Manhiça, Mozambique 2006 | YES | YES | Sex and age |
| Manhiça, Mozambique 2014-2016 | YES | YES | Sex, age, and parasitaemia |
| Kilimanjaro and Tanga, Tanzania | NO | NO | NA |
| Tanga, Tanzania 2006-2007 | NO | NO | NA |
| Kampala, Uganda 2003-2008 | NO | NO | NA |
| Kampala, Uganda 2008-2013 | NO | NO | NA |
| Taiz, Yemen | YES | YES | Age (frequency matched) |
| Southern Zambia, Zambia | YES (1 UM and 2 SM groups) | NO | Age, sex and date of admission |
| London, UK | NO | NO | NA |
