# Supplementary material for: The impact of delayed treatment of uncomplicated P. falciparum malaria on progression to severe malaria: A systematic review and a pooled multicentre individual-patient meta-analysis
Source: PLoS Med. 2020 Oct 19;17(10):e1003359. doi: 10.1371/journal.pmed.1003359 (PMC7571702; doi:10.1371/journal.pmed.1003359)
Supplement: S4 Table — Table includes data from 415 individuals aged 16 or over (median age = 35, IQR = 27–46) recruited in Northwick Park Hospital (London, UK) between April 1991 and May 2006 [36]. Table denotes frequencies and percentages with UM and SM. Percentages with a given phenotype amongst severe cases omit missing values for that phenotype. Data for RDS and prostration were not collected. Mortality during admission was estimated amongst SM cases. This study was not used in the pooled analysis but was used to compare IPD analysis findings to those from a setting with high access to care. IPD, individual-participant data; RDS, respiratory distress syndrome; SM, severe malaria; UM, uncomplicated malaria. (DOCX) [file pmed.1003359.s023.docx]

**S4 Table. UK study of severe malaria**. Table includes data from 415 individuals aged 16 or over (median age=35, IQR=27-46) recruited in Northwick Park Hospital (London, UK) between April 1991 and May 2006 [36]. Table denotes frequencies and percentages with uncomplicated and severe malaria. Percentages with a given phenotype among severe cases omit missing values for that phenotype. Data for respiratory distress and prostration was not collected. Mortality during admission was estimated among severe malaria cases. This study was not used in the pooled analysis but was used to compare IPD analysis findings to those from a setting with high access to care.

|  | **N** | **%** |
| --- | --- | --- |
|  |  |  |
| **Uncomplicated Malaria** | 246/415 | 59.3 |
| **Severe Malaria** | 169/415 | 40.7 |
|  |  |  |
| **Severe Malarial Anaemia** | 4/168 | 2.4 |
| **Respiratory Distress Syndrome** | NA | NA |
| **Cerebral Malaria** | 7/41 | 17.1 |
| **Prostration** | NA | NA |
| **Hyperlactataemia** | 3/39 | 7.7 |
| **Hypoglycaemia** | 1/132 | 0.8 |
| **Jaundice** | 60/169 | 35.5 |
| **Hyperparasitaemia** | 66/169 | 39.1 |
| **Renal Impairment** | 74/169 | 43.8 |
|  |  |  |
| **Death** | 4/169 | 2.4 |
|  |  |  |
