# Supplementary material for: The impact of delayed treatment of uncomplicated P. falciparum malaria on progression to severe malaria: A systematic review and a pooled multicentre individual-patient meta-analysis
Source: PLoS Med. 2020 Oct 19;17(10):e1003359. doi: 10.1371/journal.pmed.1003359 (PMC7571702; doi:10.1371/journal.pmed.1003359)
Supplement: S5 Table — Table includes study site, study period, age ranges included, and frequencies of UM and SM for 3 age groups (<5 years, 5 to <15 years, and 15+ years). Age was missing for 1 SM and 5 UM cases. SM, severe malaria; UM, uncomplicated malaria. (DOCX) [file pmed.1003359.s024.docx]

**S5 Table. Frequencies of uncomplicated and severe malaria by age group.** Table includes study site, study period, age ranges included, and frequencies of uncomplicated and severe malaria for three age groups (<5 years, 5 to <15 years and 15+ years). Age was missing for 1 severe and 5 uncomplicated malaria cases.

|  |  |  |  | **Uncomplicated Malaria** | | | |  | **Severe Malaria** | | | |
| --- | --- | --- | --- | --- | --- | --- | --- | --- | --- | --- | --- | --- |
| **Study site** | **Years** | **Age range** |  | **<5 years** | **5 to <15 years** | **15+ years** | **Total** |  | **<5 years** | **5 to <15 years** | **15+ years** | **Total** |
| Cotonou, Benin | Apr 2009 to Aug 2009 | 4 months to 14 years |  | 12 | 34 | 0 | **46** |  | 36 | 8 | 0 | **44** |
| Farafenni, The Gambia | Sept 2002 to Dec 2002 | 1 months to 10 years |  | 125 | 14 | 0 | **139** |  | 271 | 50 | 0 | **321** |
| Serekunda, The Gambia | Aug 2007 to Jan 2011 | 8 months to 16 years |  | 94 | 252 | 14 | **360** |  | 169 | 120 | 6 | **295** |
| Keneba, The Gambia | Nov 2009 to Apr 2012 | 4 months to 5 years |  | 27 | 4 | 0 | **31** |  | 5 | 1 | 0 | **6** |
| Sabah, Malaysia | Sept 2010 to Nov 2012 | 13 years to 78 years |  | 0 | 16 | 159 | **175** |  | 0 | 1 | 19 | **20** |
| Manhiça, Mozambique | Apr 2006 to Nov 2006 | 2 months to 5 years |  | 63 | 0 | 0 | **63** |  | 73 | 0 | 0 | **73** |
| Manhiça, Mozambique | Sept 2014 to May 2016 | under 10 years |  | 34 | 21 | 0 | **55** |  | 66 | 27 | 0 | **93** |
| Kilimanjaro and Tanga, Tanzania | Feb 2002 to Aug 2002 | All ages |  | 1,436 | 496 | 1,006 | **2,941** |  | 1,030 | 159 | 209 | **1,399** |
| Tanga, Tanzania | Jun 2006 to May 2007 | 2 months to 13 years |  | 1,257 | 111 | 0 | **1,368** |  | 753 | 45 | 0 | **798** |
| Kampala, Uganda | 2003 to 2008 | 2 years to 15 years |  | 23 | 67 | 0 | **90** |  | 40 | 45 | 0 | **85** |
| Kampala, Uganda | 2008 to 2013 | 1 year to 11 years |  | NA | NA | NA | **NA** |  | 392 | 102 | 0 | **494** |
| Taiz, Yemen | Nov 2002 to Aug 2004 | 6 months to 10 years |  | 265 | 180 | 0 | **445** |  | 183 | 70 | 0 | **253** |
| Macha, Southern Province, Zambia | Mar 2001 to May 2005 | 5 months to 7 years |  | 64 | 1 | 0 | **67** |  | 108 | 0 | 0 | **108** |
|  |  |  |  |  |  |  |  |  |  |  |  |  |
| **Total** |  |  |  | 3,400 | 1,196 | 1,179 | **5,780** |  | 3,126 | 628 | 234 | **3,989** |
|  |  |  |  |  |  |  |  |  |  |  |  |  |
