# Supplementary material for: The impact of delayed treatment of uncomplicated P. falciparum malaria on progression to severe malaria: A systematic review and a pooled multicentre individual-patient meta-analysis
Source: PLoS Med. 2020 Oct 19;17(10):e1003359. doi: 10.1371/journal.pmed.1003359 (PMC7571702; doi:10.1371/journal.pmed.1003359)
Supplement: S6 Table — CM, cerebral malaria; HG, Hypoglycaemia; HL, Hyperlactataemia or Acidosis; HP, Hyperparasitaemia; JN, Jaundice; PRO, Prostration; RDS, respiratory distress syndrome; RI, Renal Impairment SM, severe malaria; SMA, severe malarial anaemia. (DOCX) [file pmed.1003359.s025.docx]

**S6 Table. Pairwise Phi coefficients between different severe malaria phenotypes** **among severe cases.** Definitions: PRO= Prostration; SMA= Severe malarial anaemia; HL= Hyperlactataemia or Acidosis; RDS= Respiratory distress syndrome; CM= Cerebral malaria; HG= Hypoglycaemia; JN= Jaundice; HP=Hyperparasitaemia; RI=Renal Impairment.

|  | **CM** | **SMA** | **RDS** | **PRO** | **HL** | **HG** | **HP** | **RI** | **JN** |
| --- | --- | --- | --- | --- | --- | --- | --- | --- | --- |
| **CM** | 1.00 |  |  |  |  |  |  |  |  |
| **SMA** | -0.25 | 1.00 |  |  |  |  |  |  |  |
| **RDS** | 0.11 | -0.14 | 1.00 |  |  |  |  |  |  |
| **PRO** | 0.40 | -0.39 | 0.11 | 1.00 |  |  |  |  |  |
| **HL** | 0.03 | 0.01 | 0.24 | -0.003 | 1.00 |  |  |  |  |
| **HG** | 0.01 | -0.01 | 0.04 | 0.02 | 0.10 | 1.00 |  |  |  |
| **HP** | 0.02 | -0.18 | 0.21 | 0.06 | 0.10 | -0.03 | 1.00 |  |  |
| **RI** | -0.03 | -0.06 | -0.08 | -0.21 | 0.07 | -0.03 | -0.03 | 1.00 |  |
| **JN** | -0.02 | -0.12 | -0.03 | 0.09 | -0.03 | -0.06 | 0.06 | 0.06 | 1.00 |
