# Supplementary material for: The impact of delayed treatment of uncomplicated P. falciparum malaria on progression to severe malaria: A systematic review and a pooled multicentre individual-patient meta-analysis
Source: PLoS Med. 2020 Oct 19;17(10):e1003359. doi: 10.1371/journal.pmed.1003359 (PMC7571702; doi:10.1371/journal.pmed.1003359)
Supplement: S7 Table — Duration of severe symptoms obtained from 5 studies for 1,323 individuals. The median delay to admission in days after onset of severe symptoms and the difference between reported onset of severe symptoms and onset of uncomplicated disease are presented. (DOCX) [file pmed.1003359.s026.docx]

**S7 Table. Duration of severe symptoms****.** Duration of severe symptoms obtained from 5 studies for 1,323 individuals. The median delay to admission in days after onset of severe symptoms and the difference between reported onset of severe symptoms and onset of uncomplicated disease are presented.

| Severe symptom | Source | N | Median delay in days after onset of severe symptoms (IQR) | Median difference in days between onset of fever/illness and severe symptoms (IQR) |
| --- | --- | --- | --- | --- |
|  |  |  |  |  |
| Coma | Uganda 2003-2008 Uganda 2008-2013 | 344 | 0.71(0.27, 1.04) | 2.46 (1.58, 3.42) |
| Unconsciousness | Gambia 2002  Uganda 2008-2013 | 501 | 0.00 (0.00, 0.00) | 3.00 (2.00, 4.00) |
| Convulsions | Gambia 2002 | 453 | 0.00(0.00, 0.00) | 3.00 (2.00, 4.00) |
| Difficulty breathing | Gambia 2002 Uganda 2008-2013 | 947 | 0.00 (0.00, 0.00) | 3.00 (2.00, 4.00) |
| Fast breathing | Gambia 2002 | 453 | 0.00(0.00, 1.00) | 3.00 (1.00, 3.00) |
| Respiratory distress | Mozambique 2006 Mozambique 2014 | 292 | 0.00 (0.00, 0.00) | 1.00 (1.00, 2.00) |
