# Supplementary material for: The impact of delayed treatment of uncomplicated P. falciparum malaria on progression to severe malaria: A systematic review and a pooled multicentre individual-patient meta-analysis
Source: PLoS Med. 2020 Oct 19;17(10):e1003359. doi: 10.1371/journal.pmed.1003359 (PMC7571702; doi:10.1371/journal.pmed.1003359)
Supplement: S8 Table — RRs and associated 95% CI shown were obtained using a GEE model, allowing for correlation of observations within studies. Models are shown for children (<15 years). GEE, generalised estimating equations; RR, risk ratio (DOCX) [file pmed.1003359.s027.docx]

**S8 Table.** **Risk ratios for presentation with severe disease phenotypes.** Risk ratios and associated 95%CI shown were obtained using a generalized estimating equations (GEE) model, allowing for correlation of observations within studies. Models are shown for children (<15 years).

| **Children (<15 years)** | | **Risk Ratios (95 CI%)** | | | |
| --- | --- | --- | --- | --- | --- |
|  |  | **Any**  **severe** | **Severe malarial anaemia** | **Cerebral**  **malaria** | **Respiratory distress** |
| **N** (incl 3,277 UM cases) | | 6,985 | 5,051 | 4,014 | 4,222 |
| **Duration of illness (vs. within 1 day)** | **>1:≤2** | 1.06 (0.95, 1.18) | 1.18 (0.90, 1.54) | 1.05 (0.84, 1.31) | 1.08 (0.85, 1.37) |
|  | **>2:≤3** | 1.12 (1.01, 1.24) | 1.75 (1.33, 2.30) | 0.99 (0.80, 1.23) | 1.13 (0.89, 1.42) |
|  | **>3:≤4** | 1.16 (1.04, 1.30) | 1.97 (1.48, 2.63) | 0.98 (0.77, 1.24) | 1.18 (0.92, 1.51) |
|  | **>4:≤5** | 1.15 (1.02, 1.29) | 2.02 (1.50, 2.73) | 0.94 (0.72, 1.23) | 1.20 (0.92, 1.56) |
|  | **>5:≤6** | 1.19 (1.04, 1.37) | 2.25 (1.63, 3.11) | 0.86 (0.58, 1.26) | 1.06 (0.74, 1.50) |
|  | **>6:≤7** | 1.10 (0.98, 1.24) | 1.99 (1.48, 2.68) | 0.91 (0.69, 1.20) | 1.16 (0.89, 1.52) |
|  | **>7** | 1.24 (1.09, 1.41) | 2.43 (1.76, 3.34) | 1.07 (0.79, 1.43) | 1.21 (0.90, 1.63) |
| **Age (years)** | | 0.98 (0.96, 0.99) | 0.94 (0.91, 0.96) | 1.01 (0.98, 1.03) | 0.93 (0.91, 0.96) |
