# Supplementary material for: The impact of delayed treatment of uncomplicated P. falciparum malaria on progression to severe malaria: A systematic review and a pooled multicentre individual-patient meta-analysis
Source: PLoS Med. 2020 Oct 19;17(10):e1003359. doi: 10.1371/journal.pmed.1003359 (PMC7571702; doi:10.1371/journal.pmed.1003359)
Supplement: S9 Table — Sensitivity analysis with (A) exclusion of multiple phenotypes and (B) mother’s education as a covariate. Age-adjusted ORs (and 95% CIs) for the association between delay to treatment and SMA, RDS, and CM in children aged under 15. Age-adjusted ORs were obtained from a mixed-effects logistic regression, with receiving treatment within 1 day of symptom onset being the reference category. For sensitivity analysis A, cases with the phenotype of interest and prostration were not excluded because of prostration being a consequence of other symptoms (for instance, large overlap between CM and prostration). For sensitivity analysis B, low mother’s education was defined as not having completed at least primary education. The mothers of 1,782 (38.0%) children did not complete primary education. Mother’s education was quantified in 4 of the studies from Yemen (n = 781), The Gambia (Farafenni; n = 447), Tanzania 2002–2003 (n = 1,309), and Tanzania 2006–2007 (n = 2,157). CM, cerebral malaria; OR, odds ratio; RDS, respiratory distress syndrome; SMA, severe malarial anaemia. (DOCX) [file pmed.1003359.s028.docx]

**S9 Table. Sensitivity analysis with A) exclusion of multiple phenotypes and B) mother’s education as a covariate.** Age-adjusted ORs (and 95%CIs) for the association between delay to treatment and severe malarial anaemia (SMA), respiratory distress syndrome (RDS), and cerebral malaria (CM) in children aged under 15. Age-adjusted ORs were obtained from a mixed-effects logistic regression, with receiving treatment within 1 day of symptom onset being the reference category. For sensitivity analysis A, cases with the phenotype of interest and prostration were not excluded, due to prostration being a consequence of other symptoms (e.g. large overlap between CM and prostration). For sensitivity analysis B, low mother’s education was defined as not having completed at least primary education. The mothers of 1,782 (38.0%) children did not complete primary education. Mother’s education was quantified in four of the studies from Yemen (n=781), Gambia (Farafenni; n=447), Tanzania 2002-2003 (n=1,309) and Tanzania 2006-2007 (n=2,157).

|  |  |  | **Sensitivity analysis A** | | |  | **Sensitivity analysis B** | | |  | **Main analysis** | | |
| --- | --- | --- | --- | --- | --- | --- | --- | --- | --- | --- | --- | --- | --- |
|  | **Variable** |  | **No other phenotype** | | |  | **Including education** | | |  |  |  |  |
|  |  |  | **OR** | **95% CI** | |  | **OR** | **95% CI** | |  | **OR** | **95% CI** | |
| **SMA** | **Delay in days vs. ≤ 1day** | **>1:≤2** | 1.24 | 0.76 | 2.03 |  | 1.54 | 0.85 | 2.79 |  | 1.34 | 0.90 | 1.98 |
|  |  | **>2:≤3** | 2.42 | 1.52 | 3.83 |  | 3.13 | 1.78 | 5.49 |  | 2.79 | 1.92 | 4.06 |
|  |  | **>3:≤4** | 3.01 | 1.86 | 4.86 |  | 3.89 | 2.19 | 6.91 |  | 3.60 | 2.44 | 5.32 |
|  |  | **>4:≤5** | 3.60 | 2.18 | 5.95 |  | 4.71 | 2.60 | 8.54 |  | 3.83 | 2.53 | 5.79 |
|  |  | **>5:≤6** | 5.00 | 2.88 | 8.69 |  | 5.27 | 2.75 | 10.09 |  | 4.61 | 2.88 | 7.36 |
|  |  | **>6:≤7** | 3.48 | 2.09 | 5.78 |  | 4.67 | 2.56 | 8.51 |  | 3.76 | 2.48 | 5.71 |
|  |  | **>7** | 5.23 | 3.05 | 8.96 |  | 7.10 | 3.82 | 13.23 |  | 5.46 | 3.49 | 8.53 |
|  | **Age (years)** | | 0.91 | 0.87 | 0.96 |  | 0.90 | 0.86 | 0.94 |  | 0.89 | 0.85 | 0.92 |
|  | **Low education** | | NA | NA | NA |  | 1.81 | 1.51 | 2.16 |  | NA | NA | NA |
|  |  |  |  |  |  |  |  |  |  |  |  |  |  |
| **RDS** | **Delay in days vs. ≤ 1day** | **>1:≤2** | 1.09 | 0.59 | 2.01 |  | 0.98 | 0.58 | 1.67 |  | 1.13 | 0.77 | 1.66 |
|  |  | **>2:≤3** | 1.03 | 0.57 | 1.85 |  | 1.27 | 0.77 | 2.08 |  | 1.22 | 0.85 | 1.76 |
|  |  | **>3:≤4** | 1.08 | 0.57 | 2.06 |  | 1.44 | 0.85 | 2.43 |  | 1.31 | 0.88 | 1.95 |
|  |  | **>4:≤5** | 1.19 | 0.60 | 2.35 |  | 1.71 | 0.99 | 2.97 |  | 1.35 | 0.88 | 2.07 |
|  |  | **>5:≤6** | 1.32 | 0.59 | 2.96 |  | 1.36 | 0.70 | 2.67 |  | 1.11 | 0.64 | 1.92 |
|  |  | **>6:≤7** | 1.41 | 0.72 | 2.75 |  | 1.55 | 0.89 | 2.71 |  | 1.28 | 0.83 | 1.97 |
|  |  | **>7** | 1.42 | 0.71 | 2.82 |  | 1.53 | 0.85 | 2.74 |  | 1.39 | 0.87 | 2.24 |
|  | **Age (years)** | | 0.99 | 0.93 | 1.05 |  | 0.95 | 0.90 | 0.99 |  | 0.89 | 0.86 | 0.93 |
|  | **Low education** | | NA | NA | NA |  | 1.74 | 1.39 | 2.16 |  | NA | NA | NA |
|  |  |  |  |  |  |  |  |  |  |  |  |  |  |
| **CM** | **Delay in days vs. ≤ 1day** | **>1:≤2** | 1.58 | 0.85 | 2.91 |  | 0.75 | 0.40 | 1.39 |  | 1.11 | 0.73 | 1.69 |
|  |  | **>2:≤3** | 1.06 | 0.58 | 1.93 |  | 0.67 | 0.38 | 1.19 |  | 0.97 | 0.65 | 1.44 |
|  |  | **>3:≤4** | 1.28 | 0.67 | 2.45 |  | 0.47 | 0.24 | 0.91 |  | 0.94 | 0.60 | 1.47 |
|  |  | **>4:≤5** | 1.19 | 0.56 | 2.52 |  | 0.70 | 0.35 | 1.41 |  | 0.86 | 0.52 | 1.45 |
|  |  | **>5:≤6** | 0.67 | 0.18 | 2.43 |  | 0.76 | 0.32 | 1.85 |  | 0.71 | 0.33 | 1.53 |
|  |  | **>6:≤7** | 0.98 | 0.44 | 2.17 |  | 0.81 | 0.40 | 1.64 |  | 0.81 | 0.48 | 1.37 |
|  |  | **>7** | 1.23 | 0.53 | 2.87 |  | 1.03 | 0.47 | 2.24 |  | 1.15 | 0.64 | 2.07 |
|  | **Age (years)** | | 1.07 | 1.00 | 1.14 |  | 1.13 | 1.07 | 1.20 |  | 1.01 | 0.97 | 1.06 |
|  | **Low education** | | NA | NA | NA |  | 1.73 | 1.27 | 2.37 |  | NA | NA | NA |
|  |  |  |  |  |  |  |  |  |  |  |  |  |  |
