# Supplementary material for: The impact of delayed treatment of uncomplicated P. falciparum malaria on progression to severe malaria: A systematic review and a pooled multicentre individual-patient meta-analysis
Source: PLoS Med. 2020 Oct 19;17(10):e1003359. doi: 10.1371/journal.pmed.1003359 (PMC7571702; doi:10.1371/journal.pmed.1003359)
Supplement: S10 Table — Age-adjusted ORs (and 95% CIs) for the association between duration of illness and SMA, RDS, and CM in children under 15. Age-adjusted ORs were obtained from a mixed-effects logistic regression, with receiving treatment within 1 day of symptom onset being the reference category. Models include an interaction between transmission intensity and the effect of duration of illness on a phenotype. Transmission intensity was categorised into low (PfPR2–10 < 10%), moderate (PfPR2–10 of 10% to <35%), and high (PfPR2–10 ≥ 35%). CM, cerebral malaria; OR, odds ratio; RDS, respiratory distress syndrome; SMA, severe malarial anaemia. (DOCX) [file pmed.1003359.s029.docx]

|  |  | **Low**  **transmission** | | |  | **Moderate transmission** | | |  | **High**  **transmission** | | |
| --- | --- | --- | --- | --- | --- | --- | --- | --- | --- | --- | --- | --- |
| **Severe malarial anaemia** | | **OR** | **95%CI** | |  | **OR** | **95%CI** | |  | **OR** | **95%CI** | |
| **Duration of illness (vs. within 1 day)** | **>1:≤2** | 3.22 | 1.08 | 9.62 |  | 1.30 | 0.12 | 13.91 |  | 1.21 | 0.11 | 13.64 |
|  | **>2:≤3** | 4.46 | 1.54 | 12.89 |  | 3.71 | 0.38 | 36.69 |  | 1.76 | 0.17 | 18.20 |
|  | **>3:≤4** | 5.49 | 1.85 | 16.33 |  | 5.97 | 0.56 | 63.08 |  | 1.61 | 0.15 | 17.77 |
|  | **>4:≤5** | 7.19 | 2.38 | 21.73 |  | 7.61 | 0.69 | 84.42 |  | 1.18 | 0.10 | 13.68 |
|  | **>5:≤6** | 6.26 | 1.85 | 21.21 |  | 8.68 | 0.60 | 126.13 |  | 2.17 | 0.15 | 32.11 |
|  | **>6:≤7** | 10.54 | 3.49 | 31.86 |  | 4.64 | 0.41 | 52.21 |  | 1.51 | 0.13 | 17.49 |
|  | **>7** | 13.06 | 4.34 | 39.31 |  | 6.37 | 0.56 | 73.01 |  | 1.80 | 0.14 | 23.62 |
|  |  |  |  |  |  |  |  |  |  |  |  |  |
| **Respiratory distress** | |  |  |  |  |  |  |  |  |  |  |  |
| **Duration of illness (vs. within 1 day)** | **>1:≤2** | 0.96 | 0.55 | 1.66 |  | 1.50 | 0.35 | 6.35 |  | 1.70 | 0.36 | 8.12 |
|  | **>2:≤3** | 0.88 | 0.52 | 1.49 |  | 2.04 | 0.51 | 8.13 |  | 1.60 | 0.35 | 7.34 |
|  | **>3:≤4** | 0.53 | 0.29 | 0.99 |  | 3.51 | 0.74 | 16.71 |  | 1.46 | 0.26 | 8.09 |
|  | **>4:≤5** | 0.69 | 0.37 | 1.31 |  | 4.16 | 0.82 | 21.05 |  | 1.00 | 0.16 | 6.24 |
|  | **>5:≤6** | 0.54 | 0.23 | 1.28 |  | 3.71 | 0.44 | 31.42 |  | 0.71 | 0.06 | 8.29 |
|  | **>6:≤7** | 0.70 | 0.37 | 1.33 |  | 3.13 | 0.60 | 16.23 |  | 1.47 | 0.25 | 8.71 |
|  | **>7** | 1.12 | 0.60 | 2.06 |  | 1.64 | 0.28 | 9.68 |  | 1.06 | 0.11 | 9.79 |
|  |  |  |  |  |  |  |  |  |  |  |  |  |
| **Cerebral malaria** | |  |  |  |  |  |  |  |  |  |  |  |
| **Duration of illness (vs. within 1 day)** | **>1:≤2** | 0.96 | 0.44 | 2.09 |  | 1.29 | 0.22 | 7.56 |  | 0.37 | 0.03 | 4.28 |
|  | **>2:≤3** | 0.66 | 0.31 | 1.40 |  | 1.49 | 0.27 | 8.20 |  | 0.38 | 0.04 | 3.77 |
|  | **>3:≤4** | 0.58 | 0.25 | 1.33 |  | 1.59 | 0.24 | 10.61 |  | 0.40 | 0.03 | 4.67 |
|  | **>4:≤5** | 0.61 | 0.25 | 1.50 |  | 1.45 | 0.18 | 11.86 |  | 0.26 | 0.02 | 3.97 |
|  | **>5:≤6** | 0.39 | 0.10 | 1.59 |  | 2.12 | 0.09 | 51.83 |  | 0.15 | 0.00 | 9.64 |
|  | **>6:≤7** | 0.83 | 0.35 | 1.99 |  | 1.18 | 0.15 | 9.36 |  | 0.07 | 0.00 | 1.96 |
|  | **>7** | 1.31 | 0.51 | 3.34 |  | 0.98 | 0.10 | 9.76 |  | 0.31 | 0.01 | 10.08 |

**S10 Table. Delay to treatment and severe disease phenotypes for different malaria transmission intensity levels in children**. Age-adjusted ORs (and 95%CIs) for the association between duration of illness and Severe Malarial Anaemia, Respiratory Distress, and Cerebral Malaria in children under 15. Age-adjusted ORs were obtained from a mixed-effects logistic regression, with receiving treatment within 1 day of symptom onset being the reference category. Models include an interaction between transmission intensity and the effect of duration of illness on a phenotype. Transmission intensity was categorised into low (*Pf*PR_2-10_ <10%), moderate (*Pf*PR_2-10_ of 10 to <35%), and high (*Pf*PR_2-10_ ≥35%).
