# Supplementary material for: The impact of delayed treatment of uncomplicated P. falciparum malaria on progression to severe malaria: A systematic review and a pooled multicentre individual-patient meta-analysis
Source: PLoS Med. 2020 Oct 19;17(10):e1003359. doi: 10.1371/journal.pmed.1003359 (PMC7571702; doi:10.1371/journal.pmed.1003359)
Supplement: S11 Table — Case fatality in UM and SM groups. Table denotes the number of individuals with available mortality status during admission and the number (and %) of deaths amongst those. SM, severe malaria; UM, uncomplicated malaria. (DOCX) [file pmed.1003359.s030.docx]

**S11 Table. Mortality by severe phenotype.** Case fatality in uncomplicated and severe malaria groups. Table denotes the number of individuals with available mortality status during admission and the number (and %) of deaths among those.

| ***Severity group*** | **N** | **Deaths** | **Mortality (%)** |
| --- | --- | --- | --- |
|  |  |  |  |
| ***Uncomplicated*** | 2,921 | 29 | 1.0 |
| ***Any severe*** | 3,811 | 316 | 8.3 |
|  |  |  |  |
| ***Severe anaemia*** | 1,887 | 133 | 7.0 |
| ***Prostration*** | 1,710 | 218 | 12.7 |
| ***Hyperlactataemia/Acidosis*** | 992 | 129 | 13.0 |
| ***Respiratory distress*** | 949 | 165 | 17.4 |
| ***Cerebral malaria*** | 776 | 152 | 19.6 |
| ***Jaundice*** | 270 | 19 | 7.0 |
| ***Hypoglycaemia*** | 324 | 59 | 18.2 |
| ***Hyperparasitaemia*** | 191 | 8 | 4.2 |
| ***Renal Impairment*** | 3 | 0 | 0.0 |
|  |  |  |  |
| ***Total*** | 6,677 | 341 | 5.1 |
